# Author Correction: APC/C-dependent degradation of Spd2 regulates centrosome asymmetry in *Drosophila* neural stem cells

**DOI:** 10.1038/s44319-024-00080-2

**Published:** 2024-02-16

**Authors:** Francesco Meghini, Torcato Martins, Qian Zhang, Nicolas Loyer, Michelle Trickey, Yusanjiang Abula, Hiroyuki Yamano, Jens Januschke, Yuu Kimata

**Affiliations:** 1 Department of Genetics, University of Cambridge, Cambridge, UK; 2 Department of Medical Sciences, Institute of Biomedicine-iBiMED, University of Aveiro, Aveiro, Portugal; 3 School of Life Science and Technology, ShanghaiTech University, Shanghai, China; 4 School of Life Science, University of Dundee, Dundee, UK; 5 UCL Cancer Institute, University College London, London, UK

## Abstract

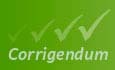

C**orrection to**: *EMBO Reports* (2023) 24:e55607. 10.15252/embr.202255607 | Published online 28 February 2023

**There is an error in the Acknowledgements section of this article relating to a grant number for the Natural Science Foundation of China attributed to (Y.K.)**

The Acknowledgements section is corrected from:

We thank Dr David Glover and his lab members for reagents, discussions and access to their spinning disc microscope, ShanghaiTech University Imaging Facility for access to microscopes, Drs Jordan Raff and Renata Basto for reagents and discussions, Dr Wei Wu at Shanghai Fly Center, Dr Simon Collier at Cambridge Fly facility, and Drs Jingnan Liu and Ji‐long Liu for their help with maintenance and transporting fly stocks, Bloomington *Drosophila* Stock Center, Cambridge Fly Facility and Shanghai Fly Center, the Vienna *Drosophila* RNA Center (VDRC) and Cambridge and China *Drosophila* research communities for fly stocks, *Drosophila* colleagues at ShanghaiTech University and the University of Cambridge for discussion, sharing information and reagents. We thank all the Kimata lab members for cooperation and discussion. This work was supported by the ShanghaiTech University start‐up grant (2018F0202‐000‐06) and the Cancer Research UK Career Development Fellowship (CRUK‐Al2874) and the Natural Science Foundation of China (3217046) to Y.K., HORIZON EUROPE European Research Council Marie Curie Fellowship (655297) to T.M., and the Wellcome Trust (205150/Z/16/Z), Marie Curie Cancer Care and Worldwide Cancer Research (formerly known as AICR) grant (07‐0062) to H.Y. Work in J.J.‘s lab was supported by the grant (BB/V001353/1) from Biotechnology and Biological Sciences Research Council.

To (see changes in bold)

We thank Dr David Glover and his lab members for reagents, discussions and access to their spinning disc microscope, ShanghaiTech University Imaging Facility for access to microscopes, Drs Jordan Raff and Renata Basto for reagents and discussions, Dr Wei Wu at Shanghai Fly Center, Dr Simon Collier at Cambridge Fly facility, and Drs Jingnan Liu and Ji‐long Liu for their help with maintenance and transporting fly stocks, Bloomington *Drosophila* Stock Center, Cambridge Fly Facility and Shanghai Fly Center, the Vienna *Drosophila* RNA Center (VDRC) and Cambridge and China *Drosophila* research communities for fly stocks, *Drosophila* colleagues at ShanghaiTech University and the University of Cambridge for discussion, sharing information and reagents. We thank all the Kimata lab members for cooperation and discussion. This work was supported by the ShanghaiTech University start‐up grant (2018F0202‐000‐06) and the Cancer Research UK Career Development Fellowship (CRUK‐Al2874) and the Natural Science Foundation of China (**32170746**) to Y.K., HORIZON EUROPE European Research Council Marie Curie Fellowship (655297) to T.M., and the Wellcome Trust (205150/Z/16/Z), Marie Curie Cancer Care and Worldwide Cancer Research (formerly known as AICR) grant (07‐0062) to H.Y. Work in J.J.‘s lab was supported by the grant (BB/V001353/1) from Biotechnology and Biological Sciences Research Council.

This change does not affect the text or interpretation of the article.

