# Supplementary material for: APC/C‐dependent degradation of Spd2 regulates centrosome asymmetry in Drosophila neural stem cells
Source: EMBO Rep. 2023 Feb 28;24(4):e55607. doi: 10.15252/embr.202255607 (PMC10074082; doi:10.15252/embr.202255607)
Supplement: Supplementary file 1 — Expanded View Figures PDF [file EMBR-24-e55607-s014.pdf]

Expanded View Figures

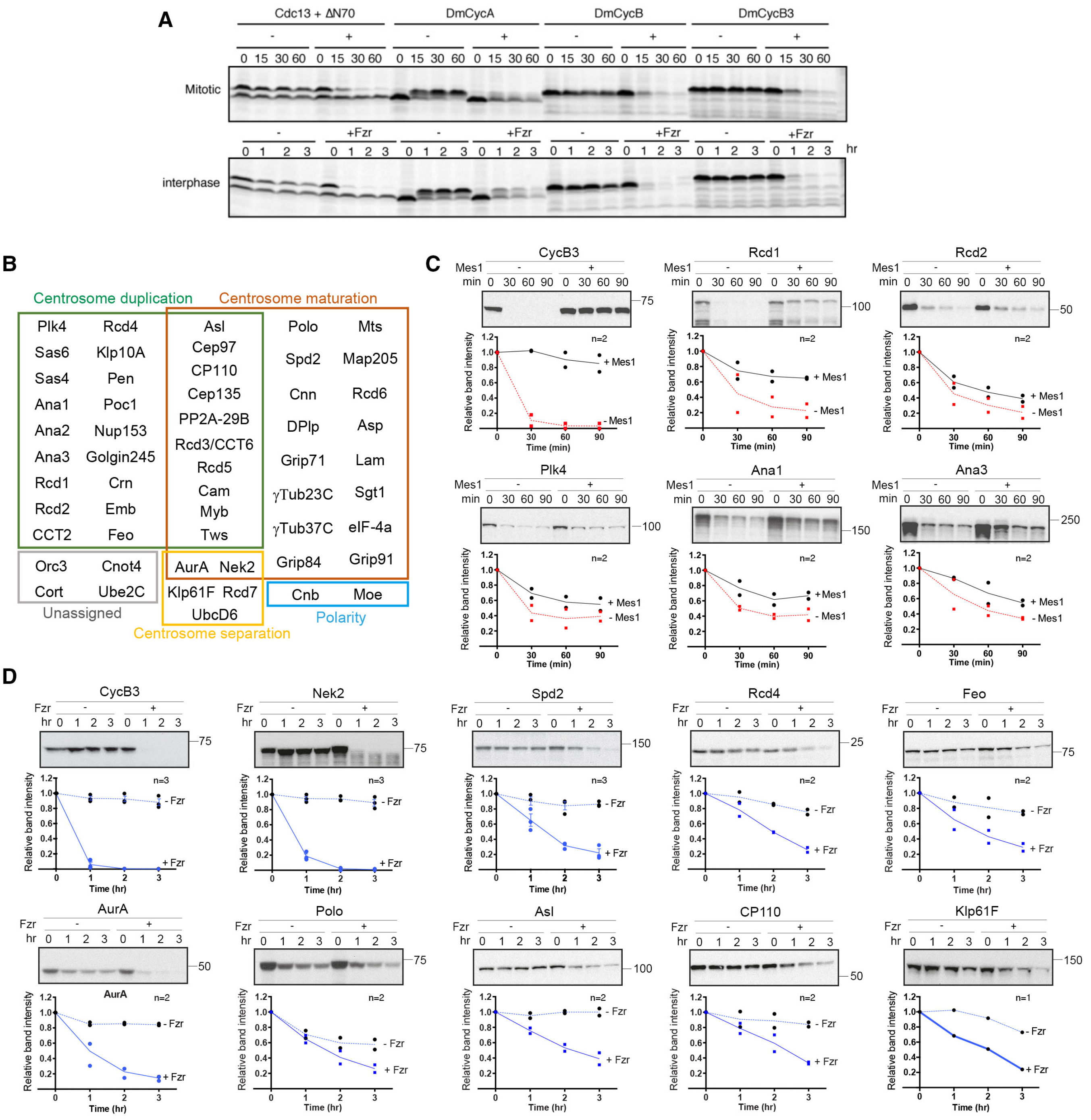

Figure EV1.

Figure EV1. The *in vitro* degradation screen identified putative centrosomal APC/C targets in *Drosophila*.

A Cell-free reconstituted APC/C-dependent degradation assays using mitotic and interphase *Xenopus leavis* egg extracts. ^{35}S-labelled substrate proteins were translated *in vitro* and were used to test their degradation. *S. pombe* mitotic cyclin Cdc13 and its stabilised mutant version ΔN70, which lacks the N-terminal 70 amino acid containing APC/C-recognition motifs, were used as positive and negative controls, respectively. All three *Drosophila* mitotic cyclins, Cyclin A (DmCycA), Cyclin B (DmCycB) and Cyclin B3 (DmCycB3), were rapidly degraded upon APC/C activation in mitotic (upper panel) and interphase egg extracts (lower panel) at similar kinetics to Cdc13.

B The cohort of *Drosophila* 55 centrosomal proteins for the *in vitro* screen. Candidate centrosomal components are grouped into different functional groups based on previous studies.

C Degradation assays of a control CycB3 and five putative APC/C^{Fzy} substrates, Rcd1, Rcd2, Plk4, Ana1 and Ana3, in mitotic egg extracts in the presence/absence of APC/C inhibitor Mes1 (See Materials and Methods for details). The representative autoradiographs are shown with molecular weight markers (kDa) in top panels, and the signal intensities of the corresponding bands at each time point on the autoradiographs were measured, and means of the relative values are shown in the line graphs, with the individual values indicated by dots, in lower panels. *n*: number of biological replicates.

D Degradation assays of CycB3 and 9 putative APC/C^{CDH1} substrates, Nek2, Spd2, Rcd4, Feo, AurA, Polo, Asl, CP110 and Klp61F, in interphase egg extracts in the presence/absence of the interphase APC/C activator Fzr. The results are presented as in (C). For data with *n* = 3, SD is shown as error bars.

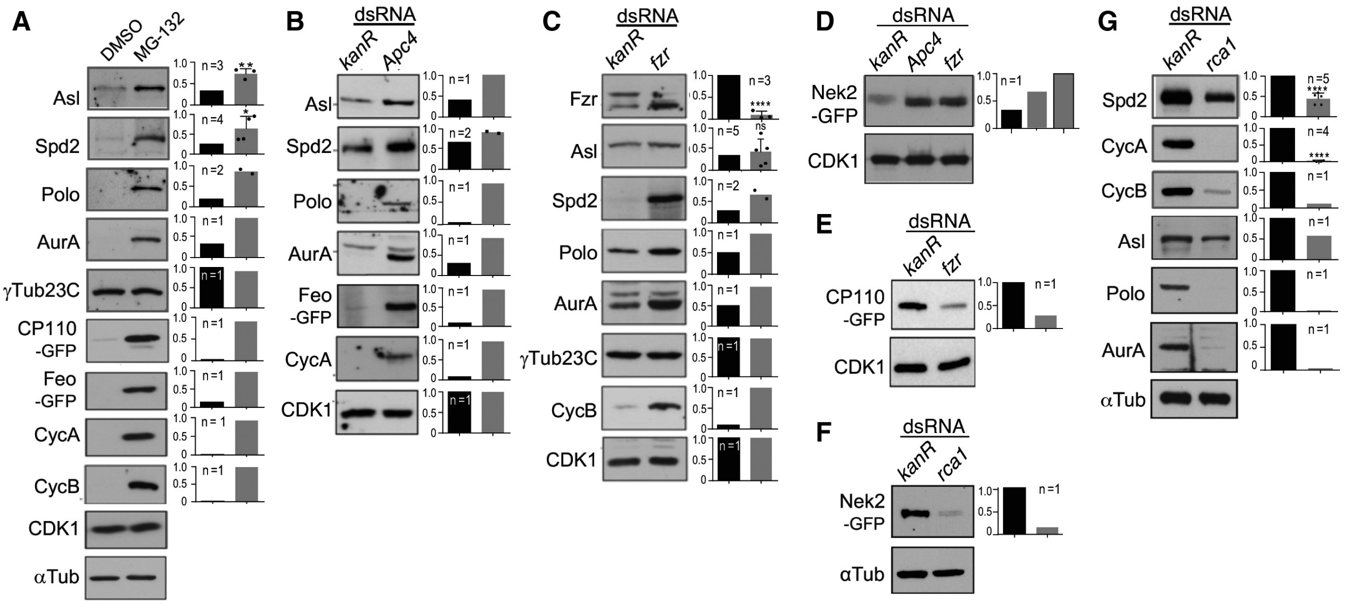

Figure EV2. type="main">APC/C^{Fzr}-dependent regulation of the protein levels of putative centrosomal APC/C substrates in cultured *Drosophila* cells.

A Effects of inhibition of the 26S proteasome by MG-132 on the levels of interphase APC/C substrate candidates in cultured *D-mel.2* cells. Examples of Western blots are presented on the left. The signal intensities of corresponding bands were measured and normalised by those of the loading controls and presented as bar graphs on the right. CDK1 and α-tubulin (αTub). CycA and CycB were used as positive controls. Proteasome inhibition caused the accumulation of endogenous Asl, Spd2, Polo and AurA proteins and transiently transfected CP110-GFP and Feo-GFP but did not affect γ-Tubulin (γTub23C). *P*-values are shown for Asl: 0.0041 (**) and for Sdp2 0.0439 (*).

B, D Effects of RNAi-mediated depletion of an APC/C subunit Apc4 on the levels of interphase candidates in cultured *D.mel-2* cells. dsRNA against the bacterial kanamycine resistant gene (*kanR*) was used as negative control for RNAi. CDK1 was used as the loading control. The accumulation of CycA was detected to confirm APC/C inactivation. Depletion of APC/C subunit Apc4 causes the accumulation of endogenous Asl, Spd2, Polo and AurA proteins and transiently transfected Feo-GFP and Nek2-GFP (D). *P*-values are indicated for Fzr: < 0.0001 (****) and for Asl: 0.6062 (ns).

C–E Effects of RNAi-mediated Fzr depletion on the levels of interphase candidates in cultured *D.mel-2* cells. CDK1 was used as the loading control. Fzr depletion results in the accumulation of endogenous Spd2, Polo and AurA proteins (C), as well as transiently transfected Nek2-GFP (D), while CP110-GFP was reduced (E).

F, G Effects of RNAi-mediated depletion of the APC/C^{Fzr} inhibitor, Rca1, on the levels of interphase candidates in cultured *D.mel-2* cells. αTub was used as the loading control. The reduction of CycA and CycB was detected to confirm the upregulation of APC/C^{Fzr} (G). Rca1 depletion causes the reduction of transiently transfected Nek2-GFP (F), as well as endogenous Spd2, Asl, Polo and AurA proteins (G), in *D.mel-2* cells. *P*-values are shown for Spd2: < 0.0001 (****) and CycA: < 0.0001 (****).

Data information: In all bar graphs in this figure, the individual values ($n = 1$) or their means ($n \geq 2$) are presented with error bars (SD, $n \geq 3$) and dots (individual values, $n \geq 2$). *n*: number of biological replicates. *P*-values were calculated using unpaird two-tailed *t*-test.
Source data are available online for this figure.

 © 2023 The Authors

A

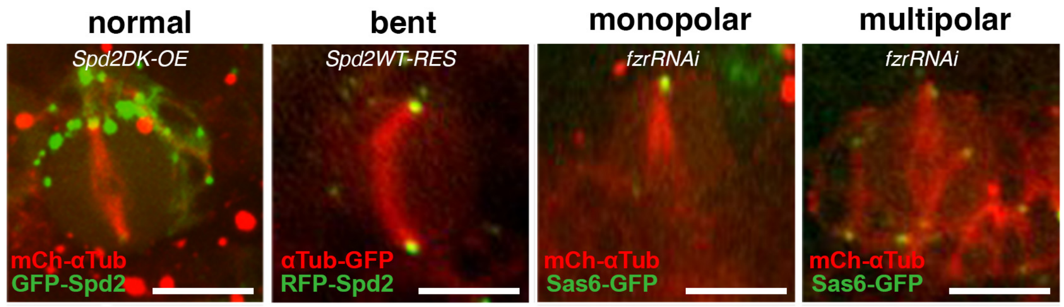

B

line name	bent spindle					transient multipolar spindle					transient monopolar spindle				
	Total (n)	phenotype	%	p-value (chi test)		Total (n)	phenotype	%	p-value (chi test)		Total (n)	phenotype	%	p-value (chi test)	
control	99	1	**1.0%**	-		100	1	**1.0%**	-		100	0	**0.0%**	-	
fzrRNAi	182	15	**8.2%**	0.0125	*	117	19	**16.2%**	0.0001	***	182	10	**5.5%**	0.0170	*
Spd2WT-RES	82	14	**17.1%**	0.0001	***	81	2	**2.5%**	0.4414	ns	81	0	**0.0%**		
Spd2DK-RES	167	25	**15.0%**	0.0002	***	163	16	**9.8%**	0.0048	**	161	0	**0.0%**		
Spd2WT-OE	17	0	**0.0%**	0.6773	ns	15	0	**0.0%**	0.6973	ns	15	0	**0.0%**		
Spd2DK-OE	45	5	**11.1%**	0.0049	**	45	1	**2.2%**	0.5594	ns	45	0	**0.0%**		
Spd2WT-OE+fzrRNAi	140	4	**2.9%**	0.3257	ns	134	4	**3.0%**	0.2989	ns	134	0	**0.0%**		

Figure EV3. Transient abnormal spindle morphologies observed in NB live imaging.

A Examples of normal bipolar spindle and transient abnormal spindle morphologies, bent spindle, monopolar spindle and multipolar spindle observed in mitotic NBs in our time-lapse live imaging. In these experiments, centrosomes and microtubules were visualised using fluorescently labelled centrosomal proteins, Sas6, Fzr or Spd2, and α-Tubulin, which are shown in red and green in this figure. Scale bars: 10 μm.

B The frequency of each of the three abnormal spindle morphologies were quantified. These abnormal spindle morphologies were observed in the NBs only for a few frames (90 fps), and the NBs then recovered normal bipolar spindle before anaphase. While fractions of *fzrRNAi*, *Spd2WT/DK-RES* and *Spd2DK-OE* NBs transiently exhibit these abnormal spindle morphologies, *Spd2WT-OE* NBs did not show these defects. *n*: total number of NB divisions analysed from at least three brains of each line. *P*-values were calculated by Pearson's chi-squared tests by comparing with control.

© 2023 The Authors

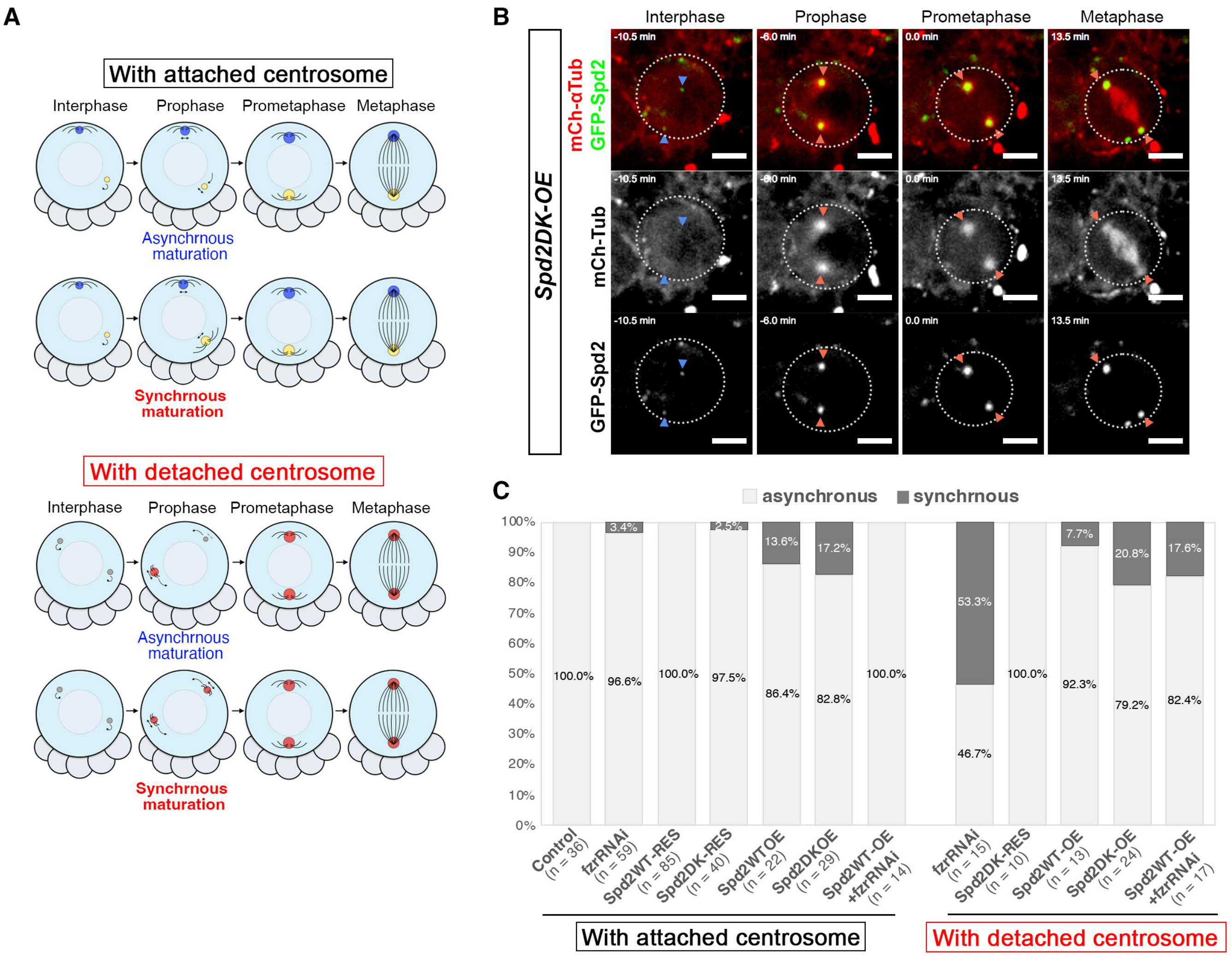

Figure EV4. Asynchronous and synchronous centrosome maturation in larval NBs with increased Spd2 levels.

A A schematic representation of asynchronous and synchronous centrosome maturation observed upon mitotic entry in the NBs in which both centrosomes become inactive and detached from the cortex in interphase due to Spd2 accumulation.

B Selected images from a time-lapse movie of *Spd2DK-OE* NBs in which two centrosomes that were inactive in interphase simultaneously expanded and started nucleating microtubules upon mitotic entry (i.e. centrosome maturation, Movie EV9). Blue arrowheads indicate inactive centrosomes, and red arrowheads indicate activated mitotic centrosomes. White dotted circles outline a NB. Scale bars: 5 µm.

C The frequencies of asynchronous and synchronous centrosome maturation observed upon mitotic entry in NBs of each of the indicated lines, with or without the apical centrosomes being detached in the preceding interphase. *n*: the number of NB divisions analysed from at least three brains of each ilne.

 © 2023 The Authors

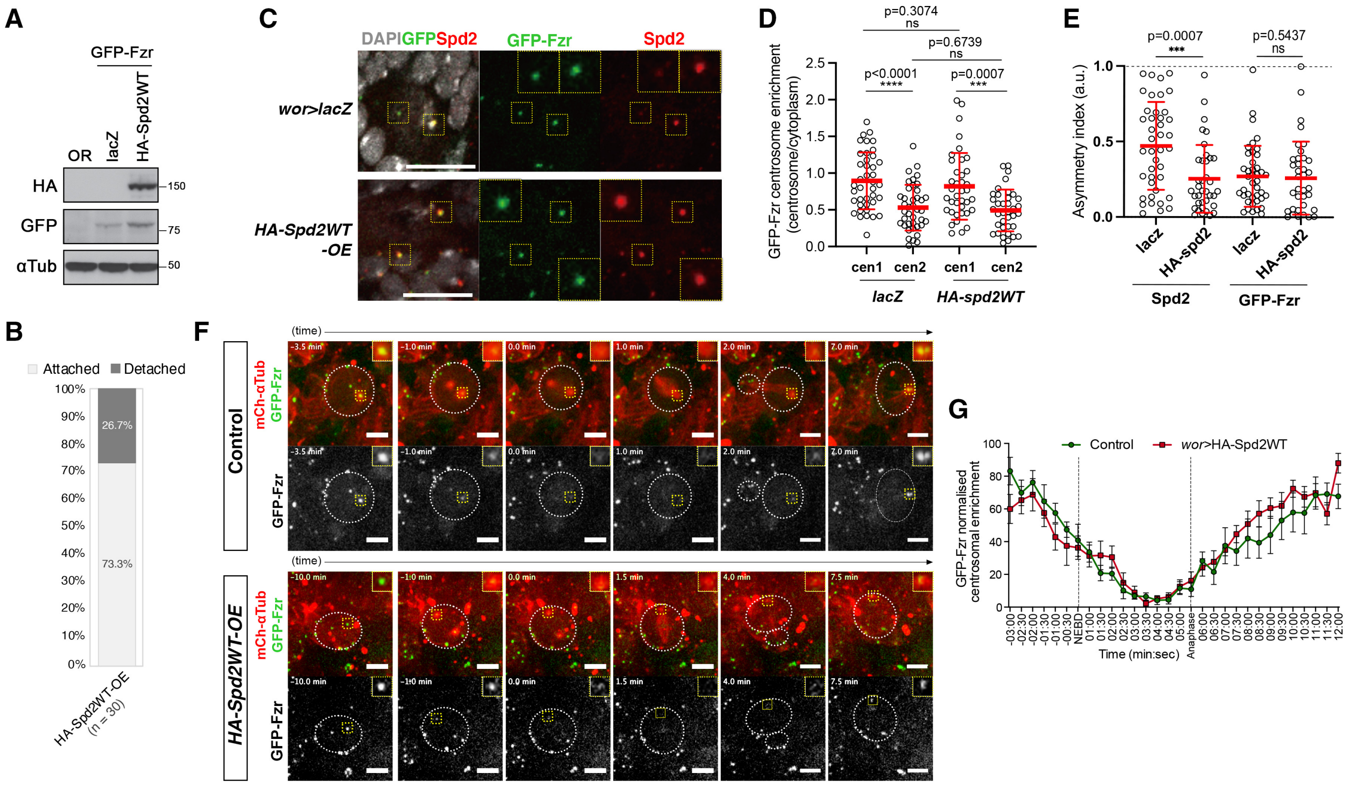

Figure EV5. The centrosomal localisation of Fzr is not affected by Spd2 accumulation.

A Western blot of the larval brain extracts from wild type (OR) or from the *pUbq-GFP-Fzr* line with NB-specific induction of lacZ (control) or HA-Spd2WT, by using HA, GFP and αTub antibodies. Expression of GFP-Fzr and HA-Spd2 was confirmed.

B In time lapse movies of third instar larval brains overexpressing HA-Spd2WT by *wor*-Gal4 (*HA-Spd2WT-OE*), the numbers of NBs that showed attached centrosomes and NBs with detached centrosomes during interphase were counted and their proportions are presented in bar graphs. *HA-Spd2WT-OE* NBs frequently showed apical centrosome detachment phenotypes. Thirty NBs (*n*) from 4 brains were analysed. *P*-values = 0.0003 (in comparison with control (Fig 2C), Pearson's chi-squared tests were performed).

C Representative images of interphase NBs in fixed control (*wor > lacZ*) and *HA-Spd2WT-OE* larval brains expressing GFP-Fzr under *pUbq* and stained for DAPI and Spd2. The dotted yellow squares indicate two centrosomes in the NBs. Scale bars: 10 μm.

D Centrosomal GFP-Fzr and Spd2 signals for each centrosome in individual interphase NBs were measured and relative signal intensities of each centrosome against cytoplasmic signals were presented in a scattered dot plots (see Materials and Methods). Among two centrosomes in each NB, the centrosome showing stronger signals was assigned as "cen1" and the other as "cen2". Centrosomal GFP-Fzr signals were not significantly affected by HA-Spd2WT overexpression. Red bars indicate means ± SD. Thirty-seven NBs (*n*) from four brains were analysed in each line.

E Asymmetric indexes of Spd2 and GFP-Fzr were calculated in control (*lacZ*) and *HA-Spd2WT-OE* NBs using the values in Fig EV5D. Asymmetric distributions of GFP-Fzr between the two centrosomes were not affected by HA-Spd2WT overexpression while those of Spd2 were affected. Red bars indicate means ± SD. Thirty-seven NBs (*n*) from four brains were analysed in each line.

F Selected images from time-lapse movies of larval NBs (in whole mount brain preparations) expressing *wor > mCh-αTub* (red) and *pUbq-GFP-Fzr* (green) alone (upper panels), or in combination with UAS-HA-Spd2WT (*HA-Spd2WT-OE*, lower panels), which were undergoing mitosis. Insets show higher magnification of the centrosomes (Movies EV11 and EV12). GFP-Fzr centrosomal dynamics were not affected by HA-Spd2WT over-expression. Scale bars correspond to 5 μm.

G Quantification of the relative centrosomal fluorescence intensity of GFP-Fzr during mitosis in control NBs and the *HA-Spd2WT-OE* NBs that showed the centrosome detachment phenotype. Eight NBs from at least three different larval brains were analysed and means ± SD are plotted in the line graph. GFP-Fzr dynamics during cell division was not affected by HA-Spd2WT overexpression.

Data information: In all statistical analyses in this figure, *P*-values were calculated using unpaired non-parametric Mann–Whitney U-tests.
Source data are available online for this figure.
