# Supplementary material for: APC/C‐dependent degradation of Spd2 regulates centrosome asymmetry in Drosophila neural stem cells
Source: EMBO Rep. 2023 Feb 28;24(4):e55607. doi: 10.15252/embr.202255607 (PMC10074082; doi:10.15252/embr.202255607)
Supplement: Supplementary file 2 — Movie EV1 [file EMBR-24-e55607-s011.zip › Movie EV1 legend.docx]

**Movie EV1 Example of a control GFP-Fzr NB showing the NB-specific centrosome behaviour**

A representative movie of a GFP-Fzr NB (control) undergoing asymmetric cell division. GFP-Fzr signals are shown in green and mCherry-Tubulin in red. During interphase, one of the two centrosomes was nucleating microtubules while the other was inactive. Upon mitotic entry both centrosomes increased microtubule nucleation (centrosome maturation), but the apical centrosome matured slightly earlier than the basal centrosome. After cell division, one centrosome remains active during interphase. Scale bar: 10 µm.
