# Supplementary material for: APC/C‐dependent degradation of Spd2 regulates centrosome asymmetry in Drosophila neural stem cells
Source: EMBO Rep. 2023 Feb 28;24(4):e55607. doi: 10.15252/embr.202255607 (PMC10074082; doi:10.15252/embr.202255607)
Supplement: Supplementary file 3 — Movie EV2 [file EMBR-24-e55607-s005.zip › Movie EV2 legend.docx]

**Movie EV2 Example of a Spd2WT-RES NB retaining an active centrosome during interphase**

A timelapse of a Spd2WT-RES NB undergoing two successive asymmetric divisions. RFP-Spd2 signals are shown in green and α-Tubulin-GFP in red. As wildtype NBs, this Spd2WT-RES NB retained microtubule nucleation activity at one centrosome during interphase and stably aligned mitotic spindle along the apico-basal axis with little deviations over the consecutive mitoses. Scale bar: 10 µm.
