# Supplementary material for: APC/C‐dependent degradation of Spd2 regulates centrosome asymmetry in Drosophila neural stem cells
Source: EMBO Rep. 2023 Feb 28;24(4):e55607. doi: 10.15252/embr.202255607 (PMC10074082; doi:10.15252/embr.202255607)
Supplement: Supplementary file 4 — Movie EV3 [file EMBR-24-e55607-s016.zip › Movie EV3 legend.docx]

**Movie EV3 Example of apical centrosome detachment in a Spd2DK-RES NB**

A movie of a Spd2DK-RES NB that exhibited apical centrosome detachment during interphase. Despite centrosome detachment this NB formed mitotic spindle at similar angles over the consecutive mitoses. RFP-Spd2 signals are shown in green and α-Tubulin-GFP in red. Scale bar: 10 µm.
