# Supplementary material for: APC/C‐dependent degradation of Spd2 regulates centrosome asymmetry in Drosophila neural stem cells
Source: EMBO Rep. 2023 Feb 28;24(4):e55607. doi: 10.15252/embr.202255607 (PMC10074082; doi:10.15252/embr.202255607)
Supplement: Supplementary file 5 — Movie EV4 [file EMBR-24-e55607-s018.zip › Movie EV4 legend.docx]

**Movie EV4 Example of apical centrosome detachment in a *fzr*RNAi NB**

A movie of a *fzrRNAi* NB that exhibited apical centrosome detachment during interphase. Despite centrosome detachment this NB formed mitotic spindle at similar angles over consecutive mitoses. Sas6-GFP signals are shown in green and mCherry-Tubulin in red. Scale bar: 10 µm.
