# Supplementary material for: APC/C‐dependent degradation of Spd2 regulates centrosome asymmetry in Drosophila neural stem cells
Source: EMBO Rep. 2023 Feb 28;24(4):e55607. doi: 10.15252/embr.202255607 (PMC10074082; doi:10.15252/embr.202255607)
Supplement: Supplementary file 6 — Movie EV5 [file EMBR-24-e55607-s007.zip › Movie EV5 legend.docx]

**Movie EV5 Example of apical centrosome detachment in a Spd2WT-OE NB**

A movie of a Spd2WT-OE NB that exhibited apical centrosome detachment during interphase. Despite the loss of an apically attached centrosome during interphase this NB could maintain the division axis over two consecutive mitoses. GFP-Spd2 signals are shown in green and mCherry-Tubulin in red. Scale bar: 10 µm
