# Supplementary material for: APC/C‐dependent degradation of Spd2 regulates centrosome asymmetry in Drosophila neural stem cells
Source: EMBO Rep. 2023 Feb 28;24(4):e55607. doi: 10.15252/embr.202255607 (PMC10074082; doi:10.15252/embr.202255607)
Supplement: Supplementary file 7 — Movie EV6 [file EMBR-24-e55607-s003.zip › Movie EV6 legend.docx]

**Movie EV6 Example of the bent spindle phenotype in a Spd2DK-OE NB**

A timelapse movie of a mitotic Spd2DK-OE NB that exhibited the bent spindle phenotype. GFP-Spd2 signals are shown in green and mCherry-Tubulin in red. Scale bar: 10 µm
