# Supplementary material for: APC/C‐dependent degradation of Spd2 regulates centrosome asymmetry in Drosophila neural stem cells
Source: EMBO Rep. 2023 Feb 28;24(4):e55607. doi: 10.15252/embr.202255607 (PMC10074082; doi:10.15252/embr.202255607)
Supplement: Supplementary file 8 — Movie EV7 [file EMBR-24-e55607-s009.zip › Movie EV7 legend.docx]

**Movie EV7 Example of a control Sas6-GFP NB that showed the division axis maintenance over three successive cell divisions**

A representative timelapse of a control Sas6-GFP NB undergoing three successive asymmetric divisions. Sas6-GFP signals are shown in green and mCherry-Tubulin in red. This control NB retained microtubule nucleation activity at one centrosome during interphase and stably maintained the division axis with little deviations over three consecutive mitoses. Scale bar: 10 µm.
