# Supplementary material for: APC/C‐dependent degradation of Spd2 regulates centrosome asymmetry in Drosophila neural stem cells
Source: EMBO Rep. 2023 Feb 28;24(4):e55607. doi: 10.15252/embr.202255607 (PMC10074082; doi:10.15252/embr.202255607)
Supplement: Supplementary file 9 — Movie EV8 [file EMBR-24-e55607-s008.zip › Movie EV8 legend.docx]

**Movie EV8 Example of division axis deviations and centriole missegregation in a Spd2DK-OE NB**

A timelapse movie of a Spd2DK-OE NB that exhibited severe division axis deviations over three successive mitoses. During the second and third mitoses, this NB missegregated the daughter centrosome (the centrosome that matured earlier than the other centrosome) into the GMCs. GFP-Spd2 signals are shown in green and mCherry-Tubulin in red. Scale bar: 10 µm
