# Supplementary material for: APC/C‐dependent degradation of Spd2 regulates centrosome asymmetry in Drosophila neural stem cells
Source: EMBO Rep. 2023 Feb 28;24(4):e55607. doi: 10.15252/embr.202255607 (PMC10074082; doi:10.15252/embr.202255607)
Supplement: Supplementary file 11 — Movie EV10 [file EMBR-24-e55607-s006.zip › Movie EV10 legend.docx]

**Movie EV10 Example of centriole missegregation in a Spd2DK-OE NB**

A timelapse movie of a Spd2DK-OE NB that showed missegregation of the daughter centrosome. In this NB, the daughter centrosome (which matured earlier than the other centrosome) was segregated into the GMCs. GFP-Spd2 signals are shown in green and mCherry-Tubulin in red. Scale bar: 10 µm.
