# Supplementary material for: APC/C‐dependent degradation of Spd2 regulates centrosome asymmetry in Drosophila neural stem cells
Source: EMBO Rep. 2023 Feb 28;24(4):e55607. doi: 10.15252/embr.202255607 (PMC10074082; doi:10.15252/embr.202255607)
Supplement: Supplementary file 12 — Movie EV11 [file EMBR-24-e55607-s017.zip › Movie EV11 legend.docx]

**Movie EV11 Example of GFP-Fzr dynamics in a control wor>lacZ NB**

A timelapse movie of a wor>lacZ NB expressing GFP-Fzr. GFP-Fzr signals are shown in green and mCherry-Tubulin in red. Scale bar: 10 µm
