# Supplementary material for: APC/C‐dependent degradation of Spd2 regulates centrosome asymmetry in Drosophila neural stem cells
Source: EMBO Rep. 2023 Feb 28;24(4):e55607. doi: 10.15252/embr.202255607 (PMC10074082; doi:10.15252/embr.202255607)
Supplement: Supplementary file 13 — Movie EV12 [file EMBR-24-e55607-s002.zip › Movie EV12 legend.docx]

**Movie EV12 Example of GFP-Fzr dynamics in a HA-Spd2WT-OE NB**

A timelapse movie of a HA-Spd2WT-OE NB expressing GFP-Fzr. The centrosome localisation of GFP-Fzr in HA-Spd2WT-OE NBs was comparable to that in control wor>lacZ NBs. GFP-Fzr signals are shown in green and mCherry-Tubulin in red. Scale bar: 10 µm
