# Supplementary material for: APC/C‐dependent degradation of Spd2 regulates centrosome asymmetry in Drosophila neural stem cells
Source: EMBO Rep. 2023 Feb 28;24(4):e55607. doi: 10.15252/embr.202255607 (PMC10074082; doi:10.15252/embr.202255607)
Supplement: Supplementary file 14 — Movie EV13 [file EMBR-24-e55607-s012.zip › Movie EV13 legend.docx]

**Movie EV13 Example of Spd2 FRAP analysis in an interphase Spd2WT-RES NB**

A representative timelapse movie of the FRAP analysis of centrosomal Spd2 signals in an interphase Spd2WT-RES NB. REP-Spd2 signals are shown in green.
