# Supplementary material for: APC/C‐dependent degradation of Spd2 regulates centrosome asymmetry in Drosophila neural stem cells
Source: EMBO Rep. 2023 Feb 28;24(4):e55607. doi: 10.15252/embr.202255607 (PMC10074082; doi:10.15252/embr.202255607)
Supplement: Supplementary file 15 — Movie EV14 [file EMBR-24-e55607-s015.zip › Movie EV14 legend.docx]

**Movie EV14 Example of Spd2 FRAP analysis in an interphase Spd2DK-OE NB**

A representative timelapse movie of the FRAP analysis of centrosomal Spd2 signals in an interphase Spd2DK-OE NB. GFP-Spd2 signals are shown in green.
