# Supplementary figures and images for: APC/C‐dependent degradation of Spd2 regulates centrosome asymmetry in Drosophila neural stem cells
Source: EMBO Rep. 2023 Feb 28;24(4):e55607. doi: 10.15252/embr.202255607 (PMC10074082; doi:10.15252/embr.202255607)

# FigEV5a

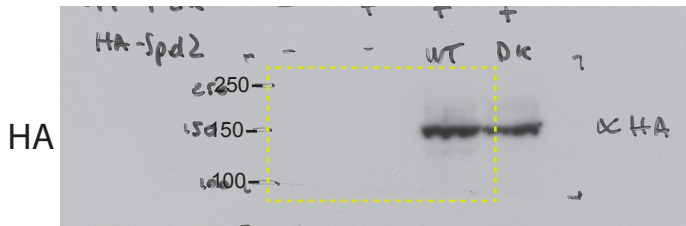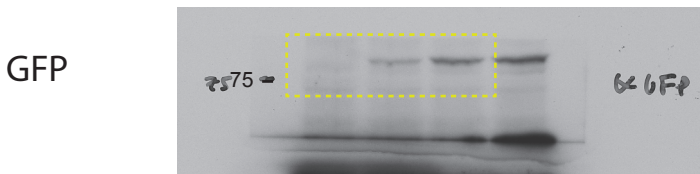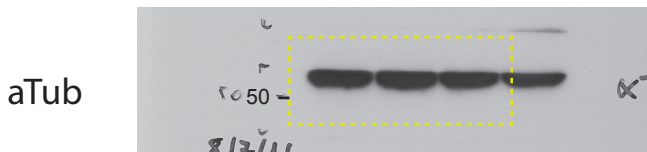

Supplement: Supplementary file 16 — Source Data for Expanded View [file EMBR-24-e55607-s019.zip › EV_Figure_Source_Data/EMBOR-2022-55607V3-Figure_EV5_WB_source_data-sd.pdf]

**FigEV2a**

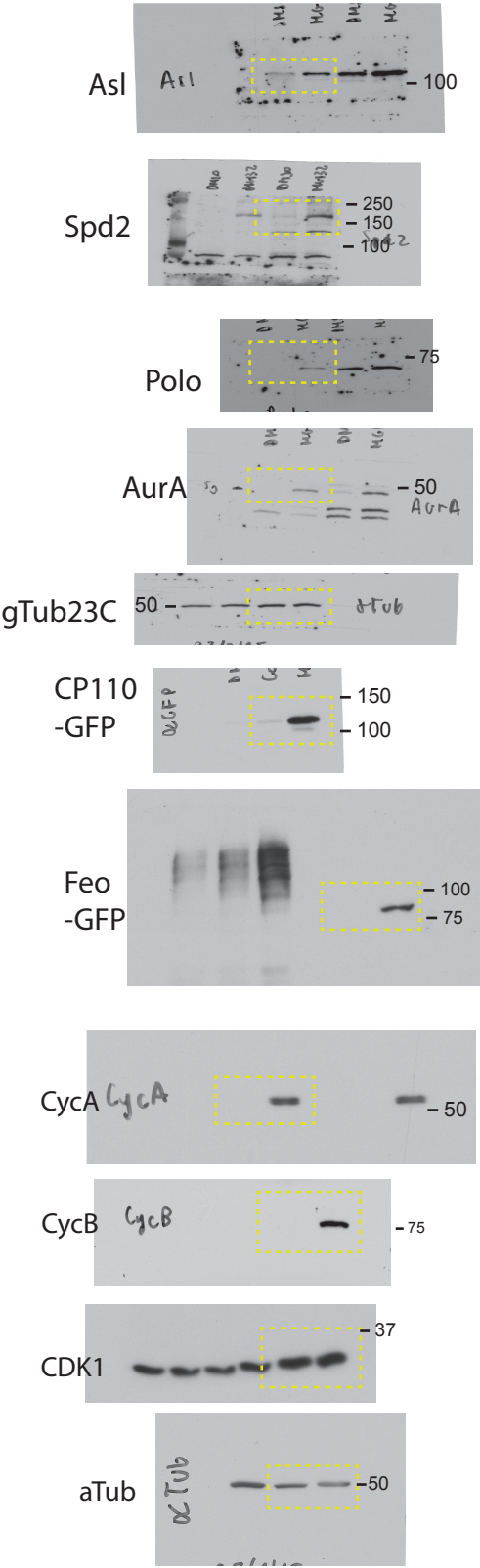

**FigEV2b**

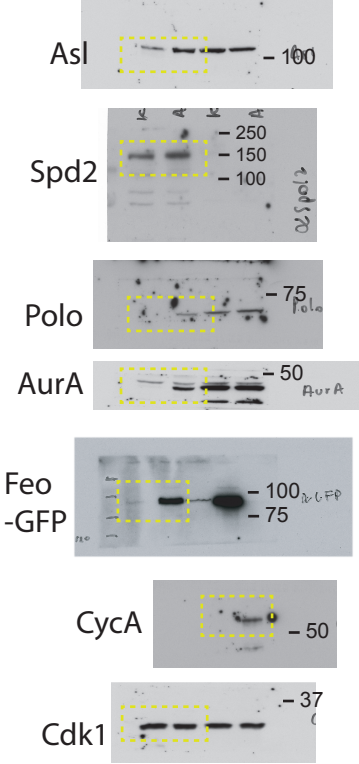

**FigEV2c**

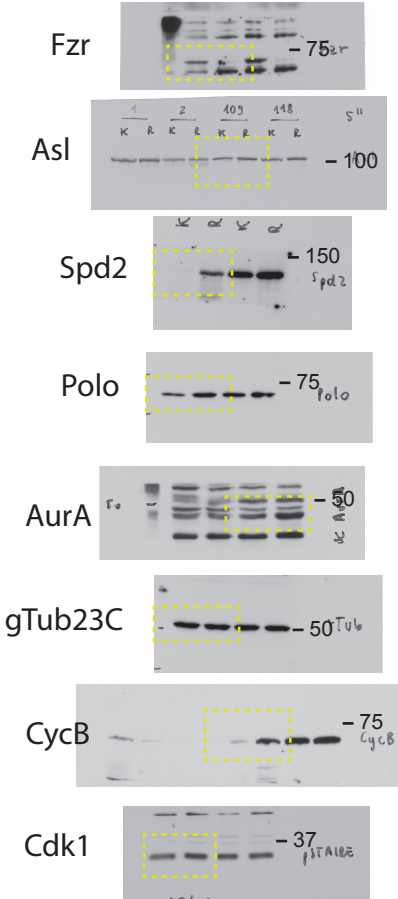

**FigEV2g**

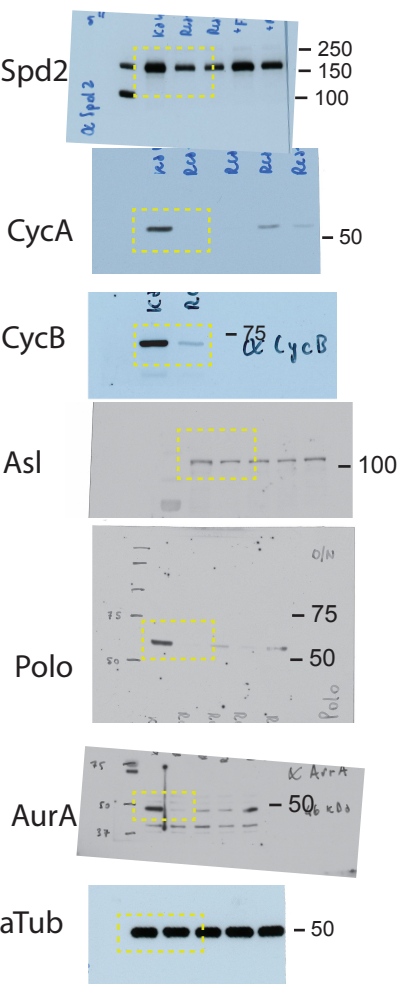

**FigEV2d**

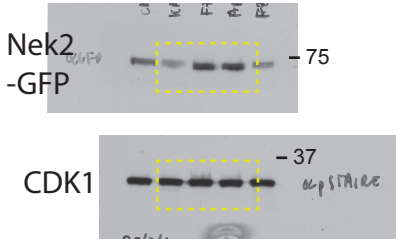

**FigEV2e**

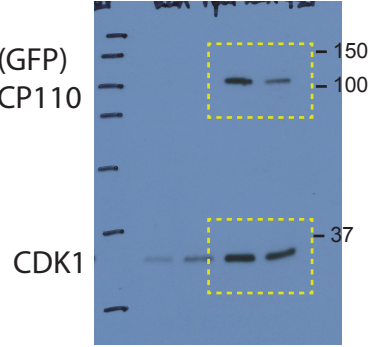

**FigEV2H**

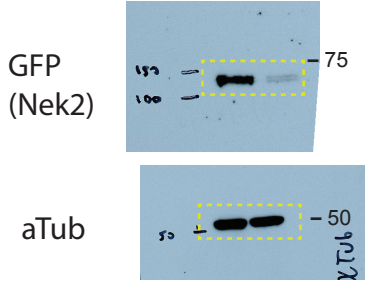

Supplement: Supplementary file 16 — Source Data for Expanded View [file EMBR-24-e55607-s019.zip › EV_Figure_Source_Data/EMBOR-2022-55607V3-Figure_EV2_WB_source_data-sd.pdf]

**Fig1B**

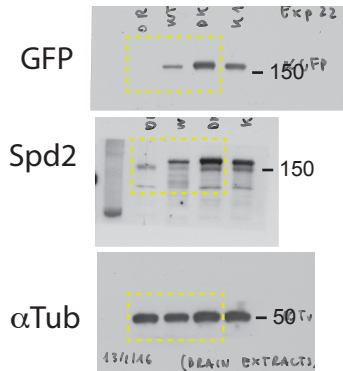

**Fig1C**

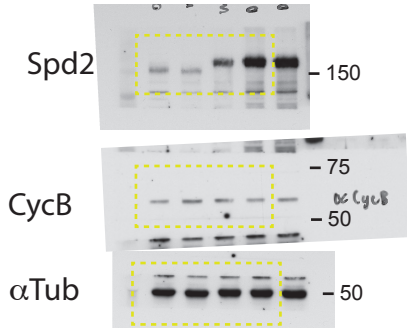

Supplement: Supplementary file 18 — Source Data for Figure 1 [file EMBR-24-e55607-s013.pdf]
