# Supplementary material for: APC/C‐dependent degradation of Spd2 regulates centrosome asymmetry in Drosophila neural stem cells
Source: EMBO Rep. 2023 Feb 28;24(4):e55607. doi: 10.15252/embr.202255607 (PMC10074082; doi:10.15252/embr.202255607)
Supplement: Supplementary file 17 — PDF+ [file EMBR-24-e55607-s004.pdf]

Article

SOURCE DATA TRANSPARENT PROCESS OPEN ACCESS

EMBO reports

APC/C-dependent degradation of Spd2 regulates centrosome asymmetry in *Drosophila* neural stem cells

Francesco Meghini[1,†], Torcato Martins[1,2,†], Qian Zhang[3,†], Nicolas Loyer[4], Michelle Trickey[5], Yusanjiang Abula[3], Hiroyuki Yamano[5], Jens Januschke[4,*] & Yuu Kimata[1,3,**]

Abstract

A functional centrosome is vital for the development and physiology of animals. Among numerous regulatory mechanisms of the centrosome, ubiquitin-mediated proteolysis is known to be critical for the precise regulation of centriole duplication. However, its significance beyond centrosome copy number control remains unclear. Using an *in vitro* screen for centrosomal substrates of the APC/C ubiquitin ligase in *Drosophila*, we identify several conserved pericentriolar material (PCM) components, including the inner PCM protein Spd2. We show that Spd2 levels are controlled by the interphase-specific form of APC/C, APC/C^{Fzr}, in cultured cells and developing brains. Increased Spd2 levels compromise neural stem cell–specific asymmetric PCM recruitment and microtubule nucleation at interphase centrosomes, resulting in partial randomisation of the division axis and segregation patterns of the daughter centrosome in the following mitosis. We further provide evidence that APC/C^{Fzr}-dependent Spd2 degradation restricts the amount and mobility of Spd2 at the daughter centrosome, thereby facilitating the accumulation of Polo-dependent Spd2 phosphorylation for PCM recruitment. Our study underpins the critical role of cell cycle–dependent proteolytic regulation of the PCM in stem cells.

Keywords asymmetric cell division; cell cycle; centrosome; stem cell; ubiquitination
Subject Categories Cell Cycle; Post-translational Modifications & Proteolysis; Stem Cells & Regenerative Medicine
DOI 10.15252/embr.202255607 | Received 17 June 2022 | Revised 31 December 2022 | Accepted 11 January 2023 | Published online 28 February 2023
EMBO Reports (2023) 24: e55607

Introduction

The centrosome is a non-membrane-bound organelle that acts as a major microtubule-organising centre in animal cells, orchestrating cytoskeletal network in interphase and promoting bipolar spindle assembly in mitosis (Bornens, 2021; Hoffmann, 2021). Each centrosome contains a pair of rod-shaped structures, centrioles, at its core and the surrounding proteinous matrix, pericentriolar material (PCM; Woodruff *et al*, 2014; Conduit *et al*, 2015; Fu *et al*, 2015; Bornens, 2021). PCM contains microtubule polymerisation factors such as γ-Tubulin (γ -Tub) and is responsible for the microtubule nucleation activity of the centrosome. Prior to mitosis, PCM rapidly increases its size and microtubule organising capacity (a process known as centrosome maturation), preparing for spindle assembly. Several evolutionally conserved proteins are known to be required for this process, which include Polo kinase (Polo-like kinase 1, Plk1 in vertebrates) and PCM components Spd2 (Cep192 in vertebrates) and Centrosomin (Cnn, CDK5RAP2 in vertebrates; Sunkel & Glover, 1988; Golsteyn *et al*, 1995; Lane & Nigg, 1996; Megraw *et al*, 1999; Pelletier *et al*, 2004; Dix & Raff, 2007; Gomez-Ferreria *et al*, 2007; Giansanti *et al*, 2008; Zhu *et al*, 2008b; Lizarraga *et al*, 2010; Chinen *et al*, 2021). In the current model for the *Drosophila* mitotic centrosome assembly, Spd2 and Cnn cooperate to form a mesh-like structure around centrioles that serves as a scaffold for other mitotic PCM components; Polo-dependent phosphorylation of Spd2 and Cnn stimulates the assembly of this PCM scaffold (Conduit et al, 2014a, 2015; Feng *et al*, 2017; Alvarez-Rodrigo *et al*, 2019). Although some details may differ, elements of this model are likely evolutionally conserved (Woodruff *et al*, 2017; Lee *et al*, 2021).

In multicellular organisms including humans, the centrosome plays a critical role in the regulation of development and tissue homeostasis, often through its cell type–specific functions (Tang & Marshall, 2012; Araújo, 2019; Bornens, 2021; Qi & Zhou, 2021). As a prime example, in the *Drosophila* larval neural stem cell or

1 Department of Genetics, University of Cambridge, Cambridge, UK
2 Department of Medical Sciences, Institute of Biomedicine-iBiMED, University of Aveiro, Aveiro, Portugal
3 School of Life Science and Technology, ShanghaiTech University, Shanghai, China
4 School of Life Science, University of Dundee, Dundee, UK
5 UCL Cancer Institute, University College London, London, UK
*Corresponding author. Tel: +44 01382 386169; E-mail: j.januschke@dundee.ac.uk
**Corresponding author. Tel: +86 021 20684508; E-mail: ykimata@shanghaitech.edu.cn
†These authors contributed equally to this work

© 2023 The Authors. Published under the terms of the CC BY 4.0 license.

neuroblast (NB), which undergoes stereotypical asymmetric cell division (Gallaud *et al*, 2017), the centrosome regulates spindle orientation and division axis maintenance through a unconventional behaviour specific to this cell type (Rebollo *et al*, 2007; Rusan & Peifer, 2007). Unlike most somatic cells, in which centrosomes lose microtubule nucleation activity during interphase, the NB retains microtubule nucleation capacity at one of the two centrosomes (Rebollo *et al*, 2007; Rusan & Peifer, 2007; Januschke & Gonzalez, 2010). The active centrosome is attached to the apical cortex via an astral microtubule network, which allows the NB to form mitotic spindle along the apicobasal axis upon mitotic entry, by fixing one pole. Through this mechanism, the NB can maintain the division axis over consecutive divisions, delivering its differentiating daughters (ganglion mother cells, GMCs) to close basal positions (Rebollo *et al*, 2007; Rusan & Peifer, 2007; Januschke & Gonzalez, 2010). Several evolutionally conserved centrosome components and regulators, including Polo, Plk4, Centrobin, CEP135, DPLP (Pericentrin in mammals) and Wdr62, have been identified to regulate this NB-specific asymmetric centrosome behaviour (Januschke *et al*, 2013; Lerit & Rusan, 2013; Singh *et al*, 2014; Conduit *et al*, 2014a; Ramdas Nair *et al*, 2016; Gambarotto *et al*, 2019). However, how the activities of these regulators are spatiotemporally coordinated to regulate the differential centrosome behaviour remains largely elusive.

The behaviour and activity of the centrosome are tightly coordinated with the cell cycle. Various posttranslational modifications on centrosome components are implicated in this coordination of the centrosome cycle with the cell cycle (Nigg & Stearns, 2011; Blanco-Ameijeiras *et al*, 2022). Of these, ubiquitination, followed by proteasomal degradation of ubiquitinated proteins, is considered key to the regulation of centriole duplication (Darling *et al*, 2017; Badarudeen *et al*, 2021). However, the importance of ubiquitination-dependent proteolysis beyond centrosome number control remains to be established.

The APC/C (anaphase-promoting complex/cyclosome) is a ubiquitin ligase complex that plays a central role in cell cycle control (Pines, 2011; Chang & Barford, 2014). The APC/C associates with one of two co-activators, Fizzy (Fzy) and Fizzy-related (Fzr) (also called CDC20 and CDH1 or Fzr1, respectively). APC/C^{Fzy} is activated at anaphase to trigger chromosome segregation and mitotic exit, while APC/C^{Fzr} is activated upon mitotic exit and remains active during interphase (Pines, 2011; Chang & Barford, 2014). It has been shown that APC/C^{Fzr} controls centrosome duplication by ubiquitinating various centriolar proteins, such as SAS6 and STIL (Strnad *et al*, 2007; Arquint & Nigg, 2014). APC/C^{Fzr} also targets major regulators of PCM and centrosome maturation, Plk1 and Aurora A (AurA) for degradation (Lindon & Pines, 2004; Floyd *et al*, 2008). However, the role of APC/C-dependent proteolysis in cell type–specific functions of the centrosome remains unclear.

To obtain a comprehensive understanding of the role of APC/C-dependent proteolysis in centrosome regulation, we conducted a biochemical screen of known components and regulators of the *Drosophila* centrosome and identified several conserved PCM proteins, including Spd2, as putative centrosomal APC/C substrates. Spd2 is an inner PCM protein and is essential for recruiting other PCM proteins and microtubule nucleation activity of the mitotic centrosome (Pelletier *et al*, 2004; Dix & Raff, 2007; Gomez-Ferreria *et al*, 2007; Giansanti *et al*, 2008; Zhu *et al*, 2008a, 2008b). We investigated the *in vivo* function of APC/C-dependent degradation of Spd2 and found that the cellular levels of Spd2 are critical for the regulation of the interphase centrosome in the *Drosophila* neural stem cell. This study provides the first demonstration for a role of ubiquitin-mediated degradation of a conserved PCM protein for cell-type-specific functions of the centrosome, in particular, in asymmetrical cell division of stem cells.

Results

In vitro screen for centrosomal targets of APC/C-dependent proteolysis

To investigate the role of the APC/C-dependent protein degradation in centrosome regulation, we conducted a biochemical screen for centrosomal APC/C substrates in *Drosophila*. We adopted a cell-free degradation assay using *Xenopus laevis* egg extracts in which APC/C-dependent proteolysis can be reconstituted *in vitro* (Yamano *et al*, 2009). *Drosophila* orthologues of mitotic cyclins, well-established canonical APC/C substrates (King *et al*, 1995; Sudakin *et al*, 1995), were degraded upon APC/C activation in this assay (Fig EV1A). We screened 55 *Drosophila* centrosomal proteins, including 51 proteins that were previously identified in RNAi and proteomic screens as critical regulators of the centrosome (Andersen *et al*, 2003; Dobbelaere *et al*, 2008; Müller *et al*, 2010; Dobbelaere, 2015), as well as Sgt1, γTub37C, Pen and Cnot1, which we previously identified as putative *in vivo* APC/C-interacting proteins (Martins *et al*, 2009; Haider *et al*, 2015; Meghini *et al*, 2016; Fig EV1B). Ana1, Ana3, Plk4, Rcd1 and Rcd2 showed APC/C-dependent degradation in mitotic extracts while Asterless (Asl, CEP152 in vertebrates), AurA, CP110, Feo, Klp61F, Nek2, Polo, Rcd4 and Spd2 showed degradation in interphase extracts (Fig EV1C and D). The degradation of all the five mitotic substrates could be only partially inhibited by addition of an APC/C inhibitor *S. pombe* Mes1 (Kimata *et al*, 2008), indicating a certain degree of intrinsic instability of these proteins in this assay (Fig EV1C). Meanwhile, among the 9 interphase substrates, all but Polo showed a clear dependency on APC/C^{Fzr} activity (Fig EV1D). Nek2 and AurA showed rapid and nearly complete degradation, whereas the rest, including Spd2, showed slow and/or incomplete degradation (Fig EV1D).

We validated some of these candidates in cultured *D.mel-2* cells. Inhibition of mitotic APC^{Fzy} activity induces extensive apoptosis. We therefore focused our analysis on the candidates of APC/C^{Fzr} substrates. Similar to the control CycA and CycB, the cellular levels of endogenous Spd2, Polo, AurA and Asl proteins, as well as GFP-CP110 and Feo-GFP, expressed by transient transfection, were increased upon addition of the proteasome inhibitor MG-132, while the levels of γTubulin (γTub23C) was unaffected (Fig EV2A). In alignment with this result, RNAi against Fzr or APC/C core subunit Apc4 caused accumulation of Spd2, Polo, AurA, Asl and Nek2-GFP (Fig EV2B–E). We also induced hyper-activation of APC/C^{Fzr} by depleting endogenous Rca1 (Emi1 in vertebrates), an APC/C^{Fzr} inhibitor (Grosskortenhaus & Sprenger, 2002), by RNAi and found that the levels of Spd2, Polo, AurA, Asl and Nek2-GFP were decreased upon Rca1 depletion (Fig EV2F and G). Together, these results suggest that Spd2, AurA, Polo, Asl and Nek2 are centrosomal targets of APC/C^{Fzr}-dependent proteolysis in *Drosophila*.

 © 2023 The Authors

Spd2 protein levels are controlled by APC/C^{Fzr}-dependent degradation *in vivo*

We previously found that Spd2 acts as a centrosomal loading factor for Fzr, by directly binding it and recruiting it to the interphase centrosome. Spd2 binds the WD40 repeat domain of Fzr via D-box and KEN-box sequences (Davey & Morgan, 2016) and is also ubiquitinated and degraded by APC/C^{Fzr} *in vitro*, in a manner similar to canonical APC/C substrates (Meghini *et al*, 2016). However, a potential function of APC/C-dependent Spd2 ubiquitination/degradation *in vivo* has not been determined yet.

To investigate the role of APC/C-dependent degradation of Spd2, we examined the effect of Spd2 stabilisation/degradation on the centrosome using the larval NB as a model. To this end, we first created a series of *Drosophila* lines in which increasing levels of Spd2 proteins are expressed in NBs (Fig 1A, also see Materials and Methods). We generated Spd2 overexpression lines (*Spd2WT-OE* and *Spd2DK-OE*), in which GFP-tagged wild-type Spd2 (Spd2WT) or a stable mutant form of Spd2 (Spd2DK), which is not recognised by APC/C^{Fzr} due to mutations in all its degrons, four D-boxes and one KEN-box (Meghini *et al*, 2016), is overexpressed in a wild-type background by an NB-specific Gal4 driver, *worniu*-Gal4 (*wor*-Gal4; Zhu *et al*, 2008a), using the Gal4/UAS system (Brand & Perrimon, 1993; Fig 1A). We also complemented the *spd2* null mutant (*spd2$^{z3-5711}$/Df(3L)BSC561*) with RFP-tagged Spd2WT or Spd2DK mildly expressed under a constitutive polyubiquitin gene promoter (*pUbq*; Lee *et al*, 1988; hence, called "rescue", Fig 1A). In addition, we generated an Fzr knockdown line (*fzrRNAi*) in which endogenous Fzr is depleted in NBs by inducing *fzrRNAi* by *wor*-Gal4 (Fig 1A).

Immunoblot analyses using the larval brain extracts from *Spd2WT-OE* and *Spd2DK-OE* lines showed that, despite being expressed under the same transcriptional control, GFP-Spd2DK proteins showed higher accumulation than GFP-Spd2WT proteins (2.07-fold difference in the mean normalised band intensities, Fig 1B). Similarly, higher accumulation of RFP-Spd2DK proteins, compared with RFP-Spd2WT proteins, were observed in brain extracts from *Spd2WT-RES* and *Spd2DK-RES* lines (2.08-fold difference, Fig 1C), confirming that the D-Boxes and KEN-box regulate Spd2 protein levels posttranslationally *in vivo*. In this analysis, we could not observe any significant change in endogenous Spd2 levels in *fzrRNAi* (Fig 1C); this is perhaps expected as *worniu* is expressed only in a small fraction of cells (i.e. NBs) in the larval brain.

We also analysed Spd2 protein levels in larval NBs in different conditions quantifying fluorescence intensities using an Spd2 antibody. We measured the local signal intensities of Spd2 immunofluorescence at the centrosomes and in the cytoplasm (Fig 1D, see Materials and Methodsfor more details). *Spd2WT-RES* NBs did not show any significant increase in Spd2 levels when compared with control both at the centrosomes and in the cytoplasm (Fig 1D). *fzrRNAi* NBs showed a subtle but statistically significant increase in Spd2 levels at the centrosome, compared with control (1.29 ± 0.50-fold increase in the normalised mean signal intensities, $P = 0.0078$, Student *t*-test, Fig 1D). *Spd2WT-OE* NBs showed substantial increases both in the centrosomal and cytoplasmic Spd2 levels, compared with control (1.93 ± 1.08-fold and 3.62 ± 1.46-fold, respectively. Fig. 1D). These data indicate that Spd2 levels in wild-type and *Spd2WT-RES* larval NBs are comparable, while Spd2 levels are slightly increased in *fzrRNAi* NBs and progressively increased in *Spd2WT-OE* and *Spd2DK-OE* NBs, supporting the interpretation that Spd2 protein levels are regulated by APC/C^{Fzr}-dependent degradation *in vivo*, at least in larval NBs.

Spd2 accumulation compromises the stem cell–specific control of interphase centrosome activity

To monitor the dynamic behaviour and microtubule nucleation activity of the centrosome, we adapted an *ex vivo* whole-mount brain culture technique to observe the asymmetric cell divisions of NBs (Pampalona *et al*, 2015; Januschke & Loyer, 2020). We confirmed that expression of RFP-tagged Spd2WT or Spd2DK in a *spd2* null background (i.e., *Spd2WT/DK-RES*) can restore PCM accumulation and microtubule nucleation at mitotic centrosomes as previously shown (Meghini *et al*, 2016). Indeed, in *Spd2WT-RES* and *Spd2DK-RES* larval brains, most NBs formed mitotic spindles with two clearly focused poles and underwent proper chromosome segregation and asymmetric cell division, similar to NBs in control brains (Movies EV1–EV3). This was also true for most NBs analysed in *fzrRNAi*, *Spd2WT-OE* and *Spd2DK-OE* brains (Movies EV4–EV6). A small fraction of NBs of these genotypes transiently exhibited abnormal spindle morphologies, such as multipolar, monopolar spindle or "bent" spindles (Fig EV3A and B; Movie EV6). High levels of Spd2 proteins did not seem to be accountable for these defects, as we did not observe such abnormalities in *Spd2WT-OE* NBs overexpressing GFP-Spd2WT (0.0%, $n = 17$, Fig EV3B; Movie EV5). The large majority of NBs from all lines, however, formed bipolar spindle with asters (Fig EV3A and B; Movies EV1–EV6). These data suggest that the mitotic function of the centrosome, i.e. centrosome maturation and microtubule nucleation at spindle poles, is robust to increased Spd2 levels, which is in line with our results that Spd2 did not appear to be a target of APC/C *in vitro* in mitotic extracts (Fig EV1; Meghini *et al*, 2016).

We next examined the effect of Spd2 accumulation on the interphase centrosome. Larval NB centrosomes exhibit an unconventional behaviour during interphase (Rebollo *et al*, 2007; Rusan & Peifer, 2007). The daughter centrosome retains PCM and microtubule nucleation activity after mitotic exit and is tethered to the apical cell cortex through astral microtubules throughout interphase (Fig 2A; Rebollo *et al*, 2007; Rusan & Peifer, 2007; Conduit & Raff, 2010; Januschke *et al*, 2011). As we found that Spd2 was a target of the interphase-specific APC/C^{Fzr}, but not of the mitotic APC/C^{Fzy}, we speculated that APC/C^{Fzr}-dependent degradation of Spd2 may play a role in regulating interphase centrosomes in NBs.

Consistent with previous reports, in all the NBs examined in control (*pUbq-Sas6-GFP* or *pUbq-GFP-Fzr*, expressing Sas6-GFP or GFP-Fzr as a centrosome marker) and *Spd2WT-RES* brains ($n = 29$ or 45, respectively), one centrosome kept nucleating microtubules during interphase and was stably anchored at the proximity of the apical cell cortex (Fig 2C; Movies EV1, EV2, and EV7). However, in significant fractions of NBs in the lines with increased Spd2 levels (*fzrRNAi*, *Spd2DK-RES* and *Spd2WT/DK-OE*), both centrosomes lost microtubule nucleation capacity during interphase, and the apical centrosome became detached from the cortex to start wandering around the cytoplasm (Fig 2A–C; Movies EV3–EV5). Intriguingly, the frequencies of this phenotype ("apical centrosome detachment")

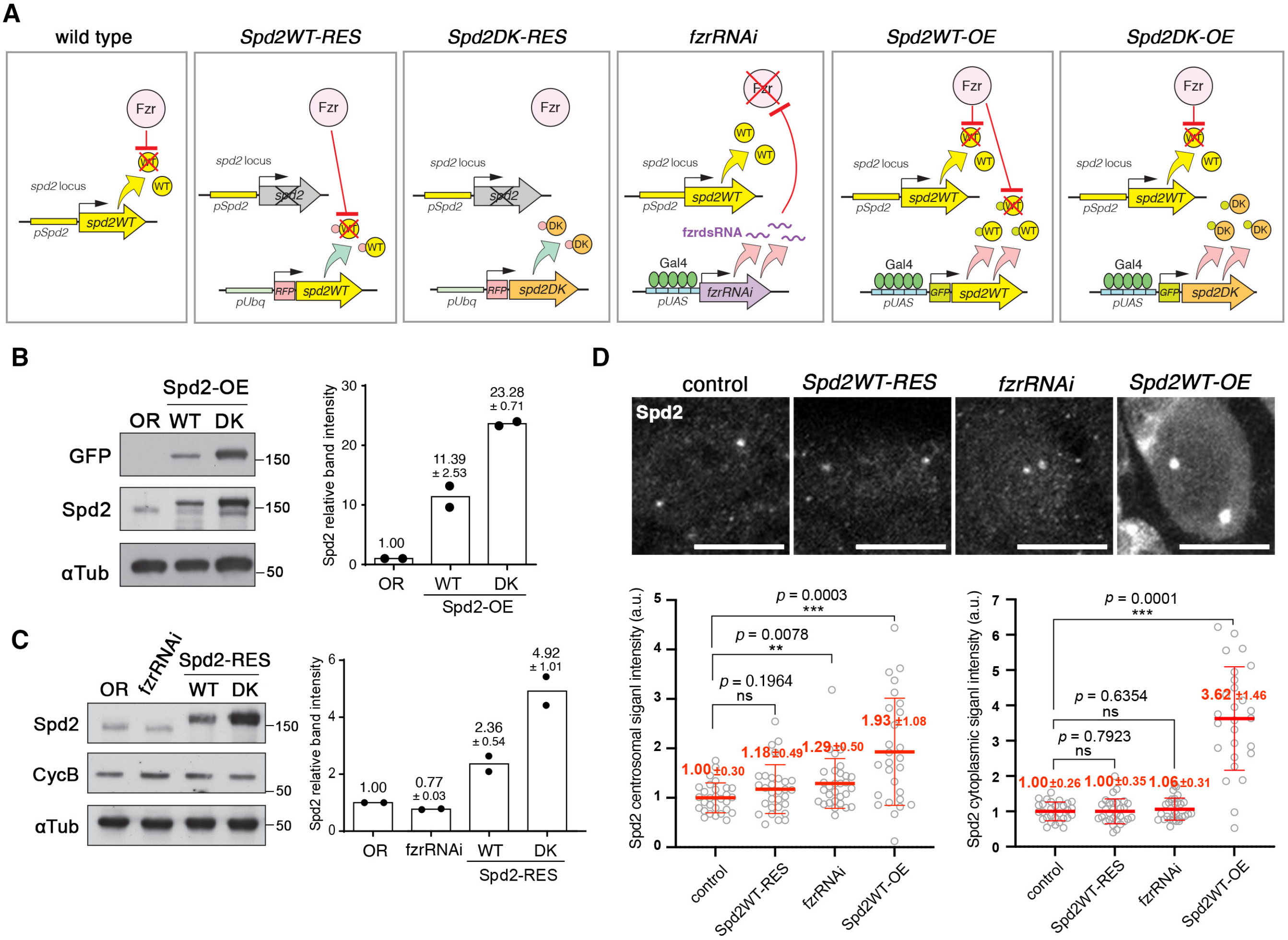

Figure 1. The cellular levels of the conserved PCM protein Spd2 are regulated by APC/C-dependent proteolysis *in vivo*.

A A schematic diagram showing the genetic compositions in the *Drosophila* lines that express different levels of Spd2 proteins in NBs used in this study. *pSpd2* and *pUAS* indicate the native *spd2* promoter and UAS sequences, respectively. Gal4 is expressed specifically in NBs by the control of a NB-specific *worniu* gene promoter.

B The Western blot analysis of GFP, Spd2 and αTub (loading control) in the brain extracts from wild-type (*Oregon R*, *OR*), *Spd2WT-OE* and *Spd2DK-OE* third instar larva. Representative immune blot images are shown on the left. The signal intensities of Spd2 bands were quantified and normalised against αTub values. The values relative to control of each biological replicate ($n = 2$), and their means are shown as dots and bars, respectively, in a scatted dot plot on the right. The numbers above the bars indicate means ± Range. GFP-Spd2DK proteins in *Spd2DK-OE* larval brain extracts showed approximately 2.04-fold higher accumulation than GFP-Spd2WT proteins in *Spd2WT-OE* brain extracts.

C The Western blot analysis of Spd2, CycB and αTub in OR, *fzrRNAi*, *Spd2WT-RES* and *Spd2DK-RES* brain extracts. The signal intensities of Spd2 bands were quantified and normalised against αTub values. The values relative to control, and their means are presented in a scatted dot plot on the right (biological replicate $n = 2$). Means ± Range are shown above the bars. RFP-Spd2DK proteins in *Spd2DK-RES* larval brain extracts showed approximately 2.08-fold higher accumulation than RFP-Spd2WT proteins in *Spd2WT-RES* brain extracts.

D Larval brains of OR, *Spd2WT-RES*, *fzrRNAi* and *Spd2WT-OE* flies were stained by an anti-Spd2 antibody. Representative images of NBs are shown on the top. Scale bar: 10 μm. The local signal intensities of Spd2 immunofluorescence at the centrosomes and in the cytoplasm of individual NBs in each brain were measured, and the normalised Spd2 signal intensities in individual NBs were presented as scattered dot plots (see Materials and Methods for details). The lines indicate means ± SD. The numbers of NBs (n) and the numbers of brains analysed (N) are control: $n = 29$ ($N = 6$), *Spd2WT-RES*: $n = 30$ ($N = 4$), *fzrRNA*: $n = 30$ ($N = 6$), and *Spd2WT-OE*: $n = 27$ ($N = 10$). P values were calculated using non-parametric two-tailed Mann–Whitney tests. Based on these results, the relative centrosomal Spd2 protein levels in NBs are estimated as wild-type (or control): *Spd2WT-RES*: *fzrRNAi*: *Spd2DK-RES*: *Spd2WT-OE*: *Spd2DK-OE* = 1: 1.00–1.18: 1.06–1.29: 2.08–2.45: 1.93–3.62: 3.94–7.38 (see Materials and Methods for details).

Source data are available online for this figure.

increased with increasing Spd2 levels observed in these conditions (Fig 1 and 2C): approximately 21.3% ($n = 80$) of NBs in *fzr*RNAi or *Spd2DK-RES* larval brains showed apical centrosome detachment in interphase which rose to 65.1% ($n = 43$) in *Spd2WT-OE* brains and even 90.0% in *Spd2DK-OE* ($n = 40$, Fig 2C). This observation shows that the microtubule nucleation ability and the anchoring of the daughter centriole to the apical cortex are sensitive to an increase in Spd2 levels and may suggests that the APC/C-dependent

 © 2023 The Authors

degradation of Spd2 is critical for regulating asymmetric centrosomes behaviour in larval NBs.

It was previously shown that various PCM proteins are differentially recruited to the two centrosomes in the NB, regulating their behaviour (Lerit & Rusan, 2013). We therefore examined the effect of altered Spd2 levels on the recruitment of major PCM proteins to centrosomes in interphase NBs. To this end, we measured the fluorescent signal intensities of the PCM proteins at the two centrosomes in individual NBs and determined the degree of their asymmetric distribution, as described previously (Lerit & Rusan, 2013; Fig 2D and E, see Materials and Methods for details). In interphase NBs in control and *Spd2WT-RES* brains, the PCM proteins Spd2, Cnn, AurA and γ-Tubulin (γ-Tub) showed an asymmetric distribution, being enriched on one centrosome, while the pan-centriolar component Asl (Fu & Glover, 2012; Mennella *et al*, 2012) was symmetrically distributed (Fig 2D and E). In contrast, in *Spd2DK-RES* NBs, Spd2 and γ-Tub showed significantly reduced asymmetric localisation patterns at the two centrosomes and become more symmetrically distributed, while Cnn appears to be unaffected (Fig 2D and E).

Combined together, these data suggest that increased Spd2 levels may alter the retention of PCM components on interphase centrosomes. This could potentially explain the loss of microtubule nucleation activity of the centrosome and its detachment from the apical cortex in interphase larval NBs.

Altered Spd2 levels partially randomise the NB division axis

Larval NBs keep their axis of division stably over multiple rounds of divisions, delivering their differentiating daughters (i.e. GMCs) always at their basal pole (Fig 3A). It has been shown that the active interphase centrosome acts as a positional cue to orient the axis of NB division (Januschke & Gonzalez, 2010). We therefore

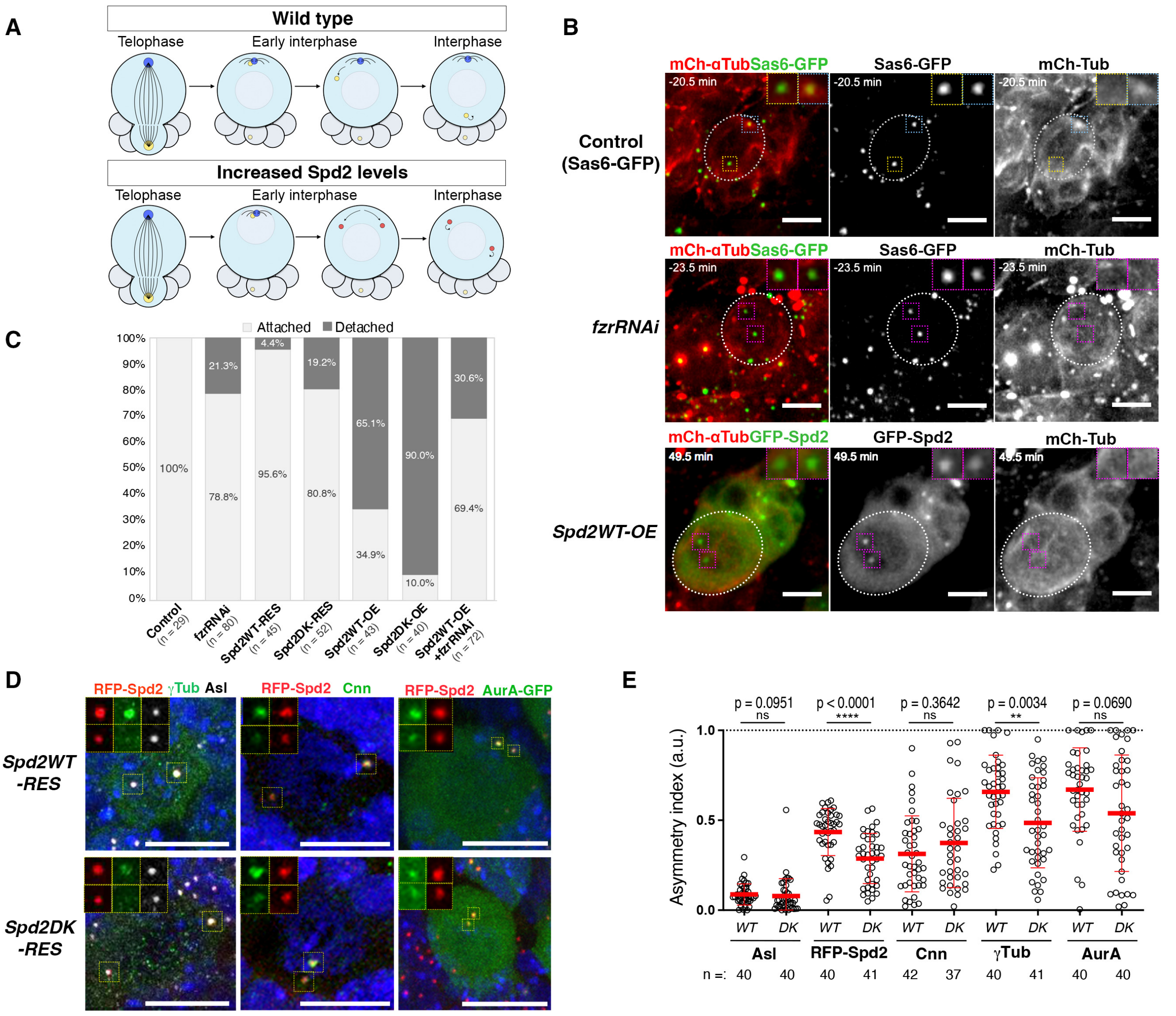

Figure 2.

◀

Figure 2. Spd2 compromises asymmetric PCM loading and activity of interphase centrosomes in larval NBs.

A A schematic diagram representing the centrosome behaviour during interphase in wild-type larval NBs and NBs with increased levels of Spd2. The daughter and mother centrosomes are represented by blue and yellow circles, respectively; the centrosomes become inactive and detached from the cortex and are represented by red circles. The size of the circles indicates the microtubule nucleation activity of the centrosome. In the wild-type NB, the daughter centrosome keeps actively nucleating microtubules and attached to the apical cortex during interphase, while in NBs with increased Spd2 levels both centrosomes become inactive and move around in the cytoplasm.

B Selected images from time-lapse movies of the control (*pUbq-Sas6-GFP*, top panels), *fzrRNAi* (carrying *pUbq-Sas6-GFP* to visualise centrosomes, middle panels) and *Spd2DK-OE* larval NBs (See also Movies EV1, EV2, EV4, and EV5). All were expressing *wor* > *mCh-αTub* to visualise microtubules. mCherry signals are shown in red and GFP signals in green. In the control, one of the two centrosome retained microtubule nucleation activity and stayed attached to the apical cortex (blue squares) while the other centrosome became inactive and moved towards the basal side (yellow squares). In *fzrRNAi* and *Spd2DK-OE* NBs, both centrosomes (orange squares) frequently became inactivated in interphase and were dissociated from the cortex and dynamically moved in the cytoplasm. Higher magnification images of the centrosomes are shown in the insets. Scale bars: 5 μm.

C In time lapse movies, NBs that showed an active centrosome that was attached to the cortex during interphase ("Attached") and those in which both centrosomes became inactivated and detached from the cortex during interphase ("Detached") were counted in third instar larval brains of the indicated fly lines and their proportions are presented in bar graphs. The numbers of NBs analysed from at least four brains (*n*) are Control: 29, *fzrRNAi*: 80, *Spd2WT-RES*: 45, *Spd2DK-RES*: 52, *Spd2WT-OE*: 43, *Spd2DK-OE*: 40, *Spd2WT-OE* + *fzrRNAi*: 72. *P*-values are *fzrRNAi*: 0.0069, *Spd2WT-RES*: 0.2479, *Spd2DK-RES*: 0.0116, *Spd2WT-OE*: < 0.0001, *Spd2DK-OE*: < 0.0001, *Spd2WT-OE* + *fzrRNAi*: 0.0008 (Pearson's chi-squared tests were performed between control and each condition).

D Representative images of NBs of immunostained *Spd2WT-RES* and *Spd2DK-RES* larval brains. Endogenous Asl, γTub or Cnn was detected by their specific antibodies. AurA was visualised *pUbq*-AurA-GFP transgene. Scale bars: 10 μm.

E The signal intensities of the indicated centriolar and PCM proteins at two centrosomes were quantified in individual interphase NBs in *Spd2WT-RES* and *Spd2DK-RES* larval brains and their asymmetric distributions of their centrosomal signals between the two centrosomes are presented as Asymmetric Indexes (see Materials and Methods) in a scatted dot plot. Red bars represent means ± SD. The numbers of NBs analysed from at least 3 different brains of *Spd2WT-RES* and *Spd2DK-RES* (*n*) were 40 and 40 for Asl, 40 and 41 for RFP-Spd2, 42 and 37 for Cnn, 40 and 41 for γTub and 40 and 40 for AurA. *P*-values were calculated performing non-parametric unpaired Mann–Whitney U tests and were shown above the plots.

tested whether reduced PCM asymmetry and apical centrosome detachment in interphase, caused by altered Spd2 levels, affect the division axis in three dimensions and analysed the deviation between consecutive divisions (Loyer & Januschke, 2018; see Materials and Methods, Fig 3B and C). Consistent with a previous report (Januschke & Gonzalez, 2010), NBs in the control brains maintained their division axis stably over consecutive divisions (28.24 ± 20.43°, n = 36, Fig 3B and C; Movies EV1 and EV7). In some of the larval brains with higher Spd2 levels, NBs showed significantly larger axis deviations: *fzrRNAi* and *Spd2DK-OE* brains, and the brains in which GFP-Spd2WT and *fzr*RNAi were co-expressed by *wor*-Gal4 (*Spd2WT-OE* + *fzrRNAi*) showed mean deviations of 39.62 ± 20.43°, 60.14 ± 38.79° and 46.00 ± 31.05° (n = 66, 16, and 74, respectively. Fig 3B and C; Movie EV8).

To determine whether these division orientation deviations are attributable to apical centrosome detachment, we separately analysed the division axis deviations in consecutive mitoses in NBs in which the apical centrosome stayed attached to the cortex in interphase and compared them with those in NBs in which it did not (Fig 3D). We observed strong correlations between the occurrence of apical centrosome detachment in interphase and larger division axis deviations in the subsequent mitosis (Fig 3D). These data show that the detachment of the apical centrosome during interphase caused by increased Spd2 levels significantly alters the division axis maintenance of larval NBs.

All together, these results strongly suggest that in larval NBs, the correct regulation of Spd2 protein levels by APC/C is critical for maintaining PCM and microtubule nucleation activity at the apical centrosome, required for NB division axis maintenance.

Spd2 accumulation compromises the segregation pattern of the daughter centrosome

Each centrosome consists of a pair of daughter and mother centrioles: the daughter centriole is generated using the mother centriole as the template. Due to the semi-conservative manner of the centriole duplication, two centrosomes present in each cell are different molecular ages: one centrosome contains the mother centriole that is older than the mother centriole of the other. Hence, they are commonly referred to as mother and daughter centrosomes (Nigg & Stearns, 2011; Blanco-Ameijeiras *et al*, 2022).

Various stem cells preferentially inherit either the mother or daughter centrosome. The function of this preferential centriole inheritance is unclear but may act as a form of cellular memory (Chen & Yamashita, 2021; Gonzalez, 2021). The *Drosophila* larval NB preferentially inherits the daughter centrosome (Conduit & Raff, 2010; Januschke *et al*, 2011). The asymmetric activity of the interphase centrosomes is thought to play a critical role in regulating this centrosome segregation pattern: it is the daughter centrosome that retains PCM and is anchored to the apical cortex in interphase, thereby being segregated into the daughter NB (Rebollo *et al*, 2007; Rusan & Peifer, 2007; Conduit & Raff, 2010; Januschke *et al*, 2011; Fig 4A).

To examine whether altered Spd2 levels affects the pattern of centrosome inheritance, we used live NB imaging and followed the segregation of centrosomes. Control NBs correctly segregated centrosomes (96.7%, n = 30, Fig 4A–C; Movies EV1 and EV7). Upon altered Spd2 levels, NBs largely segregated centrosomes correctly when the daughter centrosome kept nucleating microtubules in interphase (Fig 4C). An exception were NBs in *Spd2WT-OE* brains, in which the daughter centrosome was frequently mis-segregated into the GMC for unclear reasons (50.0%, n = 10, P = 0.0003 in comparison with control, Pearson's chi-squared test. Fig 4C).

We further addressed whether centrosome detachment upon altered Spd2 levels affects centrosome segregation. Due to rapid free movement of the detached centrosomes, it was difficult to track them throughout interphase in an unbiased fashion. Interestingly, we noticed that, in control NBs, the daughter centrosome always matures slightly earlier than the mother centrosome upon mitotic entry, starting expanding PCM and increasing microtubule nucleation (100%, n = 36, Figs 4B and EV4A and C; Movies EV1 and EV7). We also observed asynchronous maturation of two centrosomes in

 © 2023 The Authors

NBs with detached centrosomes (Figs 4A and B, and EV4A and C; Movies EV4, EV5, and EV8), although the incidence of synchronous centrosome maturation was increased in the presence of higher Spd2 levels (Fig EV4A and B; Movie EV9). We therefore assumed that the centrosome that matures earlier upon mitotic entry is the daughter centrosome.

Using this assumption, we monitored the fate of the daughter centrosome in the larval NBs with detached interphase centrosomes. Surprisingly, in the large number of the NBs in *fzrRNAi* and *Spd2DK-RES* brains, despite its detachment from the cortex, the daughter centrosome was correctly segregated to the NB (87.5%, n = 8, and 22.2%, n = 9, respectively, Fig 4D; Movies EV4 and EV5), indicating that, despite the lack of the cortical interaction, the daughter centrosome can maintain its preferential segregation pattern into the NB. However, in NBs with higher Spd2 levels, the daughter centrosome was frequently mis-segregated into the GMC (Fig 4A, B, and D; Movies EV8 and EV10). In *Spd2WT-OE* and *Spd2DK-OE* NBs, for instance, the segregation of the daughter centrosome was randomised: (50.0%, n = 12, and 65.2%, n = 23, respectively, Fig 4D). These data indicate that altered Spd2 levels

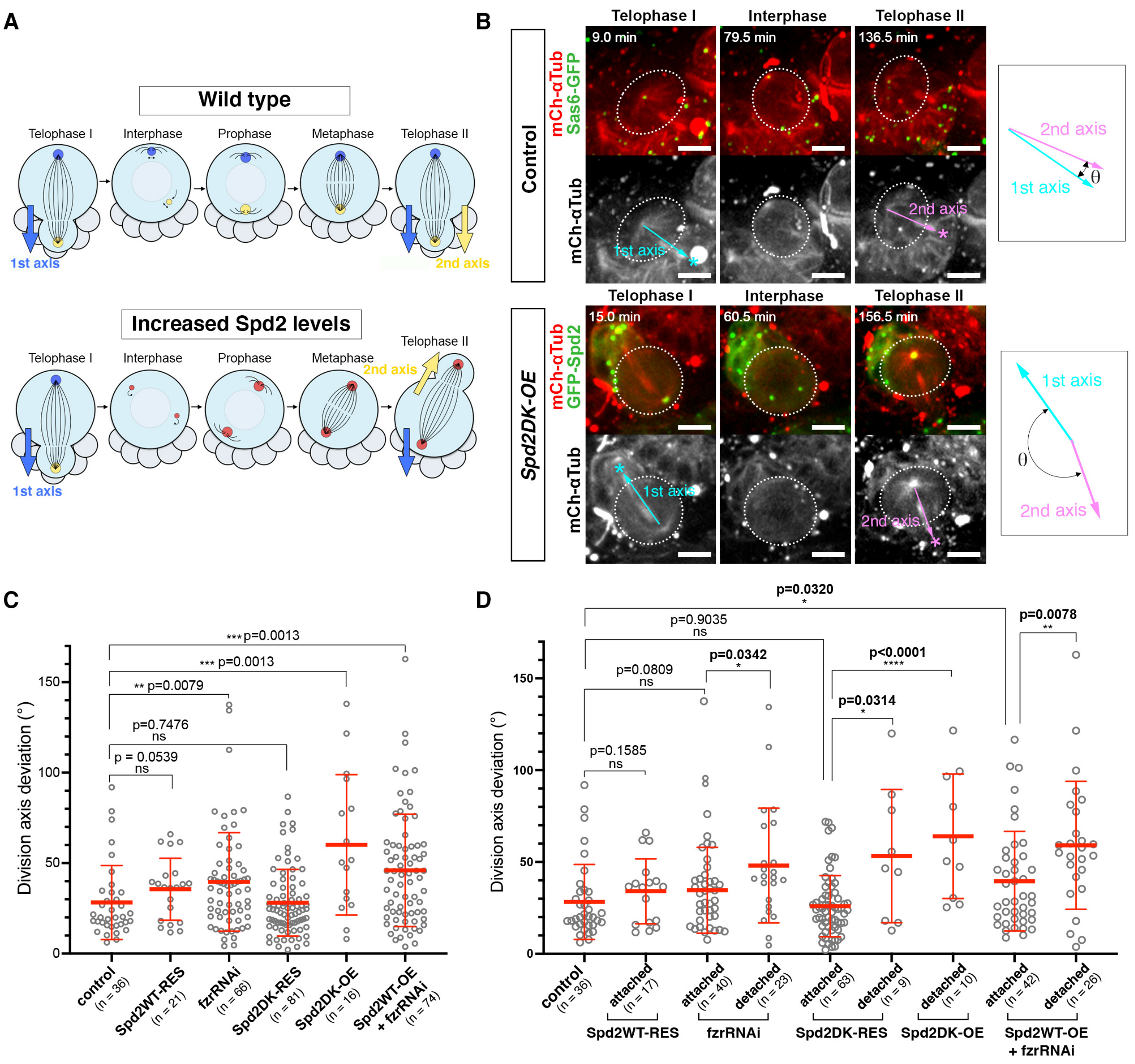

Figure 3.

◀ **Figure 3. Altered Spd2 levels partially randomise the NB division axis.**

A A diagram summarising the division axis maintenance in wild-type larval NBs and NBs with altered levels of Spd2. Wild-type NBs maintain division axes over consecutive divisions while NBs with increased Spd2 levels partially randomise division axes.

B Selected images from time-lapse microscopy of NBs in the whole-mount larval brains in the control (*pUbq-sas6-GFP*) and *Spd2WT-OE* flies (see also Movies EV7 and EV8). Centrosomes were visualised by Sas6-GFP or GFP-Spd2 signals and microtubules by mCh-αTub signals. Multi-channel images showing GFP signals in green and mCh signals in red are shown in the upper panels. Single-channel images showing mCh-αTub signals in grey are shown in the lower panels, in which dotted white lines outline NBs and asterisks indicate forming GMCs. The pale blue and pink arrows indicate the NB division axes upon the first and second mitosis, respectively, and the deviations between the two axes (θ) were determined. Control NBs mostly maintain the division axes over consecutive divisions (top panels), whereas *Spd2DK-OE* NBs often divide at significantly different angles, over 90° (lower panels). Scale bars: 10 μm.

C In larval NBs in the indicated fly brains, division angles were determined in the 3D context, and deviations of the angles in consecutive divisions were quantified (see Loyer & Januschke, 2018 and Materials and Methods for details). The number of NB divisions (n) analysed from at least four brains and the average deviations (mean ± SD) were Control: 28.24° ± 20.43 (n = 36), *Spd2WT-RES*: 35.57° ± 17.10 (n = 21), *fzrRNAi*: 39.62° ± 27.24 (n = 66), *Spd2DK-RES*: 28.13° ± 47.7 (n = 81), *Spd2DK-OE*: 60.14° ± 38.79 (n = 16), *Spd2WT-OE* combined with *fzrRNAi* (*Spd2WT-OE* + *fzrRNAi*): 46.00° ± 31.05 (n = 74). *Spd2WT-OE* brains were not analysed due to the low sample number (n = 4). P-values were calculated using Mann–Whitney U tests comparing with control and are shown above the plots. *fzrRNAi*, *Spd2DK-OE* and *Spd2WT-OE* + *fzrRNAi* showed statistically significant differences in division angle deviations.

D Division axis deviations were separately analysed in NBs with apically attached interphase centrosomes ("attached") and those with centrosomes being detached from the cortex ("detached") for each line, using the same data set as in Fig 3C. The same control data as in Fig 3C are shown for reference. *fzrRNA* detached (mean ± SD: 34.62° ± 23.39), *Spd2DK-OE* detached (64.01° ± 33.93), and *Spd2WT-OE* + *fzrRNAi* attached (39.55° ± 27.08) and detached (59.08° ± 34.88) brains showed statistically significant differences from control, while NBs with cortically attached centrosomes in any of the lines did not show significant differences. n: the number of NB divisions analysed from at least four brains for each line. P-values were calculated using Mann–Whitney U tests comparing with control and are shown above the dots.

beyond a certain threshold affect centrosome segregation in NBs independently of its detachment from the cortex.

Spd2 accumulation does not affect the centrosomal localisation of Fzr

We asked how the increase of Spd2 levels impedes PCM maintenance and microtubule nucleation at the daughter centrosome. We previously showed that Spd2 recruits Fzr to interphase centrosomes, which promotes APC/C-dependent degradation of AurA at the centrosomes (Meghini *et al*, 2016). Since AurA is a PCM regulator (Berdnik & Knoblich, 2002), increased Spd2 levels may affect PCM recruitment at interphase centrosomes through recruiting more Fzr to the centrosomes. We therefore examined whether increasing Spd2 levels can affect the centrosomal localisation of Fzr in larval NBs. As we detected Fzr by using *pUbq-GFP-Fzr* transgene, we generated fly lines in which HA-tagged wild-type Spd2 can be induced under the UAS promoter (Materials and Methods). HA-Spd2WT overexpression by *wor*-Gal4 (*HA-Spd2WT-OE*) caused centrosome detachment (26.7%, n = 30, P = 0.003, Pearson's chi-squared test, compared with control, Fig EV5A and B) and reduced an asymmetry distribution of Spd2 (Fig EV5C and E), as expected. Nevertheless, GFP-Fzr levels at interphase centrosomes and its asymmetric distribution were unaffected (Fig EV5C–E). Similarly, the dynamics of GFP-Fzr at the centrosomes during mitosis was not affected by HA-Spd2 overexpression (Fig EV5F and G; Movies EV11 and EV12). Therefore, Spd2 accumulation likely affects PCM recruitment at the daughter centrosome independently of Fzr recruitment.

Spd2 accumulation increases the mobility of Spd2 and may prevent Polo-dependent phosphorylation at the interphase centrosome

Polo/Plk1 is the conserved kinase whose activity is universally required for centrosome maturation and spindle formation in eukaryotic cells (Llamazares *et al*, 1991; Conduit *et al*, 2014a; Ramani *et al*, 2018). In interphase NBs, Polo is preferentially accumulated at the daughter centrosome, which is required and sufficient for PCM retention and microtubule nucleation activity at the centrosome (Rebollo *et al*, 2007; Rusan & Peifer, 2007; Januschke *et al*, 2013; Ramdas Nair *et al*, 2016; Gallaud *et al*, 2020). In *cnb* or *wdr62* mutant NBs, Polo cannot localise at the daughter centrosome, and both centrosomes become inactive during interphase (Ramdas Nair *et al*, 2016; Gallaud *et al*, 2020), a phenotype similar to what we observed in the NBs with high levels of Spd2 (Fig 3). We therefore examined the centrosomal localisation of Polo in interphase NBs with excess Spd2 by expressing GFP-Polo under the native *polo* promoter (Moutinho-Santos *et al*, 1999). Surprisingly, the centrosomal levels of Polo-GFP and its asymmetric centrosomal distributions were unaffected in both *Spd2DK-RES* and *HA-Spd2WT-OE* NBs (Fig 5A–D), in which the daughter centrosome was still inactivated and detached (Figs 2C and EV5B). These data suggest that Spd2 accumulation blocks PCM recruitment and microtubule nucleation at the daughter centrosome downstream of Polo.

It was recently shown that Polo directly phosphorylates Spd2 at multiple sites, which become additional binding sites for Polo (Meng *et al*, 2015; Alvarez-Rodrigo *et al*, 2019; Devi *et al*, 2021). It is proposed that Polo and Spd2 may form a positive feedback loop that enables rapid PCM expansion through an autocatalytic mechanism upon centrosome maturation (Alvarez-Rodrigo *et al*, 2019; Cabral *et al*, 2019). We speculated that a similar feedback mechanism may operate at the daughter centrosome in interphase NBs. To test this, we used Spd2CONS, the unphosphorylatable mutant form of Spd2 in which all the conserved putative Polo phosphorylation sites are mutated (Alvarez-Rodrigo *et al*, 2019). We generated fly lines in which mCherry-tagged Spd2WT or Spd2CONS is expressed in an *spd2* mutant (*spd2*^{G20143}/*Df(3L)BSC561*) background (hereinafter, referred to as *mCh-Spd2WT/CONS-RES*; Alvarez-Rodrigo *et al*, 2019). We analysed interphase NBs in *mCh-Spd2CONS-RES* third instar larval brains and found that NBs showed a significant reduction in asymmetric distribution of Spd2 and γ-Tub (Fig 5E and F), similar to the NBs with altered Spd2 levels (Fig 2D and E). This data suggests that Polo-dependent phosphorylation of Spd2 is critical for PCM retention at

© 2023 The Authors

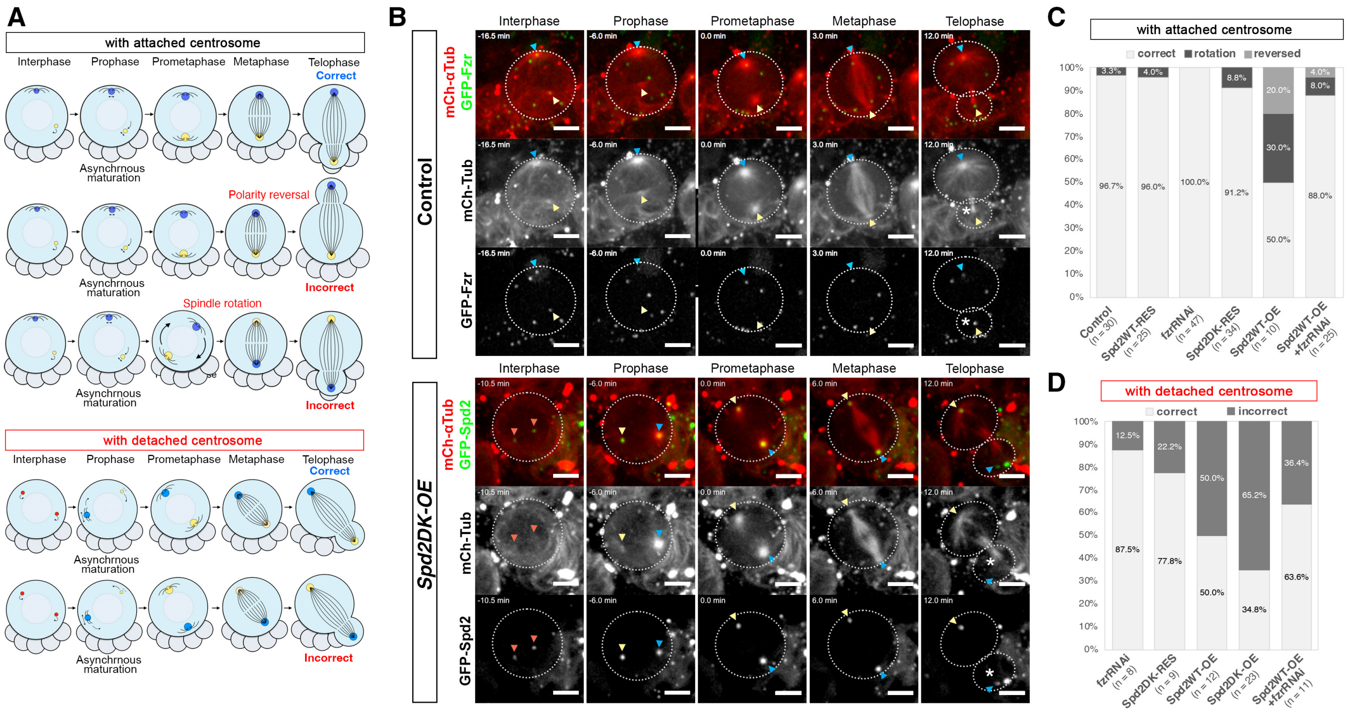

Figure 4. Spd2 accumulation compromises the segregation pattern of the daughter centrosome.

A A schematic representation of the centrosome segregation patterns of NBs with or without the detachment of the daughter centrosome from the apical cortex. The daughter and mother centrosomes are represented by blue and yellow circles, respectively; the centrosomes become inactive and detached from the cortex and are represented by red circles. In wild-type NBs, the daughter centrosome matures earlier than the daughter centrosome upon mitotic entry and is segregated into the daughter NB (top). In rare cases, NBs mis-segregated the daughter centrosome into the GMC, either by polarity reversal or spindle rotation. In most of the NBs in which both centrosomes were inactive during interphase, one centrosome (possibly, the daughter centrosome) still matured earlier than the other (see Fig EV4), like in control NBs, which can be segregated into either the daughter NB or the GMC.

B Selected images from time-lapse microscopy of NBs in control (*wor > mCh-Tub*, *pUbq-GFP-Fzr*, top panels) and *Spd2DK-OE* (lower panels) larval brains (Movies EV1 and EV10). In the control NB, the daughter centrosomes (blue arrowheads) first matured (−5.0 min) and was segregated into the daughter NB (12.0 min), while the mother centrosome (yellow arrowheads) matured a little later (0.0 min) and segregated into the GMC. In contrast, in the *Spd2DK-OE* NB, both centrosomes lost microtubule nucleation activity in interphase (red arrowheads). The centrosome that matured earlier (blue arrowheads, −6.0 min) was incorrectly segregated into the GMC (12.0 min) while the centrosome that matured later (yellow arrowheads, 0.0 min) was segregated into the NB (12.0 min). Dotted white lines outline the dividing NBs and the newly formed GMC, which are also marked by asterisks. Scale bars: 5 μm.

C, D The NB centrosome segregation patterns were quantified in each line, based on the segregation patterns in (A). The segregation patterns of the NBs with the apically attached centrosomes were analysed in (C) and those of the NBs with both centrosomes being detached in (D). *n*: the total numbers of NB divisions analysed in at least three brains in each line. Except for the *Spd2WT-OE* brains, NBs with apically attached centrosomes could correctly segregate the daughter centrosome into the daughter NBs. In *fzrRNAi* and *Spd2DK-RES* lines, most NBs with centrosome detachment could segregate the first matured centrosomes (i.e. the daughter centrosomes). However, in the brains with even higher Spd2 levels, NBs frequently mis-segregated the centrosomes.

the daughter centrosome, enabling microtubule nucleation activity and cortical association in interphase NBs.

We therefore hypothesised that excess Spd2 levels interfere with Polo-dependent phosphorylation at the daughter centrosome, thereby blocking PCM retention. We analysed the dynamics of the centrosomal association of Spd2 with the daughter centrosome by performing fluorescence recovery after photobleaching (FRAP) experiments in interphase NBs in *Spd2WT-RES* and *Spd2DK-OE* brains. We found that in *Spd2DK-OE* NBs, the Spd2 fluorescence recovered much faster and more completely than in *Spd2WT-RES* brains (Fig 5G and H; Movies EV12 and EV13). This suggests that in *Spd2DK-OE* NBs, a larger mobile Spd2 pool exists at the daughter centrosome, which may prevent accumulation of Polo-phosphorylated Spd2 on the daughter centrosome.

Discussion

In this study, we set out to identify centrosomal APC/C substrates in *Drosophila* and conducted an *in vitro* destruction screen to identify 14 candidate substrates. Although whether all the candidates are bona fide APC/C substrates remains to be tested, we demonstrated that the protein levels of Spd2, AurA, Polo, Nek2 and Asl are indeed regulated in a proteasome- and APC/C^{Fzr}-dependent manner in *D.mel-2* cells (Fig EV2). The vertebrate orthologues of Ana1, Ana4, CP110, Rcd1 and Feo contain multiple APC/C recognition motifs, some of which are evolutionally conserved, pointing to the conserved role of the APC/C-dependent proteolysis in centrosome regulation. Although we failed to obtain any evidence that human CEP192 is targeted by APC/C^{CDH1} (Meghini *et al*, 2016), it is

© 2023 The Authors

noteworthy that CEP192 has been shown to be targeted by another cell cycle–related E3 ligase, the SCF (Moser *et al*, 2013; Fung *et al*, 2018; Meitinger *et al*, 2020). Thus, although the APC/C may not take part, the cell cycle–dependent regulation of Spd2/CEP192 protein stability through the ubiquitin-proteasome pathway may be conserved through evolution.

The critical role of APC/C-dependent proteolysis in coupling the centriole duplication process to the cell cycle is widely acknowledged (Strnad *et al*, 2007; Arquint *et al*, 2012; Badarudeen *et al*, 2021). Yet, its importance beyond the duplication control is unclear. Key PCM regulators, such as Plk1 and AurA, have been known to be APC/C substrates. However, the stabilisation/accumulation of these regulators appears to have little impact on the general functions of the mitotic centrosome, i.e. PCM accumulation and bipolar spindle formation (Lindon & Pines, 2004; Floyd *et al*, 2008). In alignment with this, we also showed that overexpression of Spd2, even the non-degradable mutant form Spd2DK, does not strongly affect centrosome maturation and bipolar spindle formation (Figs EV2 and EV4). However, we found in the *Drosophila* larval NB that Spd2 protein levels strongly impact the cell type–specific interphase function of the centrosomes, which serves as a positional cue for orienting the spindle in the following mitosis. Upon Spd2 stabilisation or accumulation, both centrosomes lose PCM and become inactive during interphase, and the daughter centrosome is detached from the cortex (Fig 2). This will result in partial randomisation of the division axis and the centriole segregation pattern (Figs 3 and 4). These results strongly suggest that APC/C-dependent degradation of Spd2 is critical for the NB-specific mode of regulation of the centrosomes, more specifically, the daughter centrosome.

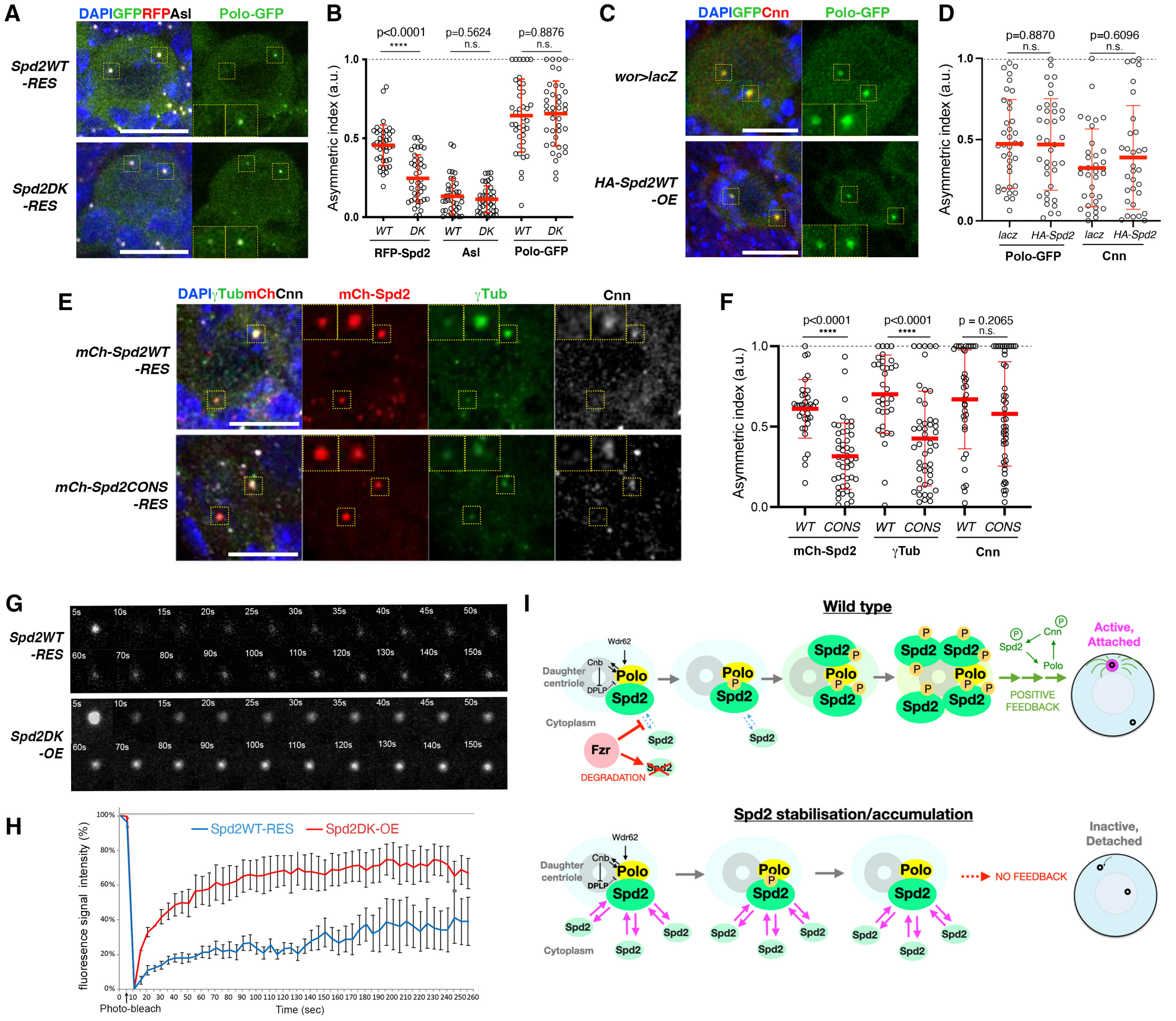

Figure 5.

 © 2023 The Authors

Figure 5. Spd2 accumulation increases the mobility of Spd2 at the interphase centrosome, preventing accumulation of Polo-dependent phosphorylation.

A Representative images of interphase NBs in fixed *Spd2WT-RES* (top panels) and *Spd2DK-RES* (lower panels) third instar larval brains expressing Polo-GFP from the native promoter and stained for DAPI and Asl. Dotted yellow squares highlight centrosomes and their magnified images are shown in insets. While Spd2 was localised more symmetrically on the two centrosomes in *Spd2DK-RES* NBs compared with *Spd2WT-RES* NBs, Polo was still asymmetrically localised, being enriched at the apical centrosome, in *Spd2DK-RES* NBs. Scale bar: 10 μm.

B Asymmetric indexes of RFP-Spd2, Asl and Polo-GFP in *Spd2WT-RES* and *Spd2DK-RES* interphase NBs expressing Polo-GFP. The asymmetric distribution of Polo-GFP at interphase centrosomes was unaffected in *Spd2DK-RES*, unlike Spd2. Forty NBs (*n*) from at least three brains were analysed in each line. Red bars represent means ± SD.

C, D Representative images of interphase NBs in fixed *wor* > *lacZ* (control, top panels) and *HA-Spd2WT-OE* larval brains expressing Polo-GFP from the native promoter and stained for DAPI and Spd2 (C). Asymmetric indexes of Polo-GFP in interphase NBs *wor* > *lacZ* (control, top panels) and *HA-Spd2WT-OE* larval brains (D). Thirty-eight and 39 NBs (*n*) from at least three brains were analysed in each line. The asymmetric distribution of Polo-GFP at interphase centrosomes was unaffected by HA-Spd2WT overexpression. Red bars represent means ± SD.

E Representative images of interphase NBs in fixed *mCh-Spd2WT-RES* (top panels) and *mCh-Spd2DK-RES* larval brains and stained for DAPI, γTub and Cnn. Both multichannel and single-channel images of mCh-Spd2, γTub and Cnn are shown. Dotted yellow squares highlight centrosomes and their magnified images are shown in insets. Similar to *Spd2DK-RES* NBs, in *mCh-Spd2CONS-RES* NBs, Spd2 and γTub were more symmetrically accumulated at the two centrosomes than in *mCh-Spd2WT-RES* NBs, but Cnn was still asymmetrically distributed. Scale bar: 10 μm.

F Asymmetric indexes of mCh-Spd2, γTub and Cnn were analysed in *mCh-Spd2WT-RES* and *mCh-Spd2CONS-RES* interphase NBs. $n = 34$ and 47 from 5 and 9 brains of each line, respectively. Red bars represent means ± SD.

G, H FRAP analyses of the centrosomal Spd2 fluorescent signals in *Spd2WT-RES* and *Spd2DK-OE* NBs. (G) Representative images from time-lapse movies (Movies EV13 and EV14) of Spd2 fluorescent signals in *Spd2WT-RES* NBs (top panels) and *Spd2DK-OE* NBs (lower panels) upon photobleaching. (H) The recoveries of the fluorescent intensities of centrosomal Spd2 signals after photobleaching were measured in *Spd2WT-RES* ($n = 7$) and *Spd2DK-OE* NBs ($n = 5$). Means ± SEM of the normalised fluorescent intensities is shown in a line graph. Centrosomal Spd2 signals recovered more fully after photobleaching in *Spd2DK-OE* NBs than in *Spd2WT-RES* NBs.

I A schematic diagram of a model for the role of APC/C^{Fzr}-dependent Spd2 degradation in the regulation of centrosomes in the *Drosophila* larval NB. The model is explained in detail in Discussion.

Data information: *P*-values in all the plots in this figure were calculated using unpaired non-parametric Mann–Whitney U-tests.

It is surprising that in the NBs with excess Spd2, the division axis is still largely maintained, despite the detachment of the daughter centrosome during interphase (Figs 2 and 3). However, this result is line with previous reports that the complete loss of the centrosome (by *sas4* or *asl* mutations) only partially affects the division orientation (Januschke & Gonzalez, 2010; Loyer & Januschke, 2018). We recently showed that the interface between the NBs and its last-born GMC provides an additional positional cue for the division axis maintenance that functions redundantly with the centrosome (Loyer & Januschke, 2018). Interestingly, we also observed that in many *Spd2DK-RES* and *fzrRNAi* NBs, despite the loss of its apical anchorage prior to mitosis, the daughter centrosome was still able to be segregated into the NB (Fig 4), which appears to be hampered by excess Spd2, suggesting an additional daughter centrosome-intrinsic mechanism that allows its preferential segregation into the NB, which involves Spd2.

How does APC/C^{Fzr}-dependent degradation of Spd2 regulate the asymmetric behaviour of the centrosomes in the larval NB? It has been shown that a daughter centriole-specific protein, Cnb, and a microtubule-associated protein, Wdr62, recruit Polo to the daughter centrosome where Polo-dependent Cnb phosphorylation is required for the retention of PCM and microtubule nucleation activity (Januschke *et al*, 2013; Ramdas Nair *et al*, 2016; Gallaud *et al*, 2020). In contrast, DPLP and CEP135 (also known as BLD10) are enriched at the mother centrosome to shed Polo and PCM (Lerit & Rusan, 2013; Singh *et al*, 2014). We observed that, in the presence of high levels of Spd2, the daughter centrosomes lose PCM and become inactive in interphase, similar to *cnb* and *wdr62* mutants (Fig 5; Januschke *et al*, 2013; Ramdas Nair *et al*, 2016; Gallaud *et al*, 2020). However, unlike in these mutants, the asymmetric localisation of Polo and Cnn appears not to be affected by the presence of excess Spd2 (Figs 2D and E, and 5A–D), indicating that Spd2 levels control the PCM retention by the daughter centrosome downstream or independently of Polo. Recently, it was shown that Spd2 is directly phosphorylated by Polo at multiple sites, which create additional binding Polo sites (Alvarez-Rodrigo *et al*, 2019). We hypothesise that, in the interphase NB, Polo-dependent Spd2 phosphorylation may allow retention of a small fraction of active PCM at the daughter centrosome. In support of this, in the NBs of *mCh-Spd2CONS-RES* brains, in which Spd2 cannot be phosphorylated by Polo, the asymmetric localisation of γ-Tubulin, but not Cnn, at the interphase centrosomes is significantly reduced (Fig 5E and F). We therefore propose a model in which APC/C^{Fzr}-dependent Spd2 proteolysis functions to facilitate the accumulation of Polo-dependent Spd2 phosphorylation at the daughter centrosome to allow its retention of microtubule nucleating capacity (Fig 5I). Importantly, it was shown that a fraction of Spd2 is dynamically localised at the centrosome (Conduit *et al*, 2014b), which would rapidly dissociate and exchange with unphosphorylated Spd2 in the cytoplasm. Our FRAP analysis showed that, in the presence of excess nondegradable Spd2 proteins, the mobile fraction of the centrosomal Spd2 pool was substantially increased (Fig 5G and H). Thus, Spd2 degradation may promote accumulation of Polo-phosphorylated Spd2 by limiting not only the total volume of Spd2 but also the mobility of the centrosomal population of Spd2 (Fig 5I). Through this dual mechanism, APC/C^{Fzr}-dependent Spd2 proteolysis can increase the sensitivity of the daughter centrosome PCM to the limited amount of Polo activity to ensure the differential activity of the two centrosomes in interphase NBs (Fig 5I). In the mitotic NB, although APC/C^{Fzr} is turned off, excess Spd2 does not interfere with PCM loading, as more abundant and more active Polo is present at the mitotic centrosome (Macůrek *et al*, 2008; Seki *et al*, 2008; Joukov *et al*, 2014).

In our previous study, we reported that Spd2 is the centrosomal loading factor of Fzr (Meghini *et al*, 2016). In this new study, we demonstrated that Spd2 is also a bona fide substrate of APC/C^{Fzr}.

© 2023 The Authors

These results suggest an Spd2-Fzr negative feedback loop which can operate to maintain the centrosomal Spd2 levels within a specific range. This mechanism can prevent excessive Spd2 accumulation at the centrosome, which may overwhelm Polo activity, while preserving a fraction of Spd2 at the inner PCM core, which is required for maintaining the structural integrity of centrioles (Loncarek *et al*, 2008; Zhu *et al*, 2008b; Seo *et al*, 2015). Interestingly, some consensus Polo sites (S-T/P) are in the vicinity of the APC/C degrons of Spd2 (Alvarez-Rodrigo *et al*, 2019). Phosphorylation of these sites may prevent the recognition of Spd2 by Fzr, thereby turning off the Spd2-Fzr negative feedback loop. Such mechanism may contribute to allow the rapid PCM expansion and the dissociation of Fzr from the centrosome upon mitotic entry.

Dysfunctions and misregulations of the centrosome are tightly linked to various human disorders, including cancer, microcephaly, and ciliopathy (Nigg & Raff, 2009; Goundiam & Basto, 2021). Our study provides evidence that defects in the ubiquitin-dependent degradation of centrosomal components can affect cell-type-specific functions of centrosomes and abrogate the behaviour of stem cells, pointing to the potential involvement of the ubiquitin-dependent regulation of the centrosome in these pathologies. It was reported that CDH1 knockout mice show a microcephaly-like phenotype and increased susceptibility to sporadic tumours (Garcí-Higuera *et al*, 2008; Delgado-Esteban *et al*, 2013; Eguren *et al*, 2013). Although these phenotypes are mainly attributed to increases genome instability due to premature S phase initiation, careful inspection of centrosomes in these mice might provide new insights on how the misregulation of centrosome components underlies some human diseases.

Materials and Methods

DNA constructs

All the sequences of *Drosophila* genes were obtained from the *Drosophila* Genomics Resource Center (DGRC) or amplified by PCR using cDNA libraries generated from fly embryos. Entry clones with the coding sequences encoding full length or fragments of these genes were generated using Gateway System (Thermo Fisher Scientific). Expression constructs were made by recombination between entry clones and the following destination vectors: pAGW (for actin5C promoter-driven N-terminal GFP fusion in *D.mel-2* cells, DGRC), pMT-N-GFP, FLAG or HA (for inducible metallothionein promoter-driven N-terminal GFP, 3xFLAG or 3xHA fusion in *D.mel-2* cells), pURW (for pUbq-driven N-terminal RFP fusion in flies) and pPHW for gal4-driven expression of N-terminal HA fusion of Spd2 (for the *HA-Spd2WT-OE* line).

The generation of the *spd2DK* mutant genes was described previously (Meghini *et al*, 2016). The over-expression plasmids for generating *Spd2WT-OE* or *Spd2DK-OE* fly lines were constructed by classic ligation as follows. *Spd2WT* or *Spd2DK* genes were amplified and N-terminally fused to eGFP gene by PCR and inserted into between NotI and XbaI sites of pUAST-attB vector (Bischof *et al*, 2007). The resulting pUAST-eGFP-Spd2WT/DK::attB plasmids were then injected into embryos of *y*, *w*, *M(eGFP*, *vas-int*, *dmRFP)* *ZH-2A; P*{*CaryP*}*attP40* lines for phiC31 integrase-mediated site-directed recombination (Groth *et al*, 2004). The candidate recombinant lines were screened by the eye colours in the progeny. The *y*, *w*, *M(eGFP*, *vas-int*, *dmRFP)ZH-2A* X chromosomes were replaced by the X chromosome carrying w^{1118} and the Gal4-mediated induction of GFP-Spd2WT/DK was confirmed by immunofluorescence and Western blotting. For generation of the fly line overexpressing HA-Spd2WT, the expression plasmid was generated by recombination between the Spd2WT entry clone and pPHW destination vector and was then injected into w^{1118} embryos for P-element-mediated recombination. The candidate recombinants were screened by the eye colours and the induction of HA-Spd2WT were confirmed by immunofluorescence and Western blotting.

His-Fzr constructs used for *in vitro* transcription and translation were generated by inserting full-length Fzr gene, N-terminally fused to 10 histidine sequences, into the pHY22 vector linearised with NcoI and BamHI restriction enzymes. The DNA sequences of all the constructs generated were confirmed by Sanger DNA sequencing (Source Bioscience).

Antibodies

The following primary antibodies were used for Western blotting and immunofluorescence: rabbit anti-Spd2 (Rodrigues-Martins *et al*, 2007), rabbit anti-Fzr (Raff *et al*, 2002), mouse anti-GFP (Sigma-Aldrich, 11814460001), mouse anti-HA (Covance HA11), mouse anti-FLAG (M2, Sigma, F3165), mouse anti-α-Tubulin (DM1A, Sigma-Aldrich), mouse anti-PSTAIRE (Sigma P7962), rabbit anti-Cnn (Bettencourt-Dias *et al*, 2005; a kind gift from Jinyang Fu), mouse anti-γ-Tubulin (GTU-88, Sigma T6557), rabbit anti-Asl (Dzhindzhev *et al*, 2010) and guinea pig anti-Asl (1:40,000 for IF, a gift from Nasser Rusan; Lerit & Rusan, 2013). The following secondary antibodies were used (all 1:1,000): Goat α-Mouse, Rabbit, Rat or Guinea Pig Alexa 488, Alexa 568, Alexa 647 or HRP (all from Life Technologies).

Drosophila strains

All flies were raised at 25°C under standard conditions unless stated otherwise. The following stocks (described in FlyBase, unless otherwise stated) were used: Oregon R (as the wild-type), $spd2^{Z3-5711}$ (Giansanti *et al*, 2008), *Df(3R)BSC561* (as spd2 deficiency), *pUbq-GFP-fzr* (Raff *et al*, 2002), *wor-gal4* (Zhu *et al*, 2008a), *UAS-mCherry-α tubulin* (a gift from Chris Doe), *UAS-fzrRNAi* (v25550, VDRC), *pUbq-AurA-GFP* (Sabino *et al*, 2011), *pPolo::polo-GFP* (Logarinho & Sunkel, 1998) and *pUbq-mCherry-Spd2WT* and *pUbq-mCherry-Spd2CONS* (Alvarez-Rodrigo *et al*, 2019). The transgenic *Spd2WT/DK-RES* fly lines were generated as described previously (Meghini *et al*, 2016). *Spd2WT-OE*, *Spd2WT-OE* and HA-Spd2WT-overexpression fly lines were generated as described above.

Drosophila cell culture and RNAi

Drosophila D.mel-2 cells (Thermo Fisher Scientific) were cultured in Express Five SFM medium (Thermo Fisher Scientific) supplemented with 2 mM L-glutamine and Pen Strep (ThermoFisher Scientific). DNA transfection was performed using FuGene HD transfection reagent (Promega) following the manufacturer's instruction. RNAi experiments were performed as described previously (Bettencourt-Dias & Goshima, 2009). Briefly, dsRNAs were prepared using the T7

 © 2023 The Authors

RiboMAX Express Large Scale RNA Production System (Promega) with cDNA templates and the following oligonucleotide primers:

T7-kanRRNAi-F: TAATACGACTCACTATAGGGAGAGACAATCTATCGCTTGTATG.

T7-kanRRNAi-R: TAATACGACTCACTATAGGGAGAGGAATCGAATGCAACCGGCGC.

T7-apc4RNAi-F: TAATACGACTCACTATAGGGAGAATGGCACAAACGAGCTCC.

T7-apc4RNAi-R: TAATACGACTCACTATAGGGAGACGCATTATCACCACCAGA.

T7-fzrRNAi-F: TAATACGACTCACTATAGGGAGAATGTTTAGTCCCGAGTAC.

T7-fzrRNAi-R: TAATACGACTCACTATAGGGAGACGCTCTGCAGGGTATGAA.

T7-rca1RNAi-F: TAATACGACTCACTATAGGGAGAGGCCACCAGGAGCAGGACCTTTACT.

T7-rca1RNAi-R: TAATACGACTCACTATAGGGAGAGCCAGGCTGCTATGGTTCGAGGTCT. dsRNA (30 mg) was then mixed with 20 μl of transfection reagent in 1 ml of medium and incubated 15 min at room temperature, then added to cells. Two rounds of dsRNA transfection were performed to efficiently deplete endogenous Apc4, Fzr or Rca1 proteins.

In vitro APC/C-dependent destruction assay

To identify potential centrosomal APC/C substrates, *in vitro* APC/C-dependent destruction assays were performed using mitotic or interphase *Xenopus laevis* egg extracts containing endogenous APC/C, as described previously (Yamano *et al*, 2009). Candidate centrosomal proteins were expressed and radioactively labelled by *in vitro* coupled transcription and translation with ^{35} S-methonines and the plasmids or PCR products in reticulocyte lysates (Promega). The ^{35} S-labelled candidate proteins were added into destruction assays reconstituted in *Xenopus* egg extracts, and their degradation was monitored at different time points. To test for APC/C^{CDC20}-dependent degradation, 0.4 mM calcium was added to *Xenopus* cytostatic factor-arrested egg extracts (CSF extracts) to release CSF inhibition and trigger rapid activation of APC/C^{CDC20}. To confirm APC/C^{CDC20} dependency, purified recombinant Mes1, the competitive APC/C inhibitor in fission yeast (Kimata *et al*, 2008) was added. To test for APC/C^{CDH1}-dependent degradation (interphase assay), purified recombinant *Xenopus* CDH1 or *Drosophila* Fzr was added into *Xenopus* interphase egg together with ^{35} S-labelled candidate proteins, initiating CDH1-dependent destruction. Aliquots were collected into 2× Laemmli buffer at the indicated time points, boiled for 2 min and resolved by SDS-PAGE. The run-on gel was detected by autoradiograph by X-ray files.

Western blots

For the Western blots of *Drosophila* brain extracts, 10 third instar larval brains were dissected in 60 μl of PBS containing Protease and Phosphatase inhibitor (Roche). The samples were lysed using a homogenising pestle (Sigma-Aldrich), and the lysates were clarified by centrifugation. 2× Laemmli buffer was added to the cleared lysates, and the samples were boiled 2 min. For *D.mel-2* cell extracts, 1 ml of confluent culture was harvested. After centrifugation, supernatents were removed, and cell pellets were washed once with 1× PBS and 200 μl of 2× Laemmli buffer was added to lyse the cells. The proteins were then resolved by SDS-PAGE. The electrophoretic run was performed using a Mini-PROTEAN Tetra Cell System (BioRad), in a Running Buffer solution (25 mM Tris, 192 mM glycine and 0.1% SDS, pH approx. 8.6, Sigma), at 200 Volts (V). The gel was assembled in a "transfer sandwich" (cushion pad-filter paper-gel-membrane-filter paper-cushion pad) and blotted on a nitrocellulose membrane (GE Healthcare) for 2 h at 60 V in Transfer Buffer solution (25 mM Tris, 190 mM Glycine, 20% Methanol). Protein transfer was verified by Ponceau S staining, and the membrane was incubated for 45 min at room temperature in a blocking solution containing 5% milk (Marvel) and 0.1% Tween (Sigma) in PBS. The membrane was then incubated in a primary antibody solution prepared in blocking solution for 1 h at room temperature. The membrane was washed three times for 10 min at room temperature with a solution of 0.1% Tween in PBS (PBST) and then incubated in a secondary antibody solution prepared in blocking solution for 1 h at room temperature. The membrane was washed three times with PBST for 10 min at room temperature, incubated with a peroxidase ECL substrate (Pierce), and the proteins were detected by exposing an X-ray film (Fuji).

Band intensity measurements

The band intensity was calculated using Gel Analyser in Image J (Schneider *et al*, 2012). A box was drawn over the band and adjusted to only include the area of interest. Boxes in the same size and shape were used for all the bands of the same protein on a film. The intensity of each band was measured after the background subtraction and normalised to the loading control (CDK1 or α-tubulin). The relative intensity was calculated by dividing it by the control value.

Immunostaining of *Drosophila* larval brains

For fixation of larval NSCs, developing adult brains were dissected from climbing third instar larvae in PBS and then transferred to the solution of 4% formaldehyde in PBS supplemented with MgCl2 and EGTA for 25 min. The brains were then washed with PBS-Triton 0.3% and pre-incubated with PBSTB (PBS containing 0.3% Triton, 3% BSA). The fixed tissues were then incubated with the primary antibodies in PBSTB overnight at 4°C. After three washes in PBSTB, samples were incubated in PBSTB with the secondary antibodies (1:1,000) and DAPI (1:1,000) for 2 h at room temperature. After three washes in PBSTB, tissue samples were mounted in Vectashield. Samples were analysed on the Nikon C2 confocal microscope.

Live imaging of *Drosophila* NBs in whole-mount larval brains

For long-term imaging of whole-mount larval brains, the samples were prepared essentially adapting the clot method previously described (Januschke & Loyer, 2020). Briefly, third instar larvae were dissected into complete Express Five SFM medium (Thermo Fisher Scientific; 2 mM L-glutamine, Pen Strep, 1 g/l glucose). The brains were included in a 10 μl droplet of complete medium pre-

warmed at 25°C and supplied with 10 mg/ml of fibrinogen and placed on a 35 mm glass bottom dish (MatTek). The brains were oriented with the dorso-anterior parts of the optic lobes facing down. The droplet was spread until the brain was slightly squashed, then 0.5 μl of 0.5 U/ml thrombin was dropped on the brain to stimulate the formation of a fibrin clot. The brains were incubated in the dark for 5–10 min to allow the formation of the clot, then they were washed three times with 200 μl of complete medium. Images were acquired on a Zeiss Axiovert200 microscope fitted with a PerkinElmer RSIII spinning disk confocal unit (PerkinElmer Life Sciences) and running the Volocity v6.3. During the acquisition, the temperature was maintained at 25°C by a stage incubator. Single optical sections were captured at 90 s intervals with a 40× lens. Data sets were imported into ImageJ and Photoshop for movie export and figure generation, respectively.

Measurement of centrosomal and cytoplasmic signal intensities and asymmetric indexes

For the signal intensity measurements, the third instar larval brains were dissected, prepared in parallel and treated the same. The control was analysed first to establish the confocal settings to be used, which remained unaltered for all images acquired. The brains were mounted with the ventral side facing at the cover slips, and confocal images of maximal 15 μm-thick z-stack images of the dorso-ventral regions of the brains were acquired with 0.5 μm intervals using a Nikon C2 confocal microscope. Signal measurement was performed using ImageJ (Schneider *et al*, 2012). In the acquired confocal images, interphase NBs were selected based on the uncondensed round morphology of DNA and maximum intensity projections of the z-stacks covering the two centrosomes were generated for individual NBs. In the projection images, two equal-sized and -shaped ROIs were selected for each of the two centrosomes (cen1 and cen2) and two in their proximity in the cytoplasm (cyto1 and cyto2) in the same NB. The mean values were measured in each of the regions, and they were defined as S^{cen1}, S^{cen2} and S^{cyto1} and S^{cyto2}. The average of S^{cyto1} and S^{cyto2} was used as cytoplasmic signals (S^{cyto}). Relative centrosome enrichment (corr.S^{cen}) of each centrosomal protein at each centrosome, the following equation was applied: corr.$S^{cen} = (S^{cen} - S^{cyto})/S^{cyto}$ (Fig EV5D). Asymmetric indexes were used to assess the degrees of asymmetric distributions of centrosomal proteins between the two centrosomes in NBs (Figs 2E and, 5B and D, and EV5E), following Lerit & Rusan (2013) with a slight modification. In the selected interphase NBs, corr. S^{cen} of each centrosomal protein at each of the two centrosomes was determined as described above. The asymmetric index (AI) in each NB was then calculated as $AI = |S^{cen1} - S^{cen2}|/(S^{cen1} + S^{cen2})$. AI = 0 indicates that the protein is equally distributed between the two centrosomes while AI = 1 indicates that the protein is specifically localised at only one of the two centrosomes.

For quantifications of the centrosomal and cytoplasmic signal intensities of Spd2 (Fig 1D), projection images of interphase NBs were obtained, and S^{cen1}, S^{cen2}, S^{cyto1} and S^{cyto2} were measured as above. However, instead of S^{cen1} and S^{cen2}, their averages were used as centrosomal signals (S^{cen}). In addition, two additional ROIs were selected outside of the NBs, and the averages of their mean values were defined as the background signal (S^{back}) and used to determine cytoplasmic signals (i.e. $S^{cyto} - S^{back}$) and for normalisation. Normalised centrosomal and cytoplasmic signal intensities of individual NBs were defined as follows: nor.$S^{cen} = (S^{cen} - S^{cyto})/S^{back}$ and nor.$S^{cyto} = (S^{cyto} - S^{back})/S^{back}$. The relative values to control (as 1.00) were plotted on Fig 1D.

For the measurement of the centrosome enrichment of GFP-Fzr signals using the time-lapse images of NBs during mitosis (Fig EV5E and F), corr.S^{cen} for the apical centrosome (one segregated into a daughter NSC after division) was determined at each time point over the time course as described above. In Fig EV5F, the values were then normalised using the highest corr.S^{cen} values in each time course as the reference.

Estimation of NB Spd2 levels in the series of fly lines

Using the normalised centrosomal and cytoplasmic signal intensities (nor.S^{cen} and nor.S^{ctyo}) measured above (Fig 1D), the approximate ratios of average Spd2 protein levels in individual NBs were estimated as control: *Spd2WT-RES*: *fzrRNAi: Spd2WT-OE* = 1: 1.00–1.18: 1.06–1.29: 1.93–3.62. Meanwhile, based on the Western blot analysis (Fig 1B and C), the ratios of the Spd2 protein levels between *Spd2WT-OE* NBs and *Spd2DK-OE* NBs and between *Spd2WT-OE* NBs and *Spd2DK-RES* NBs are*Spd2WT-OE: Spd2DK-OE* = 1: 2.04, and *Spd2WT-RES: Spd2DK-RES* = 1: 2.08, respectively. Collectively, we estimated the ratios of NB Spd2 levels between these fly lines as wild-type (or control): *Spd2WT-RES*: *fzrRNAi*: *Spd2DK-RES*: *Spd2WT-OE*: *Spd2DK-OE* = 1: 1.00–1.18: 1.06–1.29: 2.08–2.45 (= 1.00–1.18 × 2.08): 1.93–3.62: 3.94–7.38 (= 1.93–3.62 × 2.04). To note, the Spd2 levels between *Spd2DK-RES* and *Spd2WT-OE* are not directly comparable.

Measurements of NB division angles and division axis deviations

The angles of the division axes of individual NBs were determined in three dimensions as previously described (Loyer & Januschke, 2018). Briefly, the 3D coordinates of each spindle pole (centrosomes) at telophase were used to define a 3D vector of the mitotic spindle, i.e. the division axis. The angle between two 3D vectors defined for two successive divisions (the division axis deviation) was calculated using these coordinates.

Fluorescence recovery after photobleaching (FRAP) analysis of Spd2 in larval NB

For the analysis of the dynamics of centrosome signals of Spd2, third instar larvae brains were dissected in PBS and were mounted on a microscope slide with 50 μl of PBS, then slightly squashed under a coverslip to allow NBs to be physically dissociated from the brain tissue. Photobleaching and image acquisition were performed using the Nikon C2 confocal microscope at 25°C by a stage incubator. The centrosomes were identified as clearly defined spots with one or two GFP or RFP signals in each neuroblast. Photobleaching was performed with a strong pulse of the 488 nm laser (100% laser intensity) in an ROI containing only one of the centrosomes. For the analysis of the Spd2 fluorescence recovery at the centrosomes, single optical sections were captured at 5 s intervals with the 60× lens, and the corresponding FRAP curve was determined using the NIS-Elements software.

© 2023 The Authors

Image processing

The images were processed using the NIS-Elements software or ImageJ (Schneider *et al*, 2012). For better visualisation, a 0.5 Gaussian blur filter was applied to every image shown.

Statistical analyses

Statistical analysis was performed with GraphPad Prism or Microsoft Office Excel. Throughout this study, unpaired nonparametric Mann–Whitney U tests were used for data containing individual values to assess statistical significance. For results with categorical variables (e.g. presence or absence of phenotypes), *P*-values were calculated by performing Pearson's chi-squared tests and were used to assess significant differences between the data set and the control.

Data availability

This study includes no data deposited in external repositories. Original data and materials will be available from the corresponding author upon request.

Expanded View for this article is available online.

Acknowledgements
We thank Dr David Glover and his lab members for reagents, discussions and access to their spinning disc microscope, ShanghaiTech University Imaging Facility for access to microscopes, Drs Jordan Raff and Renata Basto for reagents and discussions, Dr Wei Wu at Shanghai Fly Center, Dr Simon Collier at Cambridge Fly facility, and Drs Jingnan Liu and Ji-long Liu for their help with maintenance and transporting fly stocks, Bloomington *Drosophila* Stock Center, Cambridge Fly Facility and Shanghai Fly Center, the Vienna *Drosophila* RNA Center (VDRC) and Cambridge and China *Drosophila* research communities for fly stocks, *Drosophila* colleagues at ShanghaiTech University and the University of Cambridge for discussion, sharing information and reagents. We thank all the Kimata lab members for cooperation and discussion. This work was supported by the ShanghaiTech University start-up grant (2018F0202-000-06) and the Cancer Research UK Career Development Fellowship (CRUK-Al2874) and the Natural Science Foundation of China (3217046) to Y.K., HORIZON EUROPE European Research Council Marie Curie Fellowship (655297) to T.M., and the Wellcome Trust (205150/Z/16/Z), Marie Curie Cancer Care and Worldwide Cancer Research (formerly known as AICR) grant (07-0062) to H.Y. Work in J.J.'s lab was supported by the grant (BB/V001353/1) from Biotechnology and Biological Sciences Research Council.

Author contributions
Yuu Kimata: Conceptualization; resources; formal analysis; supervision; funding acquisition; validation; investigation; visualization; methodology; writing—original draft; project administration; writing—review and editing. **Francesco Meghini:** Conceptualization; data curation; formal analysis; validation; investigation; visualization; methodology; writing—original draft; writing—review and editing. **Torcato Martins:** Conceptualization; data curation; formal analysis; funding acquisition; validation; investigation; visualization; methodology; writing—review and editing. **Qian Zhang:** Conceptualization; data curation; formal analysis; validation; investigation; visualization; writing—review and editing. **Hiroyuki Yamano:** Resources; supervision; funding acquisition; investigation; writing—review and editing. **Michelle Trickey:** Investigation. **Yusanjiang Abula:** Resources; investigation. **Jens Januschke:** Conceptualization; formal analysis; supervision; funding acquisition; investigation; methodology; writing—review and editing. **Nicolas Loyer:** Data curation; formal analysis; investigation; visualization; methodology; writing—review and editing.

Disclosure and competing interests statement
The authors declare that they have no conflict of interest.

References

Alvarez-Rodrigo I, Steinacker TL, Saurya S, Conduit PT, Baumbach J, Novak ZA, Aydogan MG, Wainman A, Raff JW (2019) Evidence that a positive feedback loop drives centrosome maturation in fly embryos. *eLife* 8: e50130

Andersen JS, Wilkinson CJ, Mayor T, Mortensen P, Nigg EA, Mann M (2003) Proteomic characterization of the human centrosome by protein correlation profiling. *Nature* 426: 570–574

Araújo SJ (2019) Centrosomes in branching morphogenesis. *Results Probl Cell Differ* 67: 323–336

Arquint C, Nigg EA (2014) STIL microcephaly mutations interfere with APC/C-mediated degradation and cause centriole amplification. *Curr Biol* 24: 351–360

Arquint C, Sonnen KF, Stierhof YD, Nigg EA (2012) Cell-cycle-regulated expression of STIL controls centriole number in human cells. *J Cell Sci* 125: 1342–1352

Badarudeen B, Anand U, Mukhopadhyay S, Manna TK (2021) Ubiquitin signaling in the control of centriole duplication. *FEBS J* 289: 4830–4849

Berdnik D, Knoblich JA (2002) Drosophila Aurora-a is required for centrosome maturation and Actin-dependent asymmetric protein localization during mitosis. *Curr Biol* 12: 640–647

Bettencourt-Dias M, Goshima G (2009) RNAi in *Drosophila* S2 cells as a tool for studying cell cycle progression. *Methods Mol Biol* 545: 39–62

Bettencourt-Dias M, Rodrigues-Martins A, Carpenter L, Riparbelli M, Lehmann L, Gatt MK, Carmo N, Balloux F, Callaini G, Glover DM (2005) SAK/PLK4 is required for centriole duplication and flagella development. *Curr Biol* 15: 2199–2207

Bischof J, Maeda RK, Hediger M, Karch F, Basler K (2007) An optimized transgenesis system for drosophila using germ-line-specific φC31 integrases. *Proc Natl Acad Sci USA* 104: 3312–3317

Blanco-Ameijeiras J, Lozano-Fernández P, Martı E (2022) Centrosome maturation - in tune with the cell cycle. *J Cell Sci* 135: jcs259395

Bornens M (2021) Centrosome organization and functions. *Curr Opin Struct Biol* 66: 199–206

Brand AH, Perrimon N (1993) Targeted gene expression as a means of altering cell fates and generating dominant phenotypes. *Development* 118: 401–415

Cabral G, Laos T, Dumont J, Dammermann A (2019) Differential requirements for centrioles in mitotic centrosome growth and maintenance. *Dev Cell* 50: 355–366.e6

Chang L, Barford D (2014) Insights into the anaphase-promoting complex: a molecular machine that regulates mitosis. *Curr Opin Struct Biol* 29: 1–9

Chen C, Yamashita YM (2021) Centrosome-centric view of asymmetric stem cell division. *Open Biol* 11: 200314

Chinen T, Yamazaki K, Hashimoto K, Fujii K, Watanabe K, Takeda Y, Yamamoto S, Nozaki Y, Tsuchiya Y, Takao D *et al* (2021) Centriole and PCM cooperatively recruit CEP192 to spindle poles to promote bipolar spindle assembly. *J Cell Biol* 220: e202006085

© 2023 The Authors

Conduit PT, Raff JW (2010) Cnn dynamics drive centrosome size asymmetry to ensure daughter centriole retention in *Drosophila* neuroblasts. *Curr Biol* 20: 2187 – 2192

Conduit PT, Feng Z, Richens JH, Baumbach J, Wainman A, Bakshi SD, Dobbelaere J, Johnson S, Lea SM, Raff JW (2014a) The centrosome-specific phosphorylation of Cnn by polo/Plk1 drives Cnn scaffold assembly and centrosome maturation. *Dev Cell* 28: 659 – 669

Conduit PT, Richens JH, Wainman A, Holder J, Vicente CC, Pratt MB, Dix CI, Novak ZA, Dobbie IM, Schermelleh L *et al* (2014b) A molecular mechanism of mitotic centrosome assembly in *Drosophila*. *eLife* 3: e03399

Conduit PT, Wainman A, Raff JW (2015) Centrosome function and assembly in animal cells. *Nat Rev Mol Cell Biol* 16: 611 – 624

Darling S, Fielding AB, Sabat-Pośpiech D, Prior IA, Coulson JM (2017) Regulation of the cell cycle and centrosome biology by deubiquitylases. *Biochem Soc Trans* 45: 1125 – 1136

Davey NE, Morgan DO (2016) Building a regulatory network with short linear sequence motifs: lessons from the degrons of the anaphase-promoting complex. *Mol Cell* 64: 12 – 23

Delgado-Esteban M, García-Higuera I, Maestre C, Moreno S, Almeida A (2013) APC/C-Cdh1 coordinates neurogenesis and cortical size during development. *Nat Commun* 4: 2879

Devi R, Pelletier L, Prosser SL (2021) Charting the complex composite nature of centrosomes, primary cilia and centriolar satellites. *Curr Opin Struct Biol* 66: 32 – 40

Dix CI, Raff JW (2007) Drosophila Spd-2 recruits PCM to the sperm centriole, but is dispensable for centriole duplication. *Curr Biol* 17: 1759 – 1764

Dobbelaere J (2015) Genome-wide RNAi screens in S2 cells to identify centrosome components. *Methods Cell Biol* 129: 279 – 300

Dobbelaere J, Josué F, Suijkerbuijk S, Baum B, Tapon N, Raff J (2008) A genome-wide RNAi screen to dissect centriole duplication and centrosome maturation in drosophila. *PLoS Biol* 6: 1975 – 1990

Dzhindzhev NS, Yu QD, Weiskopf K, Tzolovsky G, Cunha-Ferreira I, Riparbelli M, Rodrigues-Martins A, Bettencourt-Dias M, Callaini G, Glover DM (2010) Asterless is a scaffold for the onset of centriole assembly. *Nature* 467: 714 – 718

Eguren M, Porlan E, Manchado E, García-Higuera I, Cañamero M, Fariñas I, Malumbres M (2013) The APC/C cofactor Cdh1 prevents replicative stress and p53-dependent cell death in neural progenitors. *Nat Commun* 4: 2880

Feng Z, Caballe A, Wainman A, Johnson S, Haensele AFM, Cottee MA, Conduit PT, Lea SM, Raff JW (2017) Structural basis for mitotic centrosome assembly in flies. *Cell* 169: 1078 – 1089.e13

Floyd S, Pines J, Lindon C (2008) APC/CCdh1 targets Aurora kinase to control reorganization of the mitotic spindle at anaphase. *Curr Biol* 18: 1649 – 1658

Fu J, Glover DM (2012) Structured illumination of the interface between centriole and peri-centriolar material. *Open Biol* 2: 120104

Fu J, Hagan IM, Glover DM (2015) The centrosome and its duplication cycle. *Cold Spring Harb Perspect Med* 5: a015800

Fung E, Richter C, Yang H, Schäffer I, Fischer R, Kessler BM, Bassermann F, D'Angiolella V (2018) FBXL13 directs the proteolysis of CEP192 to regulate centrosome homeostasis and cell migration. *EMBO Rep* 19: e44799

Gallaud E, Pham T, Cabernard C (2017) Drosophila melanogaster neuroblasts: a model for asymmetric stem cell divisions. *Results Probl Cell Differ* 61: 183 – 210

Gallaud E, Nair AR, Horsley N, Monnard A, Singh P, Pham TT, Garcia DS, Ferrand A, Cabernard C (2020) Dynamic centriolar localization of polo and Centrobin in early mitosis primes centrosome asymmetry. *PLoS Biol* 18: e3000762

Gambarotto D, Pennetier C, Ryniawec JM, Buster DW, Gogendeau D, Goupil A, Nano M, Simon A, Blanc D, Racine V *et al* (2019) Plk4 regulates centriole asymmetry and spindle orientation in neural stem cells. *Dev Cell* 50: 11 – 24.e10

Garcí-Higuera I, Manchado E, Dubus P, Cañamero M, Méndez J, Moreno S, Malumbres M (2008) Genomic stability and tumour suppression by the APC/C cofactor Cdh1. *Nat Cell Biol* 10: 802 – 811

Giansanti MG, Bucciarelli E, Bonaccorsi S, Gatti M (2008) Drosophila SPD-2 is an essential centriole component required for PCM recruitment and astral-microtubule nucleation. *Curr Biol* 18: 303 – 309

Golsteyn RM, Mundt KE, Fry AM, Nigg EA (1995) Cell cycle regulation of the activity and subcellular localization of Plk1, a human protein kinase implicated in mitotic spindle function. *J Cell Biol* 129: 1617 – 1628

Gomez-Ferreria MA, Rath U, Buster DW, Chanda SK, Caldwell JS, Rines DR, Sharp DJ (2007) Human Cep192 is required for mitotic centrosome and spindle assembly. *Curr Biol* 17: 1960 – 1966

Gonzalez C (2021) Centrosomes in asymmetric cell division. *Curr Opin Struct Biol* 66: 178 – 182

Goundiam O, Basto R (2021) Centrosomes in disease: how the same music can sound so different? *Curr Opin Struct Biol* 66: 74 – 82

Grosskortenhaus R, Sprenger F (2002) Rca1 inhibits APC-Cdh1(Fzr) and is required to prevent cyclin degradation in G2. *Dev Cell* 2: 29 – 40

Groth AC, Fish M, Nusse R, Calos MP (2004) Construction of transgenic drosophila by using the site-specific integrase from phage phiC31. *Genetics* 166: 1775 – 1782

Haider S, Lipinszki Z, Przewloka MR, Ladak Y, D'Avino PP, Kimata Y, Lio' P, Glover DM (2015) DAPPER: a data-mining resource for protein-protein interactions. *BioData Min* 8: 30

Hoffmann I (2021) Centrosomes in mitotic spindle assembly and orientation. *Curr Opin Struct Biol* 66: 193 – 198

Januschke J, Gonzalez C (2010) The interphase microtubule aster is a determinant of asymmetric division orientation in drosophila neuroblasts. *J Cell Biol* 188: 693 – 706

Januschke J, Loyer N (2020) Applications of immobilization of drosophila tissues with fibrin clots for live imaging. *J Vis Exp* https://doi.org/10.3791/61954

Januschke J, Llamazares S, Reina J, Gonzalez C (2011) Drosophila neuroblasts retain the daughter centrosome. *Nat Commun* 2: 243

Januschke J, Reina J, Llamazares S, Bertran T, Rossi F, Roig J, Gonzalez C (2013) Centrobin controls mother-daughter centriole asymmetry in drosophila neuroblasts. *Nat Cell Biol* 15: 241 – 248

Joukov V, Walter JC, De Nicolo A (2014) The Cep192-organized aurora A-Plk1 cascade is essential for centrosome cycle and bipolar spindle assembly. *Mol Cell* 55: 578 – 591

Kimata Y, Trickey M, Izawa D, Gannon J, Yamamoto M, Yamano H (2008) A mutual inhibition between APC/C and its substrate Mes1 required for meiotic progression in fission yeast. *Dev Cell* 14: 446 – 454

King RW, Peters JM, Tugendreich S, Rolfe M, Hieter P, Kirschner MW (1995) A 20 S complex containing CDC27 and CDC16 catalyzes the mitosis-specific conjugation of ubiquitin to cyclin B. *Cell* 81: 279 – 288

Lane HA, Nigg EA (1996) Antibody microinjection reveals an essential role for human polo-like kinase 1 (Plk1) in the functional maturation of mitotic centrosomes. *J Cell Biol* 135: 1701 – 1713

Lee HS, Simon JA, Lis JT (1988) Structure and expression of ubiquitin genes of Drosophila melanogaster. *Mol Cell Biol* 8: 4727 – 4735

 © 2023 The Authors

Lee KS, Park JE, Ahn JI, Zeng Y (2021) Constructing PCM with architecturally distinct higher-order assemblies. *Curr Opin Struct Biol* 66: 66 – 73

Lerit DA, Rusan NM (2013) PLP inhibits the activity of interphase centrosomes to ensure their proper segregation in stem cells. *J Cell Biol* 202: 1013 – 1022

Lindon C, Pines J (2004) Ordered proteolysis in anaphase inactivates Plk1 to contribute to proper mitotic exit in human cells. *J Cell Biol* 164: 233 – 241

Lizarraga SB, Margossian SP, Harris MH, Campagna DR, Han AP, Blevins S, Mudbhary R, Barker JE, Walsh CA, Fleming MD (2010) Cdk5rap2 regulates centrosome function and chromosome segregation in neuronal progenitors. *Development* 137: 1907 – 1917

Llamazares S, Moreira A, Tavares A, Girdham C, Spruce BA, Gonzalez C, Karess RE, Glover DM, Sunkel CE (1991) Polo encodes a protein kinase homolog required for mitosis in drosophila. *Genes Dev* 5: 2153 – 2165

Logarinho E, Sunkel CE (1998) The drosophila POLO kinase localises to multiple compartments of the mitotic apparatus and is required for the phosphorylation of MPM2 reactive epitopes. *J Cell Sci* 111: 2897 – 2909

Loncarek J, Hergert P, Magidson V, Khodjakov A (2008) Control of daughter centriole formation by the pericentriolar material. *Nat Cell Biol* 103: 322 – 328

Loyer N, Januschke J (2018) The last-born daughter cell contributes to division orientation of drosophila larval neuroblasts. *Nat Commun* 9: 3745

Macůrek L, Lindqvist A, Lim D, Lampson MA, Klompmaker R, Freire R, Clouin C, Taylor SS, Yaffe MB, Medema RH (2008) Polo-like kinase-1 is activated by aurora a to promote checkpoint recovery. *Nature* 455: 119 – 123

Martins T, Maia AF, Steffensen S, Sunkel CE (2009) Sgt1, a co-chaperone of Hsp90 stabilizes polo and is required for centrosome organization. *EMBO J* 28: 234 – 247

Meghini F, Martins T, Tait X, Fujimitsu K, Yamano H, Glover DMDM, Kimata Y (2016) Targeting of Fzr/Cdh1 for timely activation of the APC/C at the centrosome during mitotic exit. *Nat Commun* 7: 12607

Megraw TL, Li K, Kao LR, Kaufman TC (1999) The centrosomin protein is required for centrosome assembly and function during cleavage in drosophila. *Development* 126: 2829 – 2839

Meitinger F, Ohta M, Lee KY, Watanabe S, Davis RL, Anzola JV, Kabeche R, Jenkins DA, Shiau AK, Desai A *et al* (2020) TRIM37 controls cancer-specific vulnerability to PLK4 inhibition. *Nature* 585: 440 – 446

Meng L, Park J-E, Kim T-S, Lee EH, Park S-Y, Zhou M, Bang JK, Lee KS (2015) Bimodal interaction of mammalian polo-like kinase 1 and a Centrosomal scaffold, Cep192, in the regulation of bipolar spindle formation. *Mol Cell Biol* 35: 2626 – 2640

Mennella V, Keszthelyi B, Mcdonald KL, Chhun B, Kan F, Rogers GC, Huang B, Agard DA (2012) Subdiffraction-resolution fluorescence microscopy reveals a domain of the centrosome critical for pericentriolar material organization. *Nat Cell Biol* 14: 1159 – 1168

Moser SC, Bensaddek D, Ortmann B, Maure JF, Mudie S, Blow JJ, Lamond AI, Swedlow JR, Rocha S (2013) PHD1 links cell-cycle progression to oxygen sensing through hydroxylation of the centrosomal protein Cep192. *Dev Cell* 26: 381 – 392

Moutinho-Santos T, Sampaio P, Amorim I, Costa M, Sunkel CE (1999) In vivo localisation of the mitotic POLO kinase shows a highly dynamic association with the mitotic apparatus during early embryogenesis in drosophila. *Biol Cell* 91: 585 – 596

Müller H, Schmidt D, Steinbrink S, Mirgorodskaya E, Lehmann V, Habermann K, Dreher F, Gustavsson N, Kessler T, Lehrach H *et al* (2010) Proteomic and functional analysis of the mitotic drosophila centrosome. *EMBO J* 29: 3344 – 3357

Nigg EA, Raff JW (2009) Centrioles, centrosomes, and cilia in health and disease. *Cell* 139: 663 – 678

Nigg EA, Stearns T (2011) The centrosome cycle: centriole biogenesis, duplication and inherent asymmetries. *Nat Cell Biol* 13: 1154 – 1160

Pampalona J, Januschke J, Sampaio P, Gonzalez C (2015) Time-lapse recording of centrosomes and other organelles in *Drosophila* neuroblasts. *Methods Cell Biol* 129: 301 – 315

Pelletier L, Özlü N, Hannak E, Cowan C, Habermann B, Ruer M, Müller-Reichert T, Hyman AA (2004) The *Caenorhabditis elegans* centrosomal protein SPD-2 is required for both pericentriolar material recruitment and centriole duplication. *Curr Biol* 14: 863 – 873

Pines J (2011) Cubism and the cell cycle: the many faces of the APC/C. *Nat Rev Mol Cell Biol* 12: 427 – 438

Qi F, Zhou J (2021) Multifaceted roles of centrosomes in development, health, and disease. *J Mol Cell Biol* 13: 611 – 621

Raff JW, Jeffers K, Huang JY (2002) The roles of Fzy/Cdc20 and Fzr/Cdh1 in regulating the destruction of cyclin B in space and time. *J Cell Biol* 157: 1139 – 1149

Ramani A, Mariappan A, Gottardo M, Mandad S, Urlaub H, Avidor-Reiss T, Riparbelli M, Callaini G, Debec A, Feederle R *et al* (2018) Plk1/polo phosphorylates Sas-4 at the onset of mitosis for an efficient recruitment of Pericentriolar material to centrosomes. *Cell Rep* 25: 3618 – 3630.e6

Ramdas Nair A, Singh P, Salvador Garcia D, Rodriguez-Crespo D, Egger B, Cabernard C (2016) The microcephaly-associated protein Wdr62/CG7337 is required to maintain centrosome asymmetry in drosophila neuroblasts. *Cell Rep* 14: 1100 – 1113

Rebollo E, Sampaio P, Januschke J, Llamazares S, Varmark H, González C, Gonzalez C, Rebollo E, Sampaio P, Januschke J *et al* (2007) Functionally unequal centrosomes drive spindle orientation in asymmetrically dividing drosophila neural stem cells. *Dev Cell* 12: 467 – 474

Rodrigues-Martins A, Bettencourt-Dias M, Riparbelli M, Ferreira C, Ferreira I, Callaini G, Glover DM (2007) DSAS-6 organizes a tube-like centriole precursor, and its absence suggests modularity in centriole assembly. *Curr Biol* 17: 1465 – 1472

Rusan NM, Peifer M (2007) A role for a novel centrosome cycle in asymmetric cell division. *J Cell Biol* 177: 13 – 20

Sabino D, Brown NH, Basto R (2011) *Drosophila* Ajuba is not an Aurora-A activator but is required to maintain Aurora-A at the centrosome. *J Cell Sci* 124: 1156 – 1166

Schneider CA, Rasband WS, Eliceiri KW (2012) NIH image to ImageJ: 25 years of image analysis. *Nat Methods* 9: 671 – 675

Seki A, Coppinger JA, Jang CY, Yates JR, Fang G (2008) Bora and the kinase Aurora a cooperatively activate the kinase Plk1 and control mitotic entry. *Science* 320: 1655 – 1658

Seo MY, Jang W, Rhee K (2015) Integrity of the Pericentriolar material is essential for maintaining centriole association during M phase. *PLoS One* 10: e0138905

Singh P, Ramdas Nair A, Cabernard C (2014) The centriolar protein Bld10/Cep135 is required to establish centrosome asymmetry in drosophila neuroblasts. *Curr Biol* 24: 1548 – 1555

Strnad P, Leidel S, Vinogradova T, Euteneuer U, Khodjakov A, Gönczy P (2007) Regulated HsSAS-6 levels ensure formation of a single procentriole per centriole during the centrosome duplication cycle. *Dev Cell* 13: 203 – 213

Sudakin V, Ganoth D, Dahan A, Heller H, Hershko J, Luca FC, Ruderman JV, Hershko A (1995) The cyclosome, a large complex containing cyclin-selective ubiquitin ligase activity, targets cyclins for destruction at the end of mitosis. *Mol Biol Cell* 6: 185 – 198

© 2023 The Authors

Sunkel CE, Glover DM (1988) Polo, a mitotic mutant of *Drosophila* displaying abnormal spindle poles. *J Cell Sci* 89: 25–38

Tang N, Marshall WF (2012) Centrosome positioning in vertebrate development. *J Cell Sci* 125: 4951–4961

Woodruff JB, Wueseke O, Hyman AA (2014) Pericentriolar material structure and dynamics. *Philos Trans R Soc Lond B Biol Sci* 369: 20130459

Woodruff JB, Ferreira Gomes B, Widlund PO, Mahamid J, Honigmann A, Hyman AA (2017) The centrosome is a selective condensate that nucleates microtubules by concentrating tubulin. *Cell* 169: 1066–1077.e10

Yamano H, Trickey M, Grimaldi M, Kimata Y (2009) In vitro assays for the anaphase-promoting complex/cyclosome (APC/C) in Xenopus egg extracts. *Methods Mol Biol* 545: 287–300

Zhu CC, Boone JQ, Jensen PA, Hanna S, Podemski L, Locke J, Doe CQ, O'Connor MB (2008a) *Drosophila* Activin- and the Activin-like product dawdle function redundantly to regulate proliferation in the larval brain. *Development* 135: 513–521

Zhu F, Lawo S, Bird A, Pinchev D, Ralph A, Richter C, Müller-Reichert T, Kittler R, Hyman AA, Pelletier L (2008b) The mammalian SPD-2 ortholog Cep192 regulates centrosome biogenesis. *Curr Biol* 18: 136–141

License: This is an open access article under the terms of the Creative Commons Attribution License, which permits use, distribution and reproduction in any medium, provided the original work is properly cited.

© 2023 The Authors

Expanded View Figures

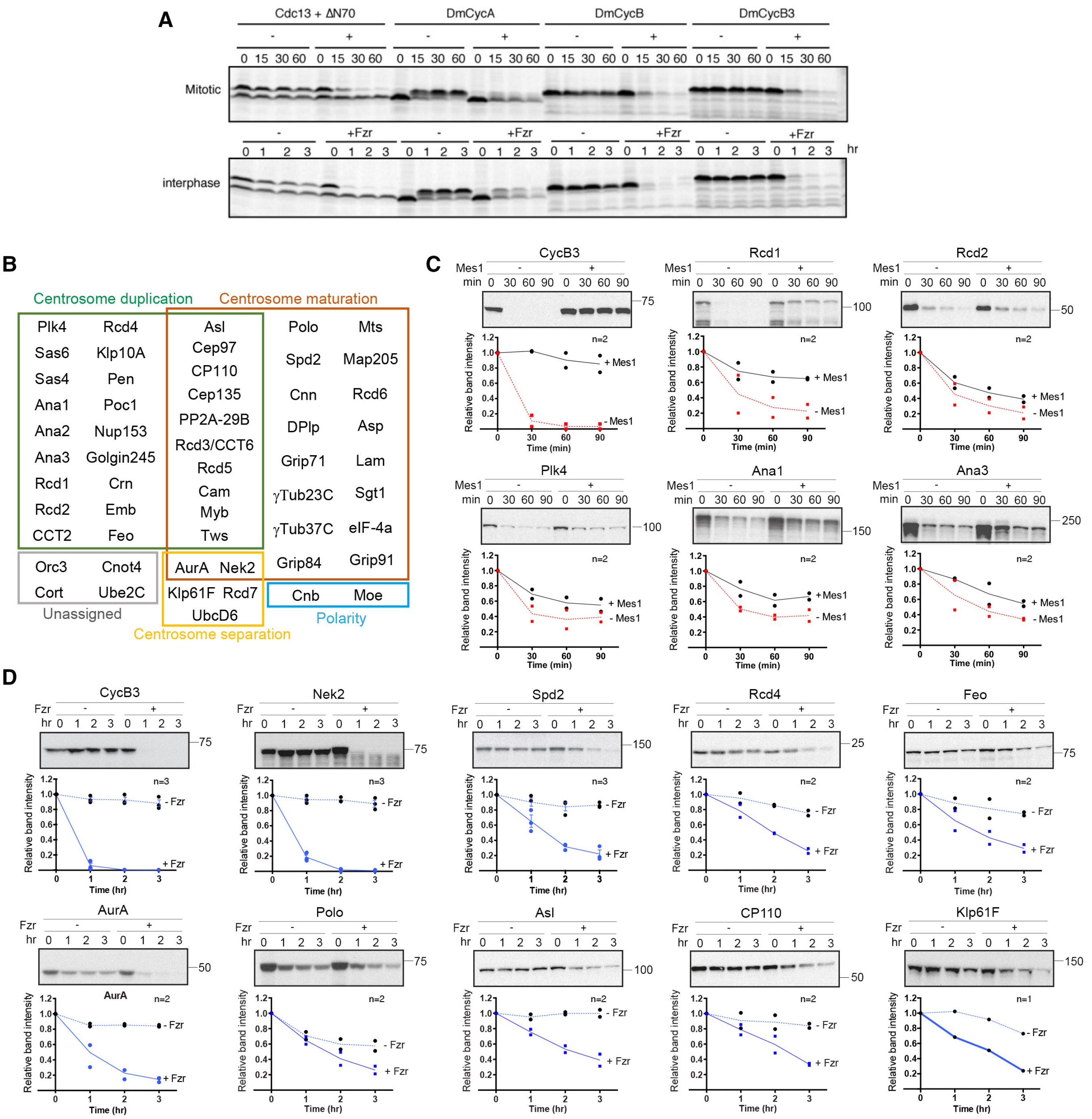

Figure EV1.

Figure EV1. The *in vitro* degradation screen identified putative centrosomal APC/C targets in *Drosophila*.

A Cell-free reconstituted APC/C-dependent degradation assays using mitotic and interphase *Xenopus leavis* egg extracts. ^{35}S-labelled substrate proteins were translated *in vitro* and were used to test their degradation. *S. pombe* mitotic cyclin Cdc13 and its stabilised mutant version ΔN70, which lacks the N-terminal 70 amino acid containing APC/C-recognition motifs, were used as positive and negative controls, respectively. All three *Drosophila* mitotic cyclins, Cyclin A (DmCycA), Cyclin B (DmCycB) and Cyclin B3 (DmCycB3), were rapidly degraded upon APC/C activation in mitotic (upper panel) and interphase egg extracts (lower panel) at similar kinetics to Cdc13.

B The cohort of *Drosophila* 55 centrosomal proteins for the *in vitro* screen. Candidate centrosomal components are grouped into different functional groups based on previous studies.

C Degradation assays of a control CycB3 and five putative APC/C^{Fzy} substrates, Rcd1, Rcd2, Plk4, Ana1 and Ana3, in mitotic egg extracts in the presence/absence of APC/C inhibitor Mes1 (See Materials and Methods for details). The representative autoradiographs are shown with molecular weight markers (kDa) in top panels, and the signal intensities of the corresponding bands at each time point on the autoradiographs were measured, and means of the relative values are shown in the line graphs, with the individual values indicated by dots, in lower panels. *n*: number of biological replicates.

D Degradation assays of CycB3 and 9 putative APC/C^{CDH1} substrates, Nek2, Spd2, Rcd4, Feo, AurA, Polo, Asl, CP110 and Klp61F, in interphase egg extracts in the presence/absence of the interphase APC/C activator Fzr. The results are presented as in (C). For data with $n = 3$, SD is shown as error bars.

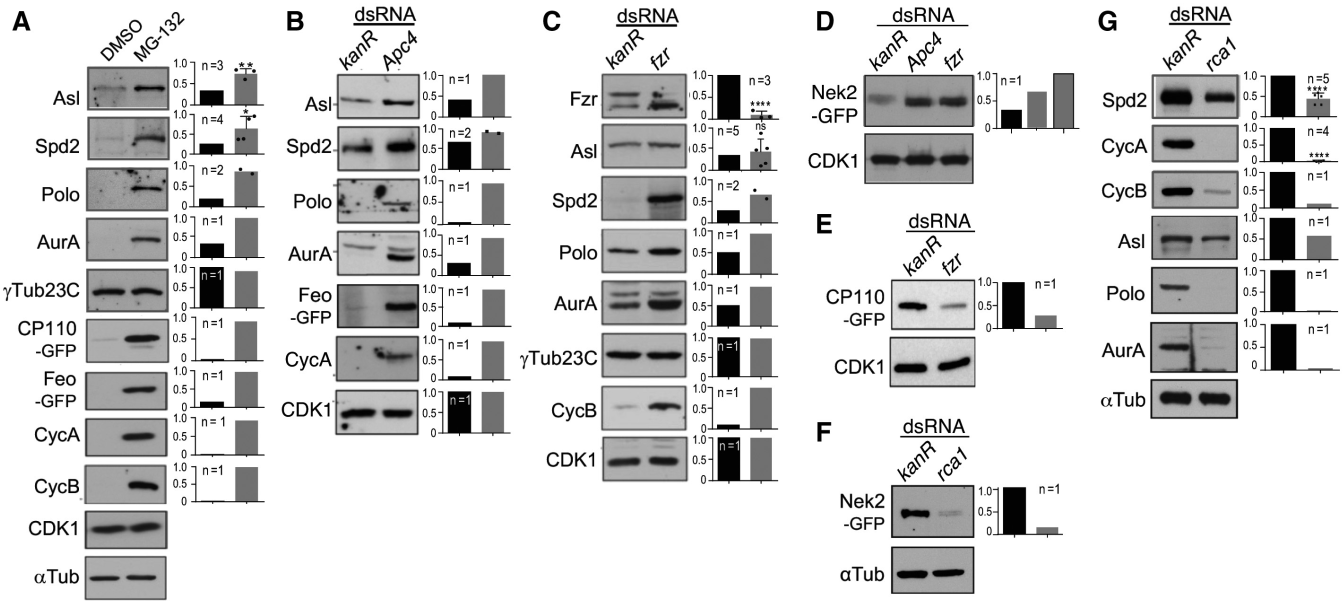

Figure EV2. type="main">APC/C^{Fzr}-dependent regulation of the protein levels of putative centrosomal APC/C substrates in cultured *Drosophila* cells.

A Effects of inhibition of the 26S proteasome by MG-132 on the levels of interphase APC/C substrate candidates in cultured *D-mel.2* cells. Examples of Western blots are presented on the left. The signal intensities of corresponding bands were measured and normalised by those of the loading controls and presented as bar graphs on the right. CDK1 and α-tubulin (αTub). CycA and CycB were used as positive controls. Proteasome inhibition caused the accumulation of endogenous Asl, Spd2, Polo and AurA proteins and transiently transfected CP110-GFP and Feo-GFP but did not affect γ-Tubulin (γTub23C). *P*-values are shown for Asl: 0.0041 (**) and for Sdp2 0.0439 (*).

B, D Effects of RNAi-mediated depletion of an APC/C subunit Apc4 on the levels of interphase candidates in cultured *D.mel-2* cells. dsRNA against the bacterial kanamycine resistant gene (*kanR*) was used as negative control for RNAi. CDK1 was used as the loading control. The accumulation of CycA was detected to confirm APC/C inactivation. Depletion of APC/C subunit Apc4 causes the accumulation of endogenous Asl, Spd2, Polo and AurA proteins and transiently transfected Feo-GFP and Nek2-GFP (D). *P*-values are indicated for Fzr: < 0.0001 (****) and for Asl: 0.6062 (ns).

C–E Effects of RNAi-mediated Fzr depletion on the levels of interphase candidates in cultured *D.mel-2* cells. CDK1 was used as the loading control. Fzr depletion results in the accumulation of endogenous Spd2, Polo and AurA proteins (C), as well as transiently transfected Nek2-GFP (D), while CP110-GFP was reduced (E).

F, G Effects of RNAi-mediated depletion of the APC/C^{Fzr} inhibitor, Rca1, on the levels of interphase candidates in cultured *D.mel-2* cells. αTub was used as the loading control. The reduction of CycA and CycB was detected to confirm the upregulation of APC/C^{Fzr} (G). Rca1 depletion causes the reduction of transiently transfected Nek2-GFP (F), as well as endogenous Spd2, Asl, Polo and AurA proteins (G), in *D.mel-2* cells. *P*-values are shown for Spd2: < 0.0001 (****) and CycA: < 0.0001 (****).

Data information: In all bar graphs in this figure, the individual values ($n = 1$) or their means ($n \geq 2$) are presented with error bars (SD, $n \geq 3$) and dots (individual values, $n \geq 2$). *n*: number of biological replicates. *P*-values were calculated using unpaird two-tailed *t*-test.
Source data are available online for this figure.

 © 2023 The Authors

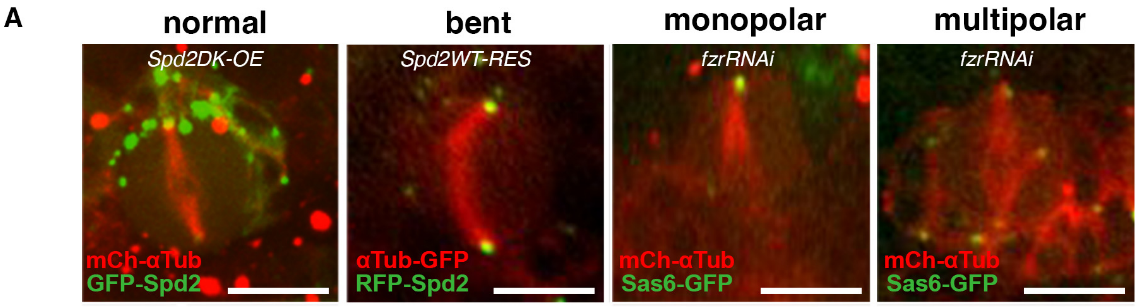

B

line name	bent spindle					transient multipolar spindle					transient monopolar spindle				
	Total (n)	phenotype	%	p-value (chi test)		Total (n)	phenotype	%	p-value (chi test)		Total (n)	phenotype	%	p-value (chi test)	
control	99	1	**1.0%**	-		100	1	**1.0%**	-		100	0	**0.0%**	-	
fzrRNAi	182	15	**8.2%**	0.0125	*	117	19	**16.2%**	0.0001	***	182	10	**5.5%**	0.0170	*
Spd2WT-RES	82	14	**17.1%**	0.0001	***	81	2	**2.5%**	0.4414	ns	81	0	**0.0%**		
Spd2DK-RES	167	25	**15.0%**	0.0002	***	163	16	**9.8%**	0.0048	**	161	0	**0.0%**		
Spd2WT-OE	17	0	**0.0%**	0.6773	ns	15	0	**0.0%**	0.6973	ns	15	0	**0.0%**		
Spd2DK-OE	45	5	**11.1%**	0.0049	**	45	1	**2.2%**	0.5594	ns	45	0	**0.0%**		
Spd2WT-OE+fzrRNAi	140	4	**2.9%**	0.3257	ns	134	4	**3.0%**	0.2989	ns	134	0	**0.0%**		

Figure EV3. Transient abnormal spindle morphologies observed in NB live imaging.

A Examples of normal bipolar spindle and transient abnormal spindle morphologies, bent spindle, monopolar spindle and multipolar spindle observed in mitotic NBs in our time-lapse live imaging. In these experiments, centrosomes and microtubules were visualised using fluorescently labelled centrosomal proteins, Sas6, Fzr or Spd2, and α-Tubulin, which are shown in red and green in this figure. Scale bars: 10 μm.

B The frequency of each of the three abnormal spindle morphologies were quantified. These abnormal spindle morphologies were observed in the NBs only for a few frames (90 fps), and the NBs then recovered normal bipolar spindle before anaphase. While fractions of *fzrRNAi*, *Spd2WT/DK-RES* and *Spd2DK-OE* NBs transiently exhibit these abnormal spindle morphologies, *Spd2WT-OE* NBs did not show these defects. *n*: total number of NB divisions analysed from at least three brains of each line. *P*-values were calculated by Pearson's chi-squared tests by comparing with control.

© 2023 The Authors

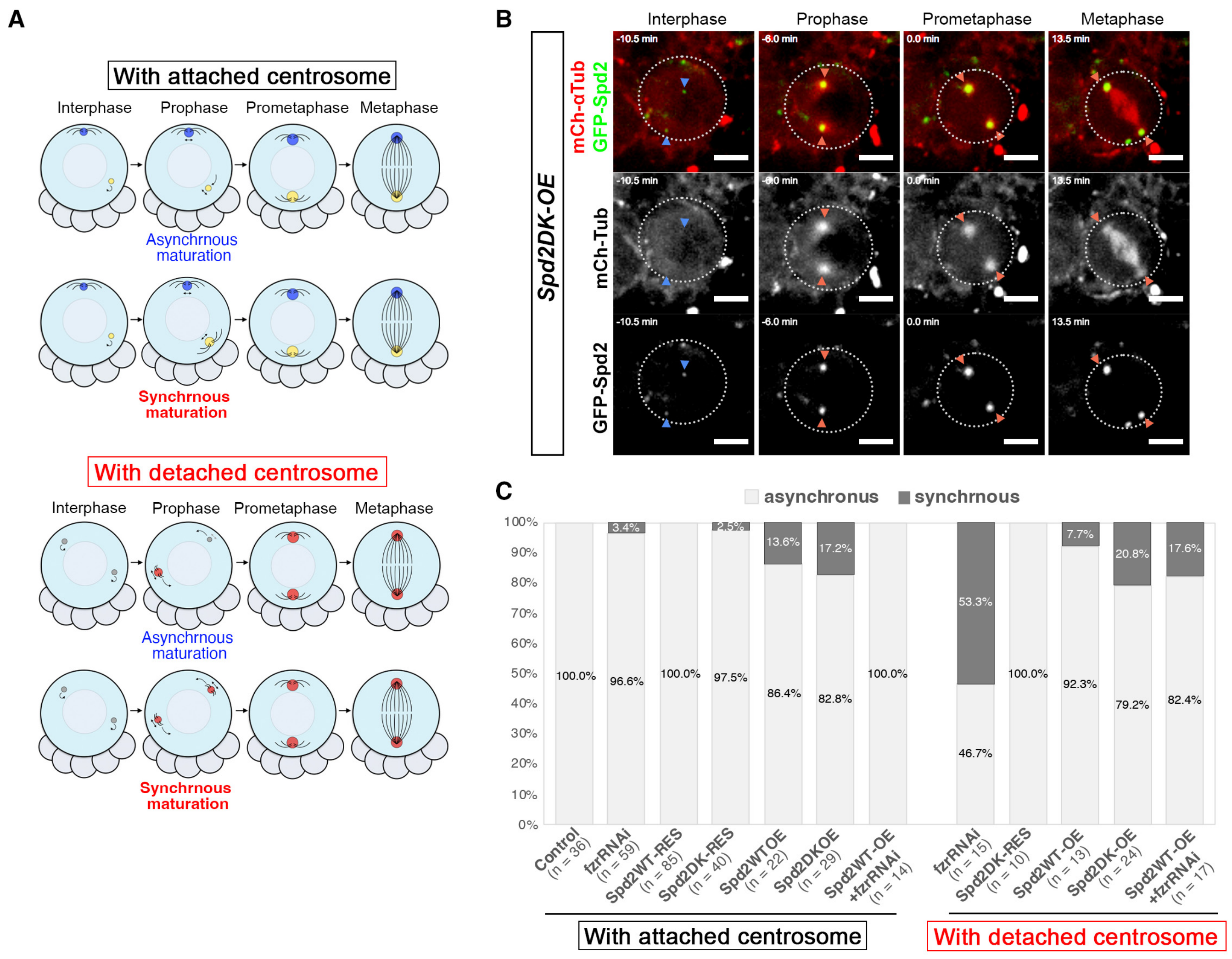

Figure EV4. Asynchronous and synchronous centrosome maturation in larval NBs with increased Spd2 levels.

A A schematic representation of asynchronous and synchronous centrosome maturation observed upon mitotic entry in the NBs in which both centrosomes become inactive and detached from the cortex in interphase due to Spd2 accumulation.

B Selected images from a time-lapse movie of *Spd2DK-OE* NBs in which two centrosomes that were inactive in interphase simultaneously expanded and started nucleating microtubules upon mitotic entry (i.e. centrosome maturation, Movie EV9). Blue arrowheads indicate inactive centrosomes, and red arrowheads indicate activated mitotic centrosomes. White dotted circles outline a NB. Scale bars: 5 µm.

C The frequencies of asynchronous and synchronous centrosome maturation observed upon mitotic entry in NBs of each of the indicated lines, with or without the apical centrosomes being detached in the preceding interphase. *n*: the number of NB divisions analysed from at least three brains of each ilne.

 © 2023 The Authors

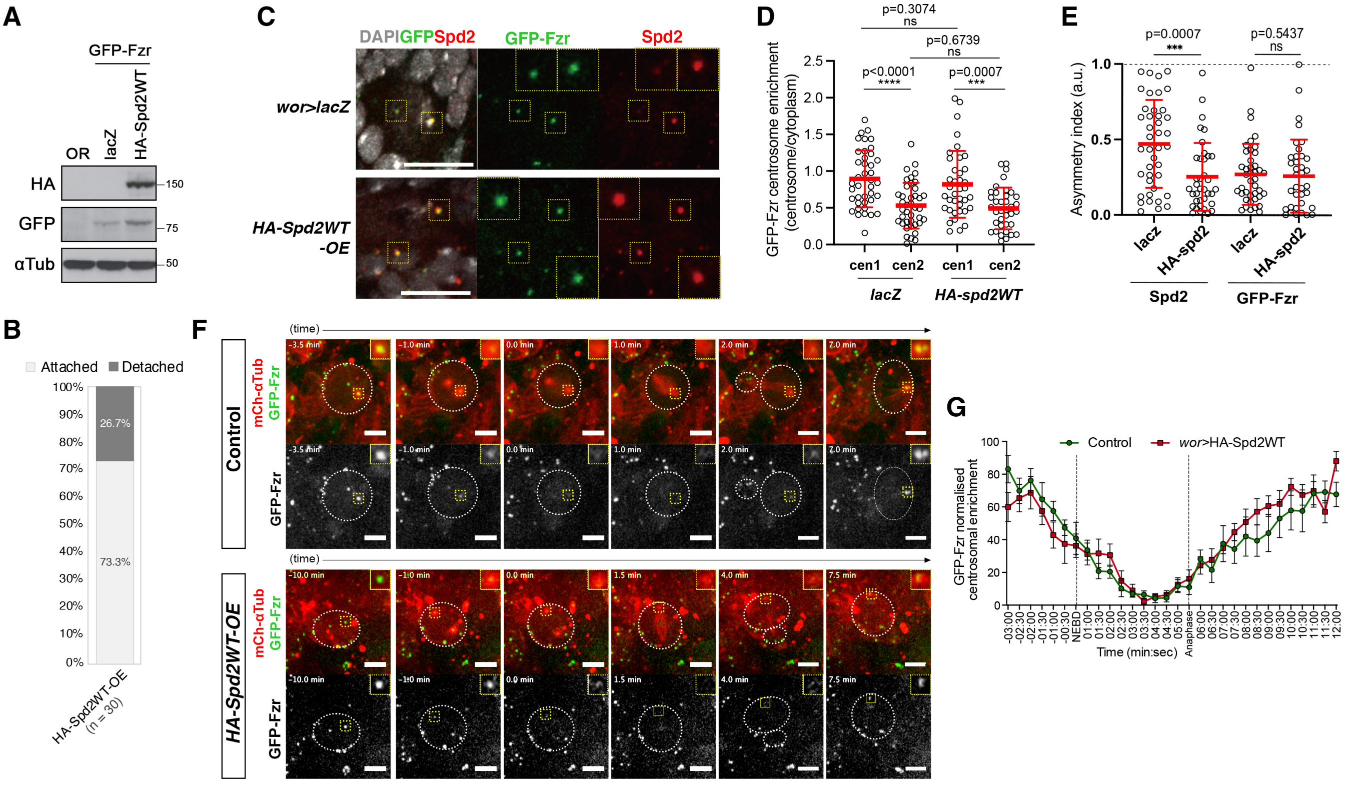

Figure EV5. The centrosomal localisation of Fzr is not affected by Spd2 accumulation.

A Western blot of the larval brain extracts from wild type (OR) or from the *pUbq-GFP-Fzr* line with NB-specific induction of lacZ (control) or HA-Spd2WT, by using HA, GFP and αTub antibodies. Expression of GFP-Fzr and HA-Spd2 was confirmed.

B In time lapse movies of third instar larval brains overexpressing HA-Spd2WT by *wor*-Gal4 (*HA-Spd2WT-OE*), the numbers of NBs that showed attached centrosomes and NBs with detached centrosomes during interphase were counted and their proportions are presented in bar graphs. *HA-Spd2WT-OE* NBs frequently showed apical centrosome detachment phenotypes. Thirty NBs (*n*) from 4 brains were analysed. *P*-values = 0.0003 (in comparison with control (Fig 2C), Pearson's chi-squared tests were performed).

C Representative images of interphase NBs in fixed control (*wor > lacZ*) and *HA-Spd2WT-OE* larval brains expressing GFP-Fzr under *pUbq* and stained for DAPI and Spd2. The dotted yellow squares indicate two centrosomes in the NBs. Scale bars: 10 μm.

D Centrosomal GFP-Fzr and Spd2 signals for each centrosome in individual interphase NBs were measured and relative signal intensities of each centrosome against cytoplasmic signals were presented in a scattered dot plots (see Materials and Methods). Among two centrosomes in each NB, the centrosome showing stronger signals was assigned as "cen1" and the other as "cen2". Centrosomal GFP-Fzr signals were not significantly affected by HA-Spd2WT overexpression. Red bars indicate means ± SD. Thirty-seven NBs (*n*) from four brains were analysed in each line.

E Asymmetric indexes of Spd2 and GFP-Fzr were calculated in control (*lacZ*) and *HA-Spd2WT-OE* NBs using the values in Fig EV5D. Asymmetric distributions of GFP-Fzr between the two centrosomes were not affected by HA-Spd2WT overexpression while those of Spd2 were affected. Red bars indicate means ± SD. Thirty-seven NBs (*n*) from four brains were analysed in each line.

F Selected images from time-lapse movies of larval NBs (in whole mount brain preparations) expressing *wor > mCh-αTub* (red) and *pUbq-GFP-Fzr* (green) alone (upper panels), or in combination with UAS-HA-Spd2WT (*HA-Spd2WT-OE*, lower panels), which were undergoing mitosis. Insets show higher magnification of the centrosomes (Movies EV11 and EV12). GFP-Fzr centrosomal dynamics were not affected by HA-Spd2WT over-expression. Scale bars correspond to 5 μm.

G Quantification of the relative centrosomal fluorescence intensity of GFP-Fzr during mitosis in control NBs and the *HA-Spd2WT-OE* NBs that showed the centrosome detachment phenotype. Eight NBs from at least three different larval brains were analysed and means ± SD are plotted in the line graph. GFP-Fzr dynamics during cell division was not affected by HA-Spd2WT overexpression.

Data information: In all statistical analyses in this figure, *P*-values were calculated using unpaired non-parametric Mann–Whitney U-tests.
Source data are available online for this figure.

© 2023 The Authors
