# Supplementary material for: APC/C‐dependent degradation of Spd2 regulates centrosome asymmetry in Drosophila neural stem cells
Source: EMBO Rep. 2023 Feb 28;24(4):e55607. doi: 10.15252/embr.202255607 (PMC10074082; doi:10.15252/embr.202255607)
Supplement: Supplementary file 19 [file EMBR-24-e55607-s001.pdf]

EMBO reports

APC/C-dependent degradation of Spd2 regulates centrosome asymmetry in *Drosophila* neural stem cells

Francesco Meghini, Torcato Martins, Qian Zhang, Nicolas Loyer, Michelle Trickey, Yusanjiang Abula, Jens Januschke, Hiroyuki Yamano, and Yuu Kimata
DOI: 10.15252/embr.202255607

Corresponding author(s): Yuu Kimata (ykimata@shanghaitech.edu.cn)

Review Timeline:

Submission Date:	17th Jun 22
Editorial Decision:	17th Jun 22
Revision Received:	9th Nov 22
Editorial Decision:	13th Dec 22
Revision Received:	31st Dec 22
Accepted:	11th Jan 23

Editor: Deniz Senyilmaz Tiebe/Achim Breiling

Transaction Report: This manuscript was transferred to EMBO reports following peer review at The EMBO Journal.

(Note: With the exception of the correction of typographical or spelling errors that could be a source of ambiguity, letters and reports are not edited. Depending on transfer agreements, referee reports obtained elsewhere may or may not be included in this compilation. Referee reports are anonymous unless the Referee chooses to sign their reports.)

Referee #1 Review
Received: 6th Jun 22

Report for Author:
The manuscript Meghini et al. describes the function of Ub-mediated degradation of a centrosomal protein, Spd2. They suggest that defects in Spd2 degradation results in erroneous activation of the inactive centrosome in Drosophila neuroblasts, interfering normal asymmetric stem cell divisions. Overall, this is an interesting story, revealing the importance of APC-mediated degradation of centrosomal components. Work is done thoroughly. However, Fig4 image quality is not to the standard, and I cannot really tell whether the results are consistent with what they described in the text (for specific issues, see below). This issue should be resolved prior to publication.

Specific comments
-Abstract: asymmetric dividing   asymmetrically dividing

-Fig3C: please provide some sort of internal control, or clarify how the centrosomal level of Spd2 could be measured between cells of different genotype. Without proper normalization, measurement cannot be accurate.

-Fig4: can you comment why there are many mChe-aTub puncta specifically in Spd2DK overexpression condition? What are they?

-The part of live imaging with dissociated cells (all supplementary, before Fig4) seems unnecessary. After all, the results are confirmed well with the whole tissue sample in Fig4, and I don't understand why they have to provide lengthy description only to say 'this might be an artifact of tissue dissociation', if the results from the whole mount tissues are nicely provided.

-Fig4: it is really hard to see Spd2-GFP localization (due to added arrows etc). Also why Spd2-GFP looks so different between 4a and 4c? (they are supposed to be the same genotype).

-Fig5b: what are they comparing?

-page 15-16: the authors are mistaken about the centrosome activities in Drosophila NSCs. In Drosophila NSCs, the mother centrosome loses MTOC activity soon after mitotic exit, then the new (daughter centrosome) becomes dominant MTOC. This misunderstanding may impact the interpretation of their data shown in Fig5.

-Fig6: they say that Spd2-OE leads to 'inheritance of the mother centrosome to the NSC', but no marker was used to tell apart mother vs. daughter. They are only showing that the 'active MTOC' goes to GMC, and they assumed that this active one is the daughter centrosome (thus opposite to the normal). I don't necessarily think that they have to use daughter centrosome marker (Cnb) to prove this point, but they should not jump on the conclusion without evidence (the data in Fig6 only shows that the active MTOC goes to GMC, instead of staying in NSC).

Referee #2 Review
Received: 9th Jun 22

Report for Author:
This manuscript reports the identification of a number of APC/C substrates involved in centrosome biology in Drosophila. Among them, Spd2 is selected for further characterization. Spd2 is targeted for degradation by APC/C at the centrosome and overexpression of Spd2 mutant forms that are insensitive to APC/C-mediated degradation result in abnormal spindle orientation and changes in the ratio of asymmetric cell divisions in Drosophila neural progenitor cells.
Overall, the manuscript contains interesting observations although I am afraid that there are major problems mostly in the novelty versus previous results, and the conclusion that the observed defects are due to Spd2 overepxression rather than lack of proper Fzr localization, as previously reported.

1. Most of the work shown in Figure 1 and Figure 2 reports the targeting of several proteins by APC/C. However, most of these proteins, and especially all the ones selected for validation are known APC/C-CDC20 or FZR targets. These figures are introductory rather than showing new data and could be eventually presented as supplementary information.

2. All the proteins selected for further analysis are known substrates of APC/C-Fzr/CDH1: Polo, AurA, ASL (Cep152), Nek2 and Spd2 (Cep192). The authors indicate (lines 176-177): "However, at present, there is no report on APC/C-dependent degradation of Spd2/CEP192 family proteins.". It is unclear the reason for this sentence as Spd2 was shown to bind Fzr, and to be targeted for proteasome degradation by the APC/C in a previous paper by the same lab (Ref. 50). From the abstract of this reference: "The localization of Fzr to the centriole during interphase depends on direct interaction with Spd2. [..] Finally, we show that Spd2 is also a novel APC/C-Fzr substrate." (Meghini et al., Nat Commun 7: 12607, 2016).

3. The section entitled "The cellular levels of the conserved PCM protein Spd2 are regulated by APC/C dependent proteolysis in vivo" and the accompanying Figure 3 reports the degradation of Spd2 by APC/C-Fzr in Drosophila, with. However, the main message including the emphasis in the degradation of the centrosome pool in vivo were already present in the 2016 paper (Figures 3, 4 and 8 in the Nat Commun 2016 paper) and the advances can be considered minimal or incremental. From the 2016 Nat Commun paper by the authors: "We directly tested this hypothesis by performing in vitro degradation assays using Xenopus egg extracts, where APC/C-dependent proteolysis can be recapitulated. We found that Spd2 was efficiently degraded on addition of recombinant Fzr proteins in interphase egg extracts, but not in mitotic egg extracts containing endogenous Fzy (Fig. 8a, Supplementary Fig. 14a-d). We also performed in vitro ubiquitination assays using purified recombinant Xenopus APC/C (ref. 45) and found that Spd2 was efficiently polyubiquinated by the APC/C (Fig. 8b). Importantly, both Spd2 degradation and ubiquitination by APC/CFzr were dependent on the presence of the D-boxes and the KEN-box in Spd2; Spd2-DK was stable in interphase egg extracts and not ubiquitinated by APC/CFzr in vitro (Fig. 8a,b, Supplementary Fig. 14e)." The section in the present paper in fact makes use of the Spd2 mutants already generated in the previous paper.

4. The novelty of the paper therefore starts in line 247 (mostly presented as Supplementary Figure 3 and Figure 4). This section describes that Spd2 accumulation results in missorientation of the mitotic spindle. Technically, these results are generated by overexpressing stable Spd2 forms. It would be important to verify that this phenotype is reproduced in Fzr-null flies.

5. The main readout in Figure 4 is the angle of the mitotic spindle. This readout is largely criticized in the field and it may be convenient to demonstrate that these changes correlate with aberrations in the anchoring of the apical centrosome to the apical membrane.

6. A major problem with the conclusions is that most data are generated with the Spd2 mutants that cannot bind Fzr. In their previous manuscript, the authors report that Spd2 is essential for recruiting APC/C-Fzr to the centrosome and, in the absence of Spd2 APC/C-Fzr targets are not properly degraded. It is therefore very difficult to conclude whether the defects reported in this manuscript are due to Spd2 increased levels or lack of proper localization of APC/C-Fzr (as described in the 2016 paper). Overexpression of the wild-type Spd2 gives intermediate results and, although the authors use these results to conclude that "Fzr mislocalisation is unlikely to be the cause of the disruption of the interphase centrosome asymmetry" (line 426-427), the same results can actually be used to conclude the opposite.

Minor:
Table 1 could be moved to Supplementary (or removed) as the proteins as listed in Fig. 1a.

Overall, I would suggest a complete reorganization of the manuscript to avoid duplication of messages already published in previous papers, and a clear demonstration of the relevance of Spd2 levels or asymmetry with independence of its function in recruiting APC/C to the centrosomes and altering levels of centrosomal APC/C substrates

Referee #3 Review
Received: 12th Jun 22

Report for Author:
The article by Meghini and colleagues entitled :"Cell cycle-controlled destruction of the core PCM Spd2 ensures centrosome asymmetry in neural stem cells", investigates the dynamics of Spd2 in spindle assembly and position in Drosophila neuroblasts. The authors identify Spd2, a PCM component, as a APC/C target among other centrosome proteins. They show that APCFzr control the levels of Spd2 at mitotic exit. They further show that if Spd2 levels are maintained throughout interphase, spindle mis-position is observed and moreover, the typical centrosome asymmetry of these cells is not maintained. This impacts daughter centriole inheritance in the neuroblast. Finally, the authors describe that lack of Spd2 degradation leads to an increase in Spd2 mobile fraction, which interrupts further PCM recruitment and microtubule nucleation, which favors retention in the neuroblast.

This is a huge study that contributes to the understanding of centrosome asymmetry establishment. It is somehow very difficult to follow. The choice of words and amount of text used, do not facilitate comprehension. From an experimental point of view, most results appeared obtained in a robust manner, although some require clarification and maybe a few experiments. A shorter version including certain clarifications would certainly increase the comprehension of this study and its impact. I am not sure I understand the model proposed because some of the data are difficult to link.

Here are the main items.

Major points

1) If increasing Spd2 levels at mitotic exit lead to mis-behaviour of centrosomes and spindles- why do the authors fail to see these in GFP-SPD2DKOE, which cannot be degraded? Like in Sup Fig2? I am asking this because to me, there seems to have fewer MTs as the signal from mCh Tub is different than the Ctrl.

2) The spindle mis-orientation described in Sup Fig 3. First, I would be careful with this nomenclature. Not really mis-orientation, more like not stably maintained though mitosis? In the end is in the same position at mitotic exit, as in time point 3.00 min for the entire mitosis. But what is remarkable is that the spindle is bent and very abnormal in shape. This should be quantified and described in a more detailed manner. In Figure 4, it is impossible to follow the mitotic spindle in the 2 lower panels.
Still on the same subject, are the authors certain that Spd2OE does not cause a spindle phenotype, in terms of morphology? If this is the case, it should be properly documented.

3) If I understood this article correctly, the authors ascribe all their phenotypes in terms of centrosome defects (inheritance of the mother vs daughter) and spindle mobility, to the fact that increase SPD2-centrosome levels inhibit the recruitment of other centrosome proteins to the daughter centriole and so microtubule nucleation. But when I look at the quantifications of Cnn, or g-tubulin in fig 5 in conditions with Spd2 and Spd2DK, the levels of Cnn at the apical are exactly the same, while g-tubulin is only slightly decreased. Maybe they were not quantified at the right moment, but this actually needs to be documented. Maybe, to prove their point, it should be important to see if after microtubule deploymerization and repolymerization at mitotic exit, the Spd2DK centrosome behavior is even worse? Or the mobility of the centrosome? Of the recovery of the soluble fraction after FRAP?

4) The differences in the FRAP experiments are impressive. Spd2-WT never reaches a strong signal. Is this normal? Could it be the problem in having stable Spd2, is not that the others are not recruited but rather that an unstable population is required for something that needs to be identified? Maybe documenting better the microtubule behavior will help clarifying this point?

Minor points:

1) The authors have to decide if it is Fzr or Cdh1. Choose one nomenclature and stick to it.

2) I count more than 55 proteins in the table provided in Fig 1a. Why is moesin added to the list? This should be explained.

3) Supplementary Fig 1 legend: were translated in vitro translated. The should be ameliorated.

4) Also, the nomenclature of all the fly strains used are difficult to follow. For example, RFP-Spd2WT-Res, this refers to the WT gene tagged with RFP and in a spd2 mutant background? To only have Spd2 from the transgene. Please explain. Is this in the Spd2 def background only?

5) The model in Fig 7e is impossible to follow.

Dear Dr. Kimata,

Thank you for transferring your manuscript to EMBO Reports, which was previously reviewed at The EMBO Journal.

Having read the manuscript and the referee reports, I would like to invite you to submit a revised manuscript to EMBO Reports as my colleague Hartmut mentioned in his previous letter. In particular,

- The study needs to be refocused and simplified as per suggestions of referees #2 and #3.
- The analysis reporting phenotypes related to mitotic spindle needs to be strengthened (referee #2, point 5; referee #3, points 1, 2, 3)
- As it stands, it is unclear whether the phenotypes are due to accumulation of Spd2 or APC/C-Fzr mislocalization. This needs to be experimentally addressed (referee #2, point 6).

Please revise your manuscript with the understanding that the referee concerns (as in their reports) must be fully addressed and their suggestions taken on board. Please address all referee concerns in a complete point-by-point response. Acceptance of the manuscript will depend on a positive outcome of a second round of review. It is EMBO reports policy to allow a single round of revision only and acceptance or rejection of the manuscript will therefore depend on the completeness of your responses included in the next, final version of the manuscript.

We generally allow three months as standard revision time. As a matter of policy, competing manuscripts published during this period will not negatively impact on our assessment of the conceptual advance presented by your study. However, we request that you contact the editor as soon as possible upon publication of any related work, to discuss how to proceed. Should you foresee a problem in meeting this three-month deadline, please let us know in advance and we may be able to grant an extension.

*** Temporary update to EMBO Press scooping protection policy:
We are aware that many laboratories cannot function at full efficiency during the current COVID-19/SARS-CoV-2 pandemic and have therefore extended our 'scooping protection policy' to cover the period required for a full revision to address the experimental issues highlighted in the editorial decision letter. Please contact the scientific editor handling your manuscript to discuss a revision plan should you need additional time, and also if you see a paper with related content published elsewhere.***

IMPORTANT NOTE: we perform an initial quality control of all revised manuscripts before re-review. Your manuscript will FAIL this control and the handling will be DELAYED if the following APPLIES:
1. A data availability section providing access to data deposited in public databases is missing (where applicable).
2. Your manuscript contains statistics and error bars based on n=2. Please use scatter plots in these cases.

You can submit the revision either as a Scientific Report or as a Research Article. For Scientific Reports, the revised manuscript can contain up to 5 main figures and 5 Expanded View figures. If the revision leads to a manuscript with more than 5 main figures it will be published as a Research Article. In this case the Results and Discussion section should be separate. If a Scientific Report is submitted, these sections have to be combined. This will help to shorten the manuscript text by eliminating some redundancy that is inevitable when discussing the same experiments twice. In either case, all materials and methods should be included in the main manuscript file

Supplementary/additional data: The Expanded View format, which will be displayed in the main HTML of the paper in a collapsible format, has replaced the Supplementary information. You can submit up to 5 images as Expanded View. Please follow the nomenclature Figure EV1, Figure EV2 etc. The figure legend for these should be included in the main manuscript document file in a section called Expanded View Figure Legends after the main Figure Legends section. Additional Supplementary material should be supplied as a single pdf labeled Appendix. The Appendix includes a table of content on the first page with page numbers, all figures and their legends. Please follow the nomenclature Appendix Figure Sx throughout the text and also label the figures according to this nomenclature. For more details please refer to our guide to authors.

Please note that for all articles published beginning 1 July 2020, the EMBO Reports reference style will change to the Harvard style for all article types. Details and examples are provided at
https://www.embopress.org/page/journal/14693178/authorguide#referencesformat

When submitting your revised manuscript, please carefully review the instructions that follow below. Failure to include requested items will delay the evaluation of your revision.

1) a .docx formatted version of the manuscript text (including legends for main figures, EV figures and tables). Please make sure that the changes are highlighted to be clearly visible.

2) individual production quality figure files as .eps, .tif, .jpg (one file per figure).

3) a .docx formatted letter INCLUDING the reviewers' reports and your detailed point-by-point responses to their comments. As part of the EMBO Press transparent editorial process, the point-by-point response is part of the Review Process File (RPF), which will be published alongside your paper. For more details on our Transparent Editorial Process, please visit our website: https://www.embopress.org/page/journal/14693178/authorguide#transparentprocess
You are able to opt out of this by letting the editorial office know (emboreports@embo.org). If you do opt out, the Review Process File link will point to the following statement: "No Review Process File is available with this article, as the authors have chosen not to make the review process public in this case."

4) a complete author checklist, which you can download from our author guidelines (<http://embor.embopress.org/authorguide>). Please insert information in the checklist that is also reflected in the manuscript. The completed author checklist will also be part of the RPF.

5) Please note that all corresponding authors are required to supply an ORCID ID for their name upon submission of a revised manuscript (<https://orcid.org/>). Please find instructions on how to link your ORCID ID to your account in our manuscript tracking system in our Author guidelines (<http://embor.embopress.org/authorguide>).

6) We replaced Supplementary Information with Expanded View (EV) Figures and Tables that are collapsible/expandable online. A maximum of 5 EV Figures can be typeset. EV Figures should be cited as 'Figure EV1, Figure EV2" etc... in the text and their respective legends should be included in the main text after the legends of regular figures.

- For the figures that you do NOT wish to display as Expanded View figures, they should be bundled together with their legends in a single PDF file called *Appendix*, which should start with a short Table of Content. Appendix figures should be referred to in the main text as: "Appendix Figure S1, Appendix Figure S2" etc. See detailed instructions regarding expanded view here: <http://embor.embopress.org/authorguide#expandedview>.

- Additional Tables/Datasets should be labeled and referred to as Table EV1, Dataset EV1, etc. Legends have to be provided in a separate tab in case of .xls files. Alternatively, the legend can be supplied as a separate text file (README) and zipped together with the Table/Dataset file.

7) We would also encourage you to include the source data for figure panels that show essential data.

Numerical data should be provided as individual .xls or .csv files (including a tab describing the data). For blots or microscopy, uncropped images should be submitted (using a zip archive if multiple images need to be supplied for one panel). Additional information on source data and instruction on how to label the files are available <http://embor.embopress.org/authorguide#sourcedata>.

8) Our journal encourages inclusion of *data citations in the reference list* to directly cite datasets that were re-used and obtained from public databases. Data citations in the article text are distinct from normal bibliographical citations and should directly link to the database records from which the data can be accessed. In the main text, data citations are formatted as follows: "Data ref: Smith et al, 2001" or "Data ref: NCBI Sequence Read Archive PRJNA342805, 2017". In the Reference list, data citations must be labeled with "[DATASET]". A data reference must provide the database name, accession number/identifiers and a resolvable link to the landing page from which the data can be accessed at the end of the reference. Further instructions are available at <http://embor.embopress.org/authorguide#datacitation>.

9) Please make sure to include a Data Availability Section before submitting your revision - if it is not applicable, make a statement that no data were deposited in a public database. Primary datasets (and computer code, where appropriate) produced in this study need to be deposited in an appropriate public database (see <http://embor.embopress.org/authorguide#dataavailability>).

Please remember to provide a reviewer password if the datasets are not yet public.

The accession numbers and database should be listed in a formal "Data Availability " section (placed after Materials & Method) that follows the model below. Please note that the Data Availability Section is restricted to new primary data that are part of this study.

Data availability

The datasets (and computer code) produced in this study are available in the following databases:

- RNA-Seq data: Gene Expression Omnibus GSE46843 (https://www.ncbi.nlm.nih.gov/geo/query/acc.cgi?acc=GSE46843)
- [data type]: [name of the resource] [accession number/identifier/doi] ([URL or identifiers.org/DATABASE:ACCESSION])

*** Note - All links should resolve to a page where the data can be accessed. ***

10) Regarding data quantification, please ensure to specify the name of the statistical test used to generate error bars and P values, the number (n) of independent experiments underlying each data point (not replicate measures of one sample), and the test used to calculate p-values in each figure legend. Discussion of statistical methodology can be reported in the materials and methods section, but figure legends should contain a basic description of n, P and the test applied.
Please note that error bars and statistical comparisons may only be applied to data obtained from at least three independent biological replicates.
Please also include scale bars in all microscopy images.

We would also welcome the submission of cover suggestions, or motifs to be used by our Graphics Illustrator in designing a cover.

11) The journal requires a statement specifying whether or not authors have competing interests (defined as all potential or actual interests that could be perceived to influence the presentation or interpretation of an article). In case of competing interests, this must be specified in your disclosure statement. Further information: https://www.embopress.org/competing-interests

I look forward to seeing a revised version of your manuscript when it is ready. Please let me know if you have questions or comments regarding the revision.

Kind regards,

Deniz Senyilmaz Tiebe

Deniz Senyilmaz Tiebe, PhD
Editor
EMBO Reports

Dear Reviewers,

Here we re-submit our manuscript with the revised title: "**APC/C-dependent degradation of Spd2 regulates centrosome asymmetry in *Drosophila* neural stem cells"** for your review.

We were quite happy to see, overall, positive comments by the reviewers and are grateful about the constructive criticisms they provided. Following their advice, we have revised our manuscript. We believe that our revised manuscript could successfully address all the concerns raised by the reviewers. According to your suggestions, we have reorganised our manuscript, making it more concise and focused. We acknowledge that the revised manuscript reads more clearly and to the point to readers.

The main changes we have made during the revision are:

1. Re-focusing our study (as suggested by Referees #2 and #3) by:
    - substantially reducing the descriptions of our *in vitro* screen (originally Fig. 1, 2) and omitting the relevant data from main figures (now Figures EV1, EV2).
    - Removing all the redundant data using squashed NB preparation (originally Fig. S2, 3) and focusing on the more physiologically relevant, whole-mount brain preparations.
    - Focusing our report on the role of Spd2 levels in the interphase centrosome activity and its links to the other phenotypes.
2. improving our characterisation of the spindle/centrosome phenotypes caused by Spd2 accumulation (as suggested by Referee #2, point 5; Referee #3, points 1, 2, 3) (this was done in collaboration with Januschke's lab, a leading lab in the research of *Drosophila* NB asymmetric division) by:
    - reanalysing NB spindle/division angles in the entire live imaging data in 3D, which enables more accurate and more quantitative analysis (New Fig. 3).
    - providing a new figure with detailed spindle anomalies (New Fig. EV3).
    - determining the links of the observed phenotypes and Spd2 accumulation.
    - updating our conclusions that the main phenotype caused by Spd2 accumulation is inactivation and subsequent detachment of the interphase centrosomes.
3. Confirming that the observed phenotypes are caused by Spd2 accumulation and not by Fzr mislocalisation (pointed by Referee #2, point 6). Therefore, to untangle the contributions of these players, we show that:

- The centrosomal localisation of GFP-Fzr is unaffected by Spd2 over-expression (HA-Spd2WT-OE condition, New Fig. EV5) in which centrosome detachment (New Fig. EV5B) and reduced PCM asymmetry were still observed (New Fig. EV5E).
- the frequencies of centrosome detachment correlate with estimated Spd2 protein levels in NBs (New Fig. 2), but not with Fzr mislocalisation, as in the condition where GFP-Fzr localization was abolished (Spd2DK-RES, Meghini et al., 2016), we did not observe a higher incidence of the phenotypes than Spd2 over-expression (please compare Spd2DK-RES data in new Fig. 2 and HA-Spd2WT-OE in Fig.EV5B).

We are very confident that our revised manuscript will suffice the reviewers. Thank you very much for your time and consideration. We are looking forward to hearing your opinion.

Please find bellow our responses to the reviewers' comments point-by-point:

Referee #1:

1. *The manuscript Meghini et al. describes the function of Ub-mediated degradation of a centrosomal protein, Spd2. They suggest that defects in Spd2 degradation results in erroneous activation of the inactive centrosome in Drosophila neuroblasts, interfering normal asymmetric stem cell divisions. Overall, this is an interesting story, revealing the importance of APC-mediated degradation of centrosomal components. Work is done thoroughly. However, Fig4 image quality is not to the standard, and I cannot really tell whether the results are consistent with what they described in the text (for specific issues, see below). This issue should be resolved prior to publication.*

>> We are very glad to have this reviewer's support for publication and are thankful for his/her acknowledgement of the importance and thoroughness of our study. According to this reviewer's suggestions, we have now replaced the old images with new images (new Fig. 3B) , which we carefully generated by selecting relevant z-planes for projection and changing to clearer labels. We believe that the new figures do clearly communicate our findings.

Specific comments:

2. *-Abstract: asymmetric dividing > asymmetrically dividing*

> > Thank you for spotting this typo. It was corrected in the new version of the manuscript.

3. *-Fig3C: please provide some sort of internal control, or clarify how the centrosomal level of Spd2 could be measured between cells of different genotype. Without proper normalization, measurement cannot be accurate.*

>> We completely agree with the reviewer and we were also aware of the importance of normalisation for comparison of signal intensities in different samples. In the original Fig.3C-G, we normalised the centrosomal and cytoplasmic signal intensities in cultured Drosophila cells using the average intensities of the background (space outside the cell), respectively. In our revised manuscript, we replaced these measurements with new signal intensity measurements in larval NBs (new Fig. 1D). For these new data, similarly, the centrosomal and cytoplasmic signal intensities were also normalised by using the average background signals (outside the NB). We think the normalisation by proximal background signals allowed to account for the differences in overall signals between samples and experimental conditions (e.g. variable staining efficiency, positional differences on the slide, etc.). The procedure of the signal intensity measurement in confocal images is described in detail in the Materials and Methods section.

4. *-Fig4: can you comment why there are many mChe-aTub puncta specifically in Spd2DK overexpression condition? What are they?*

These puncta are mostly extracellular aggregates, which were formed outside NBs by a by-product of overexpression of mCherry-fused Tubulin. As this reviewer pointed out, in original Figure 4 these aggregates may indeed appear to be more pronounced in the *Spd2DK-OE* conditions, but it was likely an artefact due to the relative positions of the NBs inside the brains and the way in which we created the projections: these aggregates did not appear to be specifically associated with any conditions (for instance, see original Fig.5c or new Fig. 2A in which mCh-Tub aggregates appear more pronounced in control than in Spd2DK-OE). To avoid confusion and to more accurately represent or observations, we have replaced the images (now new Fig. 3B). We thank the reviewer for pointing this problem to help improving the quality of our paper.

5. *-The part of live imaging with dissociated cells (all supplementary, before Fig4) seems unnecessary. After all, the results are confirmed well with the whole tissue sample in Fig4, and I don't understand why they have to provide lengthy description only to say 'this might be an artifact of tissue dissociation', if the results from the whole mount tissues are nicely provided.*

Our intention was to use these dissociated NB to present the spindle defects caused by Spd2 overexpression more clearly and in with higher resolution. Nonetheless, the results from these

assays were reproduced in the whole mount brain preprations and we have removed all the data with dissociated NBs from our revised manuscript.

6. *-Fig4: it is really hard to see Spd2-GFP localization (due to added arrows etc). Also why Spd2-GFP looks so different between 4a and 4c? (they are supposed to be the same genotype).*

> > We understand the reviewer's point and this is mainly due to positions of the NBs in the fairly thick, *Drosophila* brain tissues and the way in which each projection image was generated. For the revised version of the manuscript, we improved the image quality by carefully projecting only the z-planes of the NB that was analysed. We also changed the labels for less crowded clearer image.

7. *-Fig5b: what are they comparing?*

> > We are sorry that the labels on x-axis in original Fig. 5B were accidentally lost during the preparation of this figure. This is now shown on the new Fig.2D, where we show the Asymmetric Indexes of the centrosomal components, which represent the degree of their asymmetric distributions between the two centrosomes (first used in Lerit and Rusan, 2013. Please see the explanation of the asymmetric indexes in Materials and Methods). As reported in wild type NBs (Lerit and Rusan, 2013), PCM components Spd2, Cnn, γ-Tubulin and AurA were asymmetrically distributed between the two centrosomes in interphase Spd2WT-RES larval NBs (expressing RFP-Spd2WT at the level comparable to endogenous Spd2 levels) while the centriole protein Asl is equally distributed (as expected). We showed that, in Spd2DK-RES NBs, in which the sable mutant form of Spd2, Spd2DK was expressed in *spd2* null mutants (thus replacing endogenous Spd2 proteins), Spd2 and γ-Tubulin (and AurA, to less extent, but not Cnn) became more symmetrically distributed between the two centrosomes, indicating that their specific accumulation at the daughter centrosome is partially compromised. This result is in alignment with the result in new Fig.2A,B that Spd2 accumulation causes inactivation of the daughter centrosome.

8. *-page 15-16: the authors are mistaken about the centrosome activities in Drosophila NSCs. In Drosophila NSCs, the mother centrosome loses MTOC activity soon after mitotic exit, then the new (daughter centrosome) becomes dominant MTOC. This misunderstanding may impact the interpretation of their data shown in Fig5.*

> > We regret that we failed to clearly explain it in the original version of the manuscript and revised this description in the new version of the manuscript. The main point is that just after mitotic exit, there is only one centrosome in the renewed NB, which contains a pair of the daughter and mother centrioles (Januschke et al 2011). Soon after, the centriole pair is split into the daughter centrosome

(containing the daughter centriole) and the mother centrosome (containing the mother centriole). Then, as the reviewer described, the daughter centrosome keeps active until the next mitosis while the mother centrosome becomes inactive. We thank the reviewer for pointing this out and to clarify that this is the current model that we took into account in our interpretation of the data.

9. *-Fig6: they say that Spd2-OE leads to 'inheritance of the mother centrosome to the NSC', but no marker was used to tell apart mother vs. daughter. They are only showing that the 'active MTOC' goes to GMC, and they assumed that this active one is the daughter centrosome (thus opposite to the normal). I don't necessarily think that they have to use daughter centrosome marker (Cnb) to prove this point, but they should not jump on the conclusion without evidence (the data in Fig6 only shows that the active MTOC goes to GMC, instead of staying in NSC).*

> > This criticism is totally reasonable, and indeed we assigned the daughter centrosome based on its active MTOC status in interphase (when it kept active during interphase) or its earlier maturation timing (when it loses MTOC activity to become detached from the cortex). In fact, we have attempted the experiment that the reviewer suggested, using Cnb-YFP to mark the daughter "centriole". However, we found that Cnb could be used to distinguish daughter and mother centrosomes only until early interphase because as soon as the centrioles are duplicated, both the daughter and mother centrosomes contain a new daughter centriole and become positive for Cnb. Therefore, unless we keep tracking each centrosome throughout interphase, Cnb could not help distinguishing the daughter and the mother centrosomes in the next mitotic entry. When the centrosome is detached from the cortex (which is the case in many NBs with high Spd2 levels), it moves around rapidly in the cytoplasm, making it virtually impossible to track the centrosome for the entire interphase.

Because of this technical issue, we adopted the following criteria for assigning the daughter centrosome: 1) the daughter centrosome keeps active MTOC activity in interphase, and/or 2) it matures earlier than the mother centrosome upon mitotic entry. These criteria are based on the centrosome behaviours in control NBs (new Fig. 4, S4) and could also predict well the centrosome segregation patterns in the mutants with relatively less increased Spd2 levels (such as, *fzrRNAi* and *Spd2DK-RES,* new Fig.4C, D).

Nevertheless, the reviewer's concern is understandable since these criteria have not been experimentally proven. Therefore, in our revised manuscript, we have weakened the tone of our conclusion, making clear that our judgement is based on the above criteria.

Referee #2:

1. *This manuscript reports the identification of a number of APC/C substrates involved in centrosome biology in Drosophila. Among them, Spd2 is selected for further characterization. Spd2 is targeted for degradation by APC/C at the centrosome and overexpression of Spd2 mutant forms that are insensitive to APC/C-mediated degradation result in abnormal spindle orientation and changes in the ratio of asymmetric cell divisions in Drosophila neural progenitor cells. Overall, the manuscript contains interesting observations although I am afraid that there are major problems mostly in the novelty versus previous results, and the conclusion that the observed defects are due to Spd2 overepxression rather than lack of proper Fzr localization, as previously reported.*

>> We thank this reviewer for acknowledging potential importance of our findings and for pointing out a problem with communicating the novelty of this work in relation to our previous paper. In our revised manuscript, we provide more data and fuller reasoning to support that the phenotypes reported in this study are caused by Spd2 accumulation rather than Fzr mislocalisation, thereby showing the role of Spd2 as an APC/C^{Fzr} substrate, not as the Fzr centrosomal loading factor. We hope that our revised manuscript can convince this reviewer about the novelty of this work.

1. *Most of the work shown in Figure 1 and Figure 2 reports the targeting of several proteins by APC/C. However, most of these proteins, and especially all the ones selected for validation are known APC/C-CDC20 or FZR targets. These figures are introductory rather than showing new data and could be eventually presented as supplementary information.*

>> We agree with this reviewer's point and moved these data to supplementary information (New Figures S1 and S2). We also substantially shortened this section in the text.

2. *All the proteins selected for further analysis are known substrates of APC/C-Fzr/CDH1: Polo, AurA, ASL (Cep152), Nek2 and Spd2 (Cep192). The authors indicate (lines 176-177): "However, at present, there is no report on APC/C-dependent degradation of Spd2/CEP192 family proteins.". It is unclear the reason for this sentence as Spd2 was shown to bind Fzr, and to be targeted for proteasome degradation by the APC/C in a previous paper by the same lab (Ref. 50). From the abstract of this reference: "The localization of Fzr to the centriole during interphase depends on direct interaction with Spd2. [..] Finally, we show that Spd2 is also a novel APC/C-Fzr substrate." (Meghini et al., Nat Commun 7: 12607, 2016).*

>>> This comment (as well as the next comment) is totally reasonable. Indeed, in our previous paper, we showed data that suggest that Spd2 is an APC/C substrate. However, the data shown in this paper were only *in vitro* or in cell culture, and we did not know how Spd2 protein levels were controlled by APC/C *in vivo* and the biological relevance of this regulation. In this new study, we demonstrated that Spd2 levels are indeed subject to APC/C-dependent regulation in developing *Drosophila* larval brains (new Fig.1) and the main consequences of the Spd2 deregulation for neural stem cell biology. We therefore revised the text to clarify the novel point of this data.

3. *The section entitled "The cellular levels of the conserved PCM protein Spd2 are regulated by APC/C dependent proteolysis in vivo" and the accompanying Figure 3 reports the degradation of Spd2 by APC/C-Fzr in Drosophila, with. However, the main message including the emphasis in the degradation of the centrosome pool in vivo were already present in the 2016 paper (Figures 3, 4 and 8 in the Nat Commun 2016 paper) and the advances can be considered minimal or incremental. From the 2016 Nat Commun paper by the authors: "We directly tested this hypothesis by performing in vitro degradation assays using Xenopus egg extracts, where APC/C-dependent proteolysis can be recapitulated. We found that Spd2 was efficiently degraded on addition of recombinant Fzr proteins in interphase egg extracts, but not in mitotic egg extracts containing endogenous Fzy (Fig. 8a, Supplementary Fig. 14a-d). We also performed in vitro ubiquitination assays using purified recombinant Xenopus APC/C (ref. 45) and found that Spd2 was efficiently polyubiquinated by the APC/C (Fig. 8b). Importantly, both Spd2 degradation and ubiquitination by APC/CFzr were dependent on the presence of the D-boxes and the KEN-box in Spd2; Spd2-DK was stable in interphase egg extracts and not ubiquitinated by APC/CFzr in vitro (Fig. 8a,b, Supplementary Fig. 14e)." The section in the present paper in fact makes use of the Spd2 mutants already generated in the previous paper.*

>>> We think this is the continuation of the above point. We have addressed this concern above. We thank this reviewer for carefully reading our manuscripts and pointing this out.

4. *The novelty of the paper therefore starts in line 247 (mostly presented as Supplementary Figure 3 and Figure 4). This section describes that Spd2 accumulation results in missorientation of the mitotic spindle. Technically, these results are generated by overexpressing stable Spd2 forms. It would be important to verify that this phenotype is reproduced in Fzr-null flies.*

>> This reviewer is right in mentioning that the main results were presented from Figure 4 onwards in our original manuscript. In our revised manuscript we have reorganised the manuscript, substantially shortening the introductory part and focusing mostly on the novelty of our findings.

As this reviewer also pointed out, most of the image data presented in our figures used overexpression of Spd2DK. The main reason underlying this selection was the higher phenotypic strength. However, these phenotypes were not observed only in the presence of Spd2DK at non-physiological levels. Although less frequent or less severe, the centrosome detachment and division axis deviation phenotypes were also observed in the other conditions, including *fzrRNAi* and *Spd2DK-RES,* with statistically significant differences compared to controls (new Figs. 2-4).

We also acknowledge the reviewer suggestion to use the *Fzr* null mutants in our experiments and we considered that experiment. However, *fzr* null mutants are embryonic lethal and even if considering a clonal analysis approach, the complete knockout of Fzr would abrogate NB cell cycle, inducing endoreplication and centrosome application. Thus, we consider that the best possible approach was to decrease the Fzr levels by using *fzr* RNAi driven by the neuroblast specific driver (*wor*-Gal4).

5. *The main readout in Figure 4 is the angle of the mitotic spindle. This readout is largely criticized in the field and it may be convenient to demonstrate that these changes correlate with aberrations in the anchoring of the apical centrosome to the apical membrane.*

We thank the reviewer for this insightful comment. In response to this comment, we consulted Jens Januschke, one of the leading experts in cell polarity research, to discuss this issue. This led to a new collaboration between us in which we have reanalysed the entire time-lapse NB image data, analysing spindle angles and division axis in 3 dimensions (Loyer and Januschke, 2018, please also see the new Materials and Methods). This new analysis enabled more accurate and detailed descriptions of the effects of altered Spd2 levels on spindle orientation and division axis maintenance (new Fig. 3), which showed that altered Spd2 levels/stability causes significant changes in division axis deviations (new Fig.3) but does not cause statistically significant changes in the spindle angle within mitosis (shown in Figure 1, below. This data is not included in our revised manuscript).

With these new data from the re-analysis, we followed the reviewer's suggestion and analysed correlations between these changes with the detachment of the centrosome from the apical cortex. We found that tight correlations between the occurrence of centrosome detachment in interphase and larger deviations of division angles in the following mitosis (new Fig. 3D), suggesting that centrosome detachment may affect the maintenance of the division axis. We appreciate the reviewer's suggestion which helped us improve our manuscript and obtain the important new data.

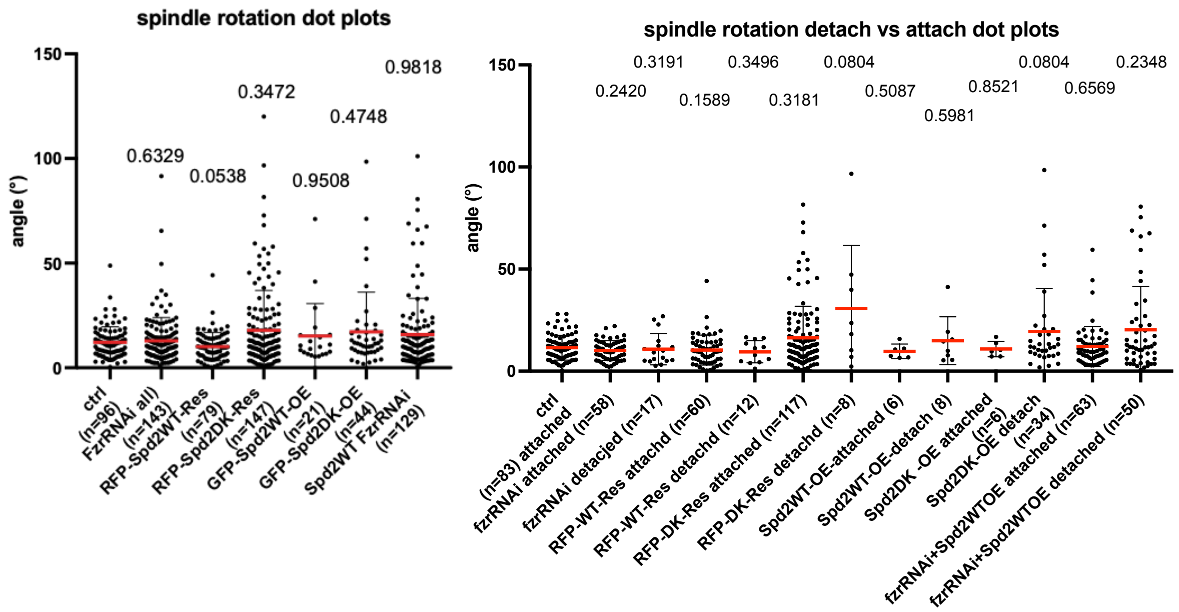

Figure 1 Spindle rotation (the deviations from the initial spindle angle at prometaphase to the angle at anaphase during mitosis, °) is not caused by altered Spd2 protein levels. Left: spindle rotations during mitosis were measured in all the NBs. Right: spindle rotations were separately analysed in NBs in which the apical centrosome was anchored to the cortex or detached in the preceding interphase. No significant changes in spindle rotations were observed between control NBs and the NBs with altered Spd2 levels. The numbers above the dots indicates the p-values of the conditions compared to control (Mann-Whitney U tests).

6. *A major problem with the conclusions is that most data are generated with the Spd2 mutants that cannot bind Fzr. In their previous manuscript, the authors report that Spd2 is essential for recruiting APC/C-Fzr to the centrosome and, in the absence of Spd2 APC/C-Fzr targets are not properly degraded. It is therefore very difficult to conclude whether the defects reported in this manuscript are due to Spd2 increased levels or lack of proper localization of APC/C-Fzr (as described in the 2016 paper). Overexpression of the wild-type Spd2 gives intermediate results and, although the authors use these results to conclude that "Fzr mislocalisation is unlikely to be the cause of the disruption of the interphase centrosome asymmetry" (line 426-427), the same results can actually be used to conclude the opposite.*

>> We thank the reviewer for mentioning this since it is very critical to distinguish our new findings from our previous report. We have strong body of evidence that the phenotypes we observed in this study is not due to Fzr mislocalisation. First, we also analysed NBs that were overexpressing wild

type Spd2 proteins (Spd2WT-OE and HA-Spd2WT-OE), which can bind Fzr, and observed the detachment of the daughter centrosome from the apical cortex as Spd2DK-OE (new Fig. 2B, Fig. 3C, D). We also showed that HA-Spd2WT OE, despite still causing apical centrosome detachment, Fzr centrosomal localisation and its asymmetric distribution were unaffected (new Fig. EV5). Finally, we observed that the incidence of the centrosome detachment phenotype correlates with estimated Spd2 protein levels in larval NBs (new Fig. 1, 2B) but not with the degrees of Fzr mislocalisation/loss (which should be highest in either Spd2DK-RES or fzrRNAi, Fig. 2B). This body of evidence strongly support our conclusion that Spd2 accumulation, but not Fzr mislocalisation, is the cause of the observed phenotypes. Nevertheless, our data cannot completely rule out completely the possibility that Fzr mislocalisation or inhibition of the activity of centrosomal Fzr may have a small contribution to the phenotypes. We included this discussion in our revised manuscript.

Minor:

7. *Table 1 could be moved to Supplementary (or removed) as the proteins as listed in Fig. 1a.*

>> Following the advice, Table 1 has been omitted from our revised manuscript.

8. *Overall, I would suggest a complete reorganization of the manuscript to avoid duplication of messages already published in previous papers, and a clear demonstration of the relevance of Spd2 levels or asymmetry with independence of its function in recruiting APC/C to the centrosomes and altering levels of centrosomal APC/C substrates*

>> Following this suggestion, we reorganised our manuscript, minimising the amount of data that may partly overlap with our previous study and clarifying the role of Spd2 as an APC/C substrate (Spd2 degradation), independent of its role in Fzr recruitment. We believe that our revised manuscript has become more clear-cut and better communicates the novelty of our findings. We are grateful for this reviewer's suggestion to help us improve our manuscript.

Referee #3:

1. *This is a huge study that contributes to the understanding of centrosome asymmetry establishment. It is somehow very difficult to follow. The choice of words and amount of text used, do not facilitate comprehension. From an experimental point of view, most results appeared obtained in a robust manner, although some require clarification and maybe a few*

experiments. A shorter version including certain clarifications would certainly increase the comprehension of this study and its impact.

I am not sure I understand the model proposed because some of the data are difficult to link.

>> We thank the reviewer for appreciating the importance and the quality of our study. We also thank this reviewer for pointing out the problem with the original manuscript and for advising how to improve our manuscript. We followed the advice and have made the manuscript into a shorter, more concise version and have revised the explanation of our model.

Major points:

1. *If increasing Spd2 levels at mitotic exit lead to mis-behaviour of centrosomes and spindles- why do the authors fail to see these in GFP-SPD2DKOE, which cannot be degraded? Like in Sup Fig2? I am asking this because to me, there seems to have fewer MTs as the signal from mChTub is different than the Ctrl.*

To answer to this reviewer's question, we look into all our data, and we could not detect any consistent decrease in mCh-Tub signals in the GFP-Spd2DK-OE condition when compared with controls. The difference that can be detected in the original Fig. S2 is likely to be due to the choice of representative images. In addition, as most of the findings were corroborated in the whole mount preparations that are more physiological relevant, we removed the squashed preparations from the new version of this manuscript.

Regarding the centrosome mis-behaviour, we do not exactly know the reason why we did not see the effect of Spd2 increase on centrosome behaviours during mitosis exit. However, one possible explanation would be that as Polo activity and levels are much higher in mitosis compared to interphase, the PCM recruitment pathway downstream of Polo is not sensitive to the increase of Spd2. Alternatively, PCM recruitment mechanisms downstream of Polo may be quite different in mitosis and interphase. For instance, Spd2 accumulation does not seem to affect Cnn levels on interphase centrosomes.

2. *The spindle mis-orientation described in Sup Fig 3. First, I would be careful with this nomenclature. Not really mis-orientation, more like not stably maintained though mitosis? In the end is in the same position at mitotic exit, as in time point 3.00 min for the entire mitosis. But what is remarkable is that the spindle is bent and very abnormal in shape. This should be quantified and described in a more detailed manner.*

>>> We appreciate this insightful comment and the suggestion by this reviewer. We regret that we used imprecise terminology to describe the observed spindle phenotypes. According to this comment, we have reanalysed the whole dataset by distinguishing “spindle rotation” (which refer to the change of the spindle angle from the initial angle established at pro/prometaphase to the spindle angle upon anaphase onset) and “division axis deviation” (which refers to the difference between the division axes, i.e., the spindle angles at telophase, in consecutive mitoses). Our data indicates that spindle rotations were not significantly affected by increased Spd2 levels (please see our response to Reviewer #2 Point 5), but division axis deviations were (new Fig. 3).

In addition, according to the suggestion by this reviewer, we performed detailed characterisation of spindle morphologies in mitotic NBs and properly documented them in new Fig. EV3. Indeed, as this reviewer pointed out, some NBs in the various genotypes used transiently exhibited various spindle abnormalities, including “bent” spindle, during mitosis (New Fig. EV3). However, these spindle abnormalities only lasted for a short period and, in most cases, the spindle recovered to the normal straight bipolar shape. As we do not know the exact cause of these phenotypes and they do not appear to be directly correlated with increasing Spd2 levels (New Fig. EV3), we decided that this mechanism would go beyond the scope of this study.

3. *In Figure 4, it is impossible to follow the mitotic spindle in the 2 lower panels.*

As in our response to Referee #1 Point 1, we have replaced the original images in Figure 4 with clearer, higher-quality images for clearer presentation (new Fig. 3B).

4. *Still on the same subject, are the authors certain that Spd2OE does not cause a spindle phenotype, in terms of morphology? If this is the case, it should be properly documented.*

As we discussed above (Point 2), we could observe transient abnormal spindle morphologies and have properly documented them in our revised manuscript (New Fig. EV3). However, we have concluded that these phenotypes were not correlated with increase Spd2 levels.

5. *If I understood this article correctly, the authors ascribe all their phenotypes in terms of centrosome defects (inheritance of the mother vs daughter) and spindle mobility, to the fact that increase SPD2-centrosome levels inhibit the recruitment of other centrosome proteins to the daughter centriole and so microtubule nucleation. But when I look at the quantifications of Cnn, or g-tubulin in fig 5 in conditions with Spd2 and Spd2DK, the levels of Cnn at the apical are exactly the same, while g-tubulin is only slightly decreased. Maybe they were not quantified at the right moment, but this actually needs to be documented.*

>>> Following the reviewer's suggestion, in our revised manuscript we have provided fuller descriptions of this analysis in Materials and Methods. In response to the reviewer's comment, we carefully chosen interphase NBs based on the DNA morphology (dispersed and clear round shape).

Indeed, the changes in the asymmetry indexes of Spd2 and γ-Tubulin were small but statistically significant (new Fig. 2E), whereas the asymmetric indexes of the other PCM tested, including Cnn, as well as Polo, showed virtually no changes (new Fig. 2E, 5B). Similar results were also obtained in HA-Spd2WT overexpression conditions (new Fig. 5D, Fig. EV5E). Indeed, it was a little surprising that Cnn was unchanged (this was reproducible. We have used two different Cnn antibodies and got same results). This may reflect different PCM structures between the active interphase centrosome and mitotic centrosomes.

The subtlety of the reductions of asymmetry levels of Spd2 and γ-Tubulin can stem from several reasons. First, the penetrance of the phenotype: in Spd2DK-RES condition, many NBs (80.8%, n = 52) still maintain active interphase daughter centrosomes (new Fig. 2B). Therefore, averaging in the whole population led to the underrepresenting of the change in asymmetry. Secondly, in our asymmetric index measurements, we made a little modification from the original method (Lerit and Rusan 2013), due to the lack of a specific marker for either daughter or mother centrosome in our assays. Consequently, although our assay can readily assess the effect of Spd2 accumulation on the degrees of asymmetric distributions between two centrosomes, it does not take into account a possible inversion of the distributions. For example, if Spd2 accumulation causes an inversion of the γ-Tubulin distribution pattern (i.e., accumulating it more at the mother centrosome, instead of the daughter centrosome), this effect would not be detected. This may also have contributed to the relative small impact on PCM asymmetry in our data.

6. *Maybe, to prove their point, it should be important to see if after microtubule depolymerization and repolymerization at mitotic exit, the Spd2DK centrosome behaviour is even worse? Or the mobility of the centrosome? Of the recovery of the soluble fraction after FRAP?*

The suggested experiments could be very insightful but were extremely hard to properly control using the whole mount brain system (*depolymerization and repolymerization by temperature shift or drugs, and analysing specific timing during at mitotic exit,* etc.). We attempted MT depolymerisation by keeping the brain samples on ice but controlling the temperature was quite hard in this setting. We also tested colcemid treatment, but did not see strong depolymerisation, which could be due to a penetrance issue. We regret that but, for this limited timeline for revision, we could not successfully conduct these new experiments.

7. *The differences in the FRAP experiments are impressive. Spd2-WT never reaches a strong signal. Is this normal? Could it be the problem in having stable Spd2, is not that the others are not recruited but rather that an unstable population is required for something that needs to be identified? Maybe documenting better the microtubule behaviour will help clarifying this point?*

>>> In FRAP experiments it is common that the fluorescent signals do not fully recover to the original level, which indicates the presence of a stable fraction, as exactly the reviewer speculated.

We acknowledge the alternative hypothesis presented by this reviewer suggesting that an unstable population of Spd2 might be doing "something" that could be causing the inactivation and detachment of the centrosome. Although this hypothesis might be very appealing, we did not find any correlation between Spd2 total levels (subsequently increased unstable population) and major defects on spindle formation (New Fig. EV3). For example, Spd2WT-OE shows a good percentage of centrosome detachment (New Fig. 2B) but does not show any clear spindle defect (New Fig. EV3). However, we cannot completely exclude any subtle defect, as the image resolution of the whole mount preparations might be a limitation to identify subtle changes on microtubule dynamics.

Minor points:

8. *The authors have to decide if it is Fzr or Cdh1. Choose one nomenclature and stick to it.*

>> Thanks for spotting this, we revised manuscript to use Fzr throughout.

9. *I count more than 55 proteins in the table provided in Fig 1a. Why is moesin added to the list? This should be explained*

We thank this reviewer for pointing out these errors. Indeed, the list in original Fig. 1 was incorrect, which contained 57 proteins (the 55 proteins in Table 1 plus L(1)dd4, another name of Grip91, and duplicated Myb). This is now corrected in the new Fig. S1.

The reason to include Moesin was that is was shown that in the Drosophila wing disc, Moesin is localised at the centrosome and regulate centrosomes (Sabino D, et al., 2015 Current Biology). As we mentioned in our response to point 7 by Reviewer #2, Table 1 has been removed in the revised manuscript.

10. *Supplementary Fig 1 legend: were translated in vitro translated. The should be ameliorated.*

>> We thank this reviewer for pointing out the mistake. We have corrected this typo.

11. *Also, the nomenclature of all the fly strains used are difficult to follow. For example, RFP-Spd2WT-Res, this refers to the WT gene tagged with RFP and in a spd2 mutant background? To only have Spd2 from the transgene. Please explain. Is this in the Spd2 def background only?*

>> We regret that we could not clearly explain the nomenclature to explain the fly lines used for our analysis. As this reviewer got right, RFP-Spd2WT-Res indeed refers to the fly strain in which RFP-fused WT Spd2 gene is expressed in a *spd2* mutant null background (the transzygotes of *spd2[z3-5711] mutant allele* and *Df(3L)BSC561*, a *spd2* d*eficiency*). We have revised the manuscript to include one full paragraph and one diagram (new Fig. 1A) to fully explain the genetic compositions of the series of the fly lines with altered Spd2 levels. We hope that these explanations can clearly communicate the genetic conditions of the fly lines used to general audience.

12. *The model in Fig 7e is impossible to follow.*

>> We regret that our explanation is unclear. We simplified and improved the model on new figure 5I and also have revised the text to explain more clearly our proposed model.

1st Revision - Editorial Decision 13th Dec 2022

Dear Dr. Kimata,

Thank you for the submission of your revised manuscript to our editorial offices. I have now received the reports from the two referees that I asked to re-evaluate your study, you will find below. As you will see, the referees now support the publication of your study in EMBO reports. Both referees have suggestions to improve the study that I ask you to address in a final revised version of the manuscript. Please also provide a final point-by-point-response addressing these points.

Moreover, I have these editorial requests:

- Please name the 'Summary' 'Abstract' and provide it written in present tense throughout.

- Please move the five keywords below the abstract.

- We now use CRediT to specify the contributions of each author in the journal submission system. CRediT replaces the author contribution section. Please use the free text box to provide more detailed descriptions during re-submission. Please do NOT add an author contributions section to the manuscript text file. See also guide to authors: https://www.embopress.org/page/journal/14693178/authorguide#authorshipguidelines

- Please make sure that the number "n" for how many independent experiments were performed, their nature (biological versus technical replicates), the bars and error bars (e.g. SEM, SD) and the test used to calculate p-values is indicated in the respective figure legends (main, EV and Appendix figures), and that statistical testing has been done where applicable. Please avoid phrases like 'independent experiment', but clearly state if these were biological or technical replicates. Please add complete statistical testing to all diagrams (main, EV and Appendix figures). Please also indicate (e.g. with n.s.) if testing was performed, but the differences are not significant. In case n=2, please show the data as separate datapoints without error bars and statistics.

Most importantly: If n<5, please show single datapoints for diagrams!

- Please add scale bars of similar style and thickness to the microscopic images (main and EV), using clearly visible black or white bars (depending on the background). Please place these in the lower right corner of the images. Please do not write on or near the bars in the image but define the size in the respective figure legend.

- We updated our journal's competing interests policy in January 2022 and request authors to consider both actual and perceived competing interests. Please review the policy https://www.embopress.org/competing-interests and update your competing interests if necessary. Please name this section 'Disclosure and Competing Interests Statement' and put it after the Acknowledgements section.

- Please order the manuscript sections like this (using these names):
Title page - Abstract - Introduction - Results & Discussion - Materials and Methods - Data availability section (DAS) - Acknowledgements - Disclosure and Competing Interests Statement - References - Figure legends - Expanded View Figure legends

- Please provide the final figure files as .eps, .tif, or .jpg (one file per figure), not as photoshop or adobe illustrator file.

- As they are significantly cropped, please provide the source data for the Western blots shown in the manuscript (including the EV figures). The source data will be published in separate source data files online along with the accepted manuscript and will be linked to the relevant figures. Please submit scans of entire gels or blots together with the revised manuscript. Please include size markers for scans of entire gels, label the scans with figure and panel number, and send one PDF file per figure (main and EV figures).

- Please provide a fully filled in author checklist (with a completed top section - author names, ms # etc.).

- Please name the EV Figures as such also in the files themselves (there they are still titled 'Figure Sx').

- Please name the movie files 'Movie EVx' and add callouts for these using these names to the manuscript text. Please upload the movie files ZIPed together with a text file containing a title and a legend.

- Finally, please find attached a word file of the manuscript text (provided by our publisher) with changes we ask you to include in your final manuscript text. Please use the attached file as basis for further revisions and provide your final manuscript file with track changes, in order that we can see any modifications done.

In addition, I would need from you:

- a short, two-sentence summary of the manuscript (not more than 35 words).
- two to four short bullet points highlighting the key findings of your study (two lines each).
- a schematic summary figure that provides a sketch of the major findings (not a data image) in jpeg or tiff format (with the exact width of 550 pixels and a height of not more than 400 pixels) that can be used as a visual synopsis on our website.

I look forward to seeing the final revised version of your manuscript when it is ready. Please let me know if you have questions regarding the revision.

Please use this link to submit your revision: https://embor.msubmit.net/cgi-bin/main.plex

Best,

Achim Breiling
Senior Editor
EMBO Reports

Referee #1:

The revised version of the article by Meghini and colleagues has improved substantially. Most of my comments, have been taken into account. I think the paper can be considered for publication. It contains a large amount of data and the overall message is quite interesting.

I would recommend that the authors still pay attention to the text, it is too long, references are not always correct and certain statements may be improved. For example, in the abstract- " Proper functioning of the centrosome is vital for the development and physiology of animals". Not sure about this statement in flies, the model organism used here, where centrosomes appear to be dispensable for most of fly development even if essential in adults for cilia assembly and function in adults. And what does "proper" mean?
Certain figures also are difficult to follow with the magnifications on top of the cells. Maybe including dashed circles that can be more easily seen in NB cell surrounding will also be helpful. In certain figures they are almost undetectable because they are too thin.

Referee #2:

The authors have really improved the manuscript by focusing to the specific points that were novel and analyzing with a deeper detail the cellular phenotypes resulting from Spd2 accumulation. The revised version of the manuscript suggests that the regulation of Spd2 levels by APC/C, previously reported by the team, controls centrosome dynamics in Drosophila neural stem cells, which is now the focus of the manuscript. I still wonder whether the two activities, targeting of the APC/C to the centrosome and degradation of Spd2, are physiologically relevant or the phenotypes are mixed up. Yet, I don't have further technical comments on the revised version and the manuscript could be published without additional revision.

Response to reviewers

Dear Editor,

First, we would like to thank the reviewers for their time and consideration. We appreciated all their constructive criticisms and useful suggestions, and we are very happy to have your support for the publication of our manuscript in EMBO Reports. Following their advice, we have revised our manuscript carefully and we believe that our new manuscript is now fully ready for publication.
Below, we provide point by point response to the reviewers' comments.

Referee #1

1. *I would recommend that the authors still pay attention to the text, it is too long*
   - We revised text and carefully removed all redundant parts and unnecessary details to make our manuscript shorter and more concise.
2. *references are not always correct.*
   - We carefully examined the references and indeed found some references to be inaccurate, which have been corrected in the final version. We thank the reviewer.
3. *certain statements may be improved. For example, in the abstract- " Proper functioning of the centrosome is vital for the development and physiology of animals". Not sure about this statement in flies, the model organism used here, where centrosomes appear to be dispensable for most of fly development even if essential in adults for cilia assembly and function in adults. And what does "proper" mean?*
   - We thank this reviewer for pointing this out. Indeed, "proper" is a vague word and therefore has been replaced with "normal" in the final manuscript.
     Although what level of function is considered "important" can be subjective, we think the centrosome is indeed critically important for Drosophila biology. The importance of the centrosome for Drosophila development is undermined by a misconception led by Basto et al., 2006, in which the authors did not take into consideration of a maternal effect (early development is supported by maternally deposited proteins), only examined centrioles but not PCM, and did not examine many tissues. In fact, the centrosome is essential for syncytial division in early embryos and for male meiotic divisions, and is required for the accuracy/fidelity of asymmetric cell division of neuroblasts and epithelial division of the imaginal wing disc (reviewed by Tillery et al., 2018). Also, although "centrioles" are not essential for spindle assembly of most somatic cells, PCM such as γ-Tubulin is
4. *Certain figures also are difficult to follow with the magnifications on top of the cells.* Maybe including dashed circles that can be more easily seen in NB cell surrounding will also be helpful. In certain figures they are almost undetectable because they are too thin.
   - We thank this reviewers for pointing out these flaws and offering suggestions. Following the reviewer's advice we have modified the relevant figures. We made the outlines of NBs thicker and more visible. Also, although, due to space shortage, we could not avoid overlaying the cells with insets, they were made smaller to minimise overlaps.

Referee #2

1. *I still wonder whether the two activities, targeting of the APC/C to the centrosome and degradation of Spd2, are physiologically relevant or the phenotypes are mixed up.*
   - We show that the centrosomal localisation of Fzr is not affected upon Spd2 overexpression (Fig. EV5). However, as this reviewer mentioned, we cannot rule out the possibility that some of the phenotypes we observed may be due to a combined effect of Spd2 accumulation and APC/C mislocalisation. It is an intriguing open question how the two functions of Spd2 as an APC/C regulator and substrate are coordinated and related, which we discuss in the Discussion section.

Dr. Yuu Kimata
ShanghaiTech University
School of Life Science and Technology
393 Middle Huaxia Road
Pudong Distric
Shanghai, Shanghai 201210
China

Dear Dr. Kimata,

I am very pleased to accept your manuscript for publication in the next available issue of EMBO reports. Thank you for your contribution to our journal.

At the end of this email I include important information about how to proceed. Please ensure that you take the time to read the information and complete and return the necessary forms to allow us to publish your manuscript as quickly as possible.

As part of the EMBO publication's Transparent Editorial Process, EMBO reports publishes online a Review Process File to accompany accepted manuscripts. As you are aware, this File will be published in conjunction with your paper and will include the referee reports, your point-by-point response and all pertinent correspondence relating to the manuscript.

If you do NOT want this File to be published, please inform the editorial office within 2 days, if you have not done so already, otherwise the File will be published by default [contact: emboreports@embo.org]. If you do opt out, the Review Process File link will point to the following statement: "No Review Process File is available with this article, as the authors have chosen not to make the review process public in this case." Please note that the author checklist will still be published even if you opt out of the transparent process.

Thank you again for your contribution to EMBO reports and congratulations on a successful publication. Please consider us again in the future for your most exciting work.

Yours sincerely,

Achim Breiling
Editor
EMBO Reports

****

THINGS TO DO NOW:

Once your article has been received by Wiley for production, the corresponding author will receive an email from Wiley's Author Services system which will ask them to log in and will present them with the appropriate license for completion.

You will receive proofs by e-mail approximately 2-3 weeks after all relevant files have been sent to our Production Office; you should return your corrections within 2 days of receiving the proofs.

Please inform us if there is likely to be any difficulty in reaching you at the above address at that time. Failure to meet our deadlines may result in a delay of publication, or publication without your corrections.

All further communications concerning your paper should quote reference number EMBOR-2022-55607V3 and be addressed to emboreports@wiley.com.

Should you be planning a Press Release on your article, please get in contact with emboreports@wiley.com as early as possible, in order to coordinate publication and release dates.

EMBO Press Author Checklist

Corresponding Author Name: Yuu Kimata
Journal Submitted to: EMBO Reports
Manuscript Number: EMBOR-2022-55607V2

USEFUL LINKS FOR COMPLETING THIS FORM
The EMBO Journal - Author Guidelines
EMBO Reports - Author Guidelines
Molecular Systems Biology - Author Guidelines
EMBO Molecular Medicine - Author Guidelines

Reporting Checklist for Life Science Articles (updated January 2022)
This checklist is adapted from Materials Design Analysis Reporting (MDAR) Checklist for Authors. MDAR establishes a minimum set of requirements in transparent reporting in the life sciences (see Statement of Task: 10.31222/osf.io/9sm4x). Please follow the journal's guidelines in preparing your manuscript.
Please note that a copy of this checklist will be published alongside your article.

Abridged guidelines for figures
1. Data
The data shown in figures should satisfy the following conditions:
- the data were obtained and processed according to the field's best practice and are presented to reflect the results of the experiments in an accurate and unbiased manner.
- ideally, figure panels should include only measurements that are directly comparable to each other and obtained with the same assay.
- plots include clearly labeled error bars for independent experiments and sample sizes. Unless justified, error bars should not be shown for technical replicates.
- if n<5, the individual data points from each experiment should be plotted. Any statistical test employed should be justified.
- Source Data should be included to report the data underlying figures according to the guidelines set out in the authorship guidelines on Data Presentation.

2. Captions
Each figure caption should contain the following information, for each panel where they are relevant:
- a specification of the experimental system investigated (eg cell line, species name).
- the assay(s) and method(s) used to carry out the reported observations and measurements.
- an explicit mention of the biological and chemical entity(ies) that are being measured.
- an explicit mention of the biological and chemical entity(ies) that are altered/varied/perturbed in a controlled manner.
- the exact sample size (n) for each experimental group/condition, given as a number, not a range;
- a description of the sample collection allowing the reader to understand whether the samples represent technical or biological replicates (including how many animals, litters, cultures, etc.).
- a statement of how many times the experiment shown was independently replicated in the laboratory.
- definitions of statistical methods and measures:
  - common tests, such as t-test (please specify whether paired vs. unpaired), simple χ2 tests, Wilcoxon and Mann-Whitney tests, can be unambiguously identified by name only, but more complex techniques should be described in the methods section;
  - are tests one-sided or two-sided?
  - are there adjustments for multiple comparisons?
  - exact statistical test results, e.g., P values = x but not P values < x;
  - definition of 'center values' as median or average;
  - definition of error bars as s.d. or s.e.m.

Please complete ALL of the questions below.
Select "Not Applicable" only when the requested information is not relevant for your study.

Materials

Newly Created Materials	Information included in the manuscript?	In which section is the information available? (Reagents and Tools Table, Materials and Methods, Figures, Data Availability Section)
New materials and reagents need to be available; do any restrictions apply?	Not Applicable	

Antibodies	Information included in the manuscript?	In which section is the information available? (Reagents and Tools Table, Materials and Methods, Figures, Data Availability Section)
For **antibodies** provide the following information:<br>- Commercial antibodies: RRID (if possible) or supplier name, catalogue number and or/clone number<br>- Non-commercial: RRID or citation	Yes	Materials and Methods

DNA and RNA sequences	Information included in the manuscript?	In which section is the information available? (Reagents and Tools Table, Materials and Methods, Figures, Data Availability Section)
**Short novel DNA or RNA including primers, probes**: provide the sequences.	Yes	Materials and Methods

Cell materials	Information included in the manuscript?	In which section is the information available? (Reagents and Tools Table, Materials and Methods, Figures, Data Availability Section)
**Cell lines:** Provide species information, strain. Provide accession number in repository **OR** supplier name, catalog number, clone number, and/**OR** RRID.	Yes	Materials and Methods
**Primary cultures:** Provide species, strain, sex of origin, genetic modification status.	Not Applicable	
Report if the cell lines were recently **authenticated** (e.g., by STR profiling) and tested for mycoplasma contamination.	Not Applicable	

Experimental animals	Information included in the manuscript?	In which section is the information available? (Reagents and Tools Table, Materials and Methods, Figures, Data Availability Section)
**Laboratory animals or Model organisms:** Provide species, strain, sex, age, genetic modification status. Provide accession number in repository **OR** supplier name, catalog number, clone number, **OR** RRID.	Yes	Materials and Methods
**Animal observed in or captured from the field:** Provide species, sex, and age where possible.	Not Applicable	
Please detail **housing and husbandry conditions**.	Not Applicable	

Plants and microbes	Information included in the manuscript?	In which section is the information available? (Reagents and Tools Table, Materials and Methods, Figures, Data Availability Section)
**Plants:** provide species and strain, ecotype and cultivar where relevant, unique accession number if available, and source (including location for collected wild specimens).	Not Applicable	
**Microbes:** provide species and strain, unique accession number if available, and source.	Not Applicable	

Human research participants	Information included in the manuscript?	In which section is the information available? (Reagents and Tools Table, Materials and Methods, Figures, Data Availability Section)
If collected and within the bounds of privacy constraints report on age, sex and gender or ethnicity for all study participants.	Not Applicable	

Core facilities	Information included in the manuscript?	In which section is the information available? (Reagents and Tools Table, Materials and Methods, Figures, Data Availability Section)
If your work benefited from core facilities, was their service mentioned in the acknowledgments section?	Yes	

Design

Study protocol	Information included in the manuscript?	In which section is the information available? (Reagents and Tools Table, Materials and Methods, Figures, Data Availability Section)
If study protocol has been **pre-registered, provide DOI in the manuscript**. For clinical trials, provide the trial registration number **OR** cite DOI.	Not Applicable	
Report the **clinical trial registration number** (at ClinicalTrials.gov or equivalent), where applicable.	Not Applicable	

Laboratory protocol	Information included in the manuscript?	In which section is the information available? (Reagents and Tools Table, Materials and Methods, Figures, Data Availability Section)
Provide DOI OR other citation details if **external detailed step-by-step protocols** are available.	Not Applicable	

Experimental study design and statistics	Information included in the manuscript?	In which section is the information available? (Reagents and Tools Table, Materials and Methods, Figures, Data Availability Section)
Include a statement about **sample size** estimate even if no statistical methods were used.	Not Applicable	
Were any steps taken to minimize the effects of subjective bias when allocating animals/samples to treatment (e.g. **randomization procedure**)? If yes, have they been described?	Not Applicable	
Include a statement about **blinding** even if no blinding was done.	Not Applicable	
Describe **inclusion/exclusion criteria** if samples or animals were excluded from the analysis. Were the criteria pre-established?<br><br>If sample or data points were omitted from analysis, report if this was due to attrition or intentional exclusion and provide justification.	Not Applicable	
For every figure, are **statistical tests** justified as appropriate? Do the data meet the assumptions of the tests (e.g., normal distribution)? Describe any methods used to assess it. Is there an estimate of variation within each group of data? Is the variance similar between the groups that are being statistically compared?	Not Applicable	

Sample definition and in-laboratory replication	Information included in the manuscript?	In which section is the information available? (Reagents and Tools Table, Materials and Methods, Figures, Data Availability Section)
In the figure legends: state number of times the experiment was **replicated** in laboratory.	Yes	
In the figure legends: define whether data describe **technical or biological replicates**.	Yes	

Ethics

Ethics	Information included in the manuscript?	In which section is the information available? (Reagents and Tools Table, Materials and Methods, Figures, Data Availability Section)
Studies involving **human participants**: State details of **authority granting ethics approval** (IRB or equivalent committee(s), provide reference number for approval.	Not Applicable	
Studies involving **human participants**: Include a statement confirming that **informed consent** was obtained from all subjects and that the experiments conformed to the principles set out in the WMA Declaration of Helsinki and the Department of Health and Human Services Belmont Report.	Not Applicable	
Studies involving **human participants:** For publication of **patient photos**, include a statement confirming that consent to publish was obtained.	Not Applicable	
Studies involving experimental **animals**: State details of **authority granting ethics approva**l (IRB or equivalent committee(s), provide reference number for approval. Include a statement of compliance with ethical regulations.	Not Applicable	
Studies involving **specimen and field samples:** State if relevant **permits** obtained, provide details of authority approving study; if none were required, explain why.	Not Applicable	

Dual Use Research of Concern (DURC)	Information included in the manuscript?	In which section is the information available? (Reagents and Tools Table, Materials and Methods, Figures, Data Availability Section)
Could your study fall under dual use research restrictions? Please check biosecurity documents and list of **select agents and toxins** (CDC): https://www.selectagents.gov/sat/list.htm	Not Applicable	
If you used a select agent, is the security level of the lab appropriate and reported in the manuscript?	Not Applicable	
If a study is subject to dual use research of concern regulations, is the name of the **authority granting approval and reference number** for the regulatory approval provided in the manuscript?	Not Applicable	

Reporting

The MDAR framework recommends adoption of discipline-specific guidelines, established and endorsed through community initiatives. Journals have their own policy about requiring specific guidelines and recommendations to complement MDAR.

Adherence to community standards	Information included in the manuscript?	In which section is the information available? (Reagents and Tools Table, Materials and Methods, Figures, Data Availability Section)
State if relevant guidelines or checklists (e.g., **ICMJE, MIBBI, ARRIVE, PRISMA**) have been followed or provided.	Not Applicable	
For **tumor marker prognostic studies**, we recommend that you follow the **REMARK** reporting guidelines (see link list at top right). See author guidelines, under 'Reporting Guidelines'. Please confirm you have followed these guidelines.	Not Applicable	
For **phase II and III randomized controlled trials**, please refer to the **CONSORT** flow diagram (see link list at top right) and submit the CONSORT checklist (see link list at top right) with your submission. See author guidelines, under 'Reporting Guidelines'. Please confirm you have submitted this list.	Not Applicable	

Data Availability

Data availability	Information included in the manuscript?	In which section is the information available? (Reagents and Tools Table, Materials and Methods, Figures, Data Availability Section)
Have **primary datasets** been deposited according to the journal's guidelines (see 'Data Deposition' section) and the respective accession numbers provided in the Data Availability Section?	Not Applicable	
Were **human clinical and genomic datasets** deposited in a public access-controlled repository in accordance to ethical obligations to the patients and to the applicable consent agreement?	Not Applicable	
Are **computational models** that are central and integral to a study available without restrictions in a machine-readable form? Were the relevant accession numbers or links provided?	Not Applicable	
If publicly available data were reused, provide the respective **data citations in the reference list**.	Not Applicable	
